# Design of a Novel Peptide‐Based Vaccine Targeting *Streptococcus mutans* SpaP Antigen for Dental Caries Prevention

**DOI:** 10.1155/ijod/5545020

**Published:** 2026-06-29

**Authors:** Fazilat Khademi, Amir Taherkhani, Ebrahim Yarmohammadi

**Affiliations:** ^1^ Department of Restorative Dentistry, School of Dentistry, Dental Research Center, Hamadan University of Medical Sciences, Hamadan, Iran, umsha.ac.ir; ^2^ Research Center for Molecular Medicine, Institute of Cancer, Hamadan University of Medical Sciences, Hamadan, Iran, umsha.ac.ir; ^3^ Urology and Nephrology Research Center, Avicenna Institute of Clinical Sciences, Hamadan University of Medical Sciences, Hamadan, Iran, umsha.ac.ir

**Keywords:** Ag I/II, immunotherapy, multiepitope, oral immunity, reverse vaccinology

## Abstract

**Objective:**

Dental caries is a widespread oral health issue affecting both children and adults. *Streptococcus mutans* (*S. mutans*) is a key bacterium responsible for its development. The Antigen I/II (Ag I/II; SpaP) protein of *S. mutans* is a promising target for a caries vaccine. Although SpaP is a validated vaccine target, conventional approaches lack epitope‐level precision and rational adjuvant integration. The present study addresses this gap by computationally designing a multiepitope vaccine (MEV) targeting only the most immunogenic regions of SpaP.

**Methods:**

The SpaP protein sequence was retrieved from UniProtKB. Adjuvants, a TLR4 agonist from *Mycobacterium tuberculosis*, and the PADRE peptide were incorporated to enhance the immune response. Potential T‐cell and B‐cell epitopes (BCEs) were predicted using the IEDB server. They were evaluated for their ability to trigger an immune response, lack of allergenicity, and binding strength to human and mouse MHC molecules. Using appropriate linkers, selected epitopes were combined into an MEV construct. Computational analyses assessed the vaccine’s properties, 3D structure, and interaction with Toll‐like receptor‐4 (TLR4).

**Results:**

Six MHC‐I, six MHC‐II, and six BCEs were indicated from SpaP, for a total of 18 epitopes selected for vaccine design. Some of these have been experimentally validated according to IEDB. This vaccine indicated potential for favorable biochemical properties, including high stability, solubility, and antigenicity. Molecular docking predicted strong vaccine–TLR4 binding affinity, with a Gibbs free energy of binding of −315.77 kcal/mol. Normal mode analysis (NMA) further suggested the stability of the vaccine‐TLR4 complex and showed minimal structural changes. This study presents a novel approach to the design of SpaP‐targeted vaccines.

**Conclusion:**

This computationally designed MEV represents a promising candidate for experimental validation and offers a cost‐effective, rational framework for developing next‐generation dental caries vaccines with enhanced immunogenicity and broader protective coverage.

## 1. Introduction

Dental caries has been established as one of the most pervasive oral health conditions observed across pediatric and adult populations [1–3]. Statistical evidence indicates that oral diseases affect ~3.7 billion individuals worldwide, of whom 2.37 billion have caries in their permanent dentition [4, 5]. By the World Health Organization’s definition, dental caries is characterized by the deterioration of dental enamel due to acidic byproducts produced by bacterial metabolism of sugars [6]. This persistent disease burden underscores the need for antigen‐specific preventive strategies.

The pathogenesis of dental caries begins when dietary sugars are metabolized by oral microorganisms, leading to the production of acids that facilitate the dissolution of dental enamel and dentin. The etiology of dental caries has been attributed to multiple factors, with retained food debris on dental surfaces and in interproximal spaces identified as a primary contributor. Cariogenic substances, particularly those rich in glucose, such as carbohydrates, confectionery, and various sweetened products, are implicated in plaque biofilm formation on dental surfaces, which subsequently serve as a colonization medium for diverse microorganisms [7].

*Streptococcus* has been identified as the predominant bacterial group within the oral microbiome, with *Streptococcus mutans* (*S. mutans*) being recognized as a primary etiological agent in caries development. *S. mutans* colonization leads to the deterioration of dental hard tissues by fermenting carbohydrates (including sucrose and fructose) into lactic acid and other organic acids [8]. The progressive demineralization of dental enamel and reduction in salivary pH are attributed to the lactic acid generated by *S. mutans* within the plaque biofilm [9]. The pathogenicity of *S. mutans* is attributed to its capacity to synthesize extracellular polysaccharides through glucose transferase activity, which facilitates bacterial adherence and aggregation, ultimately leading to the establishment of plaque biofilm and subsequent oral colonization. The cariogenic potential of *S. mutans* is further enhanced by its ability to metabolize carbohydrates into acids that initiate dental tissue demineralization and cavity formation. Therefore, there is an urgent need for targeted vaccine strategies directed against key virulence factors of *S. mutans*.

Multiple surface proteins, including antigen I/II (alternatively designated as SpaP, Pac, and P1), glucan‐binding proteins, a collagen‐binding protein, and glycosyltransferases are synthesized by *S. mutans*, thereby coordinating dental plaque formation [10]. Two distinct adhesion mechanisms have been identified in *S. mutans*: sucrose‐dependent (where glycosyltransferases are essential) and sucrose‐independent (where Ag I/II is necessary) [11]. Under sucrose‐deficient conditions, *S. mutans* produces various crucial adhesins, including antigen I/II. This protein is bound explicitly to salivary agglutinin [12], which has been implicated in bacterial tooth adhesion [13] and biofilm development. This relationship has been substantiated by observations showing that Ag I/II‐deficient mutants produced 65% less biofilm than wild‐type strains [14], accompanied by diminished capacity for aggregation and collagen‐dependent tooth dentin invasion [10, 14]. The virulence properties of Ag I/II have been extensively investigated through gnotobiotic rat models [15], and this antigen has been identified as a promising candidate for anticaries vaccine development [16–20]. Besides, conventional caries prevention approaches were inadequate for optimal caries management. Consequently, developing alternative therapeutic regimens that complement existing preventive strategies has been deemed highly necessary [21].

Reverse vaccinology systematically identifies vaccine candidates from genomic and proteomic data. It predicts antigenic epitopes that trigger B‐cell and T‐cell responses, supporting the design of broad‐protection MEVs [22, 23]. Despite the recognized role of *S. mutans* in dental caries and the importance of its surface protein antigen SpaP in bacterial adhesion, the development of epitope‐focused vaccines targeting this antigen remains limited. To date, no comprehensive design or evaluation of a SpaP‐based multiepitope vaccine (MEV) has been reported. Therefore, identifying immunogenic SpaP epitopes using computational vaccine design strategies may offer a promising approach to developing an effective preventive vaccine against dental caries. The present study addresses this gap by designing a novel peptide‐based vaccine targeting SpaP.

**Figure 1:**
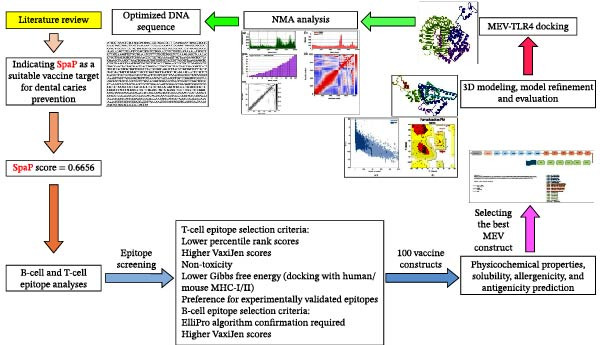
Graphical abstract of the study methodology. Abbreviations: MEV, multiepitope vaccine; NMA, normal mode analysis; SpaP, surface protein antigen P; TLR4, Toll‐like receptor 4.

Therefore, this study had three main objectives: (1) to identify highly immunogenic, nonallergenic, and stable T‐cell and B‐cell epitopes (BCEs) from the SpaP protein; (2) to construct a MEV incorporating these epitopes with appropriate adjuvants and linkers; and (3) to evaluate the vaccine’s physicochemical properties, structural stability, binding affinity to TLR4, and codon optimization for *E. coli* expression using computational tools. The precise selection of antigens, epitopes, adjuvants, and linkers fundamentally influences the therapeutic efficacy of vaccine design. The drug design process has been significantly streamlined through bioinformatics, enabling the identification of optimal antigens and potential epitopes, thereby reducing costs, minimizing risks, and shortening development timelines. The prediction of immunogenic epitopes from target antigens is facilitated by immunoinformatics, a specialized subdivision of bioinformatics [24]. Incorporating adjuvants can enhance immune responses, among which TLR agonists have been particularly efficacious. This is exemplified by the TLR4 agonist extracted from *Mycobacterium tuberculosis* (*M. tuberculosis*), which has been shown to exert powerful immunomodulatory effects, thereby establishing its potential as a promising adjuvant candidate for vaccine development [25]. The overall methodology of this study was based on that employed by Bayat et al. [26], Baghaei et al. [27], and Jalalvand et al. [28]. The workflow of the present study is shown in Figure 1.

## 2. Methods

### 2.1. Study Design

This study did not involve human participants, clinical data, or observational methods; therefore, the STROBE guidelines for reporting observational studies do not apply to this computational immunoinformatics workflow.

### 2.2. SpaP and Adjuvant Sequences

The entire amino acid sequence of the SpaP antigen, consisting of 1565 residues, was retrieved from the UniProtKB database (https://www.uniprot.org/uniprotkb/P11657/entry) (Supporting Information 1: Data 1). In addition, the vaccine’s efficacy was enhanced by adding the 50S ribosomal protein L7/L12 from *M. tuberculosis* and a universal T‐helper epitope (PADRE sequence: AKFVAAWTLKAAA) to augment immunogenicity [29]. The potent interaction between the 50S ribosomal protein L7/L12 and TLR4, a key pattern recognition receptor that initiates innate immunity upon detecting pathogen‐associated molecular patterns, has been well established in numerous studies. The activation of innate response has been demonstrated through this sequence’s agonistic interaction with TLR4 [30, 31]. Additionally, the PADRE epitope can effectively address HLA‐DR polymorphisms across populations as it binds to 15 of the 16 most prevalent human HLA‐DR types [32].

### 2.3. T Cell Epitope Prediction

MHC class I and II epitopes were predicted via the IEDB server (https://tools.iedb.org/mhci/) [33]. This publicly accessible repository compiles experimental data on antibody and T‐cell epitopes across species for infectious diseases, allergies, autoimmunity, and transplantation. The IEDB server computed percentile ranks for each epitope based on the most common human and mouse MHC class I and II alleles. Although vaccine efficacy was not tested in mice, the epitope binding affinity to murine MHC I and MHC II was analyzed. The goal was to engineer a vaccine with epitopes that bind robustly to MHC I and MHC II in both humans and mice, a key factor for potential efficacy and immunization advancement [34].

A percentile rank <1.0 was chosen for MHC alleles as this criterion is associated with high binding affinity and corresponds to the top 1% of predicted epitopes. This stringent criterion was used to identify epitopes with the highest probability of eliciting potent T‐cell responses.

Additionally, experimentally validated SpaP epitopes from IEDB (Supporting Information 2: Data 2) were included in the final vaccine due to their demonstrated immunostimulatory capacity. The final MEV was developed by integrating both predicted and experimentally validated epitope annotations from the IEDB database. It should be noted that the most promising candidates among the IEDB’s experimentally supported entries were subjected to comprehensive screening through various algorithms, as will be elaborated in the following section [26].

### 2.4. Linear B‐Cell Epitope Prediction

B‐cell‐interacting epitopes were identified using the IEDB server. A comprehensive suite of methodologies was employed to predict linear BCEs based on SpaP sequence characteristics, utilizing amino acid scales and the Hidden Markov Model. These methodologies encompassed Chou & Fasman Beta‐Turn, Emini Surface Accessibility, Karplus & Schulz Flexibility, Kolaskar & Tongaonkar Antigenicity, Parker Hydrophilicity, and Bepipred Prediction methods. Subsequently, BCEs within the three‐dimensional structure of SpaP were determined using the ElliPro tool, with analyses based on solvent accessibility and flexibility parameters. This analysis was conducted following homology modeling and model refinement procedures, utilizing the SwissModel [35] and Modelrefiner servers (https://zhanggroup.org/ModRefine).

Three principal criteria governed the selection of definitive BCEs: (1) the epitope must have been identified by at least five of the prediction methods described earlier; (2) confirmation through the ElliPro tool was required; and (3) experimental validation based on the IEDB database was deemed necessary. From the pool of epitopes meeting these stringent criteria, those with superior Vaxijen scores were selected for adding to the vaccine structure.

### 2.5. T‐Cell Epitope Screening

A comprehensive screening methodology was implemented to elucidate optimal epitopes for CD8^+^ and CD4^+^ through the following systematic process:

Epitopes with human MHC percentile ranks >1.0 were first excluded. The retained epitopes were then analyzed for antigenicity using VaxiJen v2.0 and assessed for toxicity using the ToxinPred server [36]. Those epitopes determined to be nonantigenic or potentially toxic were ignored for further analyses. Epitopes that had received IEDB experimental support or illustrated a rank score <1.0 against murine MHC alleles were preserved for continued investigation.

The subsequent analysis was bifurcated into two distinct methodological approaches:

The primary approach was predicated exclusively on predictive methods. Epitopes exhibiting a rank score <1.0 against murine MHC molecules were subjected to docking analysis. This analysis was conducted via the HPEPDOCK 2.0 server [37] against human MHC‐I alleles (HLA‐B3501 and HLA‐A0201) and murine MHC‐I molecules (H2‐Dd, H2‐Kd, and H2‐Ld). The final selection was determined by evaluating the Gibbs free energy of binding and Vaxijen antigenicity scores.

The secondary method focused on experimentally derived epitope data in the IEDB, selecting optimal candidates using the aforementioned algorithmic criteria. For these epitopes, a percentile rank <1.0 for murine MHC alleles was not considered necessary.

### 2.6. Engineering Vaccine Constructs

The most promising vaccine components were identified by selecting high‐ranking cytotoxic T lymphocytes (CTL), helper T lymphocytes (HTL), and BCEs from the screening phase. These epitopes were assembled into cohesive constructs through strategically selected linker sequences.

A 130‐residue primary adjuvant was linked at the N‐terminus to the “PADRE” secondary adjuvant via an “EAAAK” linker. The “PADRE” sequence was subsequently conjugated to the initial CTL epitope throughthea “PMGLP” linker sequence. The PMGLP linker was used between PADRE and the first epitope because it is cleaved by cathepsin S, a key endosomal protease in MHC class II presentation. The EAAAK linker helps induce robust cellular and humoral immune responses against specific antigens while also enhancing the vaccine’s stability and longevity. The AAY linker was employed between CTL epitopes, as it is commonly associated with proteasomal cleavage, influences protein stability, and enhances epitope presentation. The glycine–proline linker (GPGPG) prevents junctional epitopes and aids immune processing. Additionally, the bi‐lysine (KK) linker was used to preserve the vaccine construct’s independent immunogenicity [38–43].

To ensure a comprehensive evaluation, a heterogeneous collection of 100 vaccine sequences was generated through random assembly. These vaccine candidates then underwent rigorous evaluation.

### 2.7. Allergenicity, Antigenicity, Solubility, and Physicochemical Properties of Vaccine Constructs

The allergenicity assessment of the vaccine constructs was conducted through the AllerCatPro 2.0 server [44]. The constructs’ antigenicity was evaluated using the Vaxijen v2.0 server to analyze their primary sequences.

The solubility criteria of the vaccine candidates were evaluated through the SOLpro tool [45]. Comprehensive physicochemical characterization was also executed utilizing the ProtParam web server [46]. Multiple parameters were systematically analyzed, including:

• The instability index
• The aliphatic index
• The theoretical isoelectric point (pI)
• The half‐life in mammalian cellular environments
• The grand average of hydropathicity (GRAVY)

### 2.8. Tertiary Structure Modeling and Refinement

The optimal vaccine construct’s three‐dimensional (3D) structural coordinates were determined using the I‐TASSER online server [47]. Further structural enhancement of the vaccine candidate’s 3D model was achieved using the Galaxy Refine server [48], which employs a sophisticated refinement methodology validated by the CASP10 experiment.

### 2.9. Model Evaluation

The MEV’s 3D structural conformation was thoroughly evaluated using two online servers: ProSA‐web [48] and PROCHECK [49]. The structural quality assessment was primarily conducted using ProSA‐web, an advanced analytical tool that evaluates protein structure scores within the context of established protein structural databases. This analysis was further augmented by PROCHECK, which enabled a detailed stereochemical characterization. The PROCHECK analysis yielded comprehensive outputs in two primary forms: graphically rendered PostScript representations and detailed residue‐specific analytical reports, thereby facilitating an extensive examination of the structural characteristics.

### 2.10. Molecular Docking

The binding affinity between TLR4 and vaccine was evaluated using molecular docking with the HDOCK server [50]. The docking simulation used the refined vaccine model and the 3D TLR4 structure (PDB ID: 4G8A) as the receptor.

### 2.11. Normal Mode Analysis

The coarse‐grained approximation of the flexibility of the vaccine‐TLR4 complex was investigated through normal mode analysis (NMA) executed via the iMOD server. Advanced NMA algorithms were implemented in the iMODS platform, enabling comprehensive insights into complex conformational transitions and dynamic properties [51, 52].

### 2.12. Reverse Transcription and Codon Optimization

The nucleotide sequence of the vaccine was generated through reverse translation utilizing the SMS web server [53]. Subsequently, codon optimization was performed through the Java Codon Adaptation Tool (JCAT) [54]. Thus, an optimized nucleotide sequence for the vaccine was derived, facilitating efficient expression and translation within the target host system.

### 2.13. A Vector Design

The target MEV gene was codon‐optimized for *E. coli* expression and inserted at the *NcoI* and *HindIII* sites for directional cloning. The pET28a(+) vector was used for in silico cloning and expression, providing an IPTG‐inducible T7 promoter and a C‐terminal 6×His tag for purification. The complete pET28a‐MEV construct was designed and visualized using SnapGene.

## 3. Results

### 3.1. T‐Cell Epitopes

The SpaP contained multiple high‐affinity T‐cell epitopes that bound both human and murine MHC molecules, supporting the feasibility of cross‐species vaccine evaluation in mouse models.

The analysis encompassed 6180 unique MHC‐I‐specific sequences of 9–10 amino acids in length and 58,956 MHC‐II‐specific sequences spanning 14–15 amino acids. MHC class I and II peptides with percentile rank scores <1 are listed in Supporting Information 3: Data 3 and Supporting Information 4: Data 4, respectively.

Analysis revealed six exceptional MHC‐I epitope sequences that met stringent predictive criteria and were selected for the vaccine design. These sequences were NYYELTWDL, TVHFHYFKL, DPTVHFHYF, AYGIKSNVV, STNYYELTW, and TYKNNFTLTV. Notably, each peptide sequence achieved rank scores <1.0 in murine MHC‐I evaluations. Among these, AYGIKSNVV received additional confirmation through experimentally validated epitopes reported in the IEDB database. The significance of these candidates was further supported by their favorable characteristics, including Vaxijen scores exceeding 1.0 and Gibbs free energy of binding below −200 kcal/mol (Table 1).

**Table 1:** MHC‐I epitopes.

Alleles with percentile rank score <1	Start	End	Length	Peptide	The most favorable percentile rank for alleles	Vaxijen score	Found in experimental data (Yes/No)?	Binding to the MHC‐I in mice, based on rank scores?	Docking score with human MHC‐I (HLA‐A0201) PDB ID, 6TDR	Docking score with human MHC‐I (HLA‐B35) PDB ID, 3LKN	Docking score with mice MHC‐I (H2‐Kd) PDB ID, 5GSV	Docking score with mice MHC‐I (H2‐Ld) PDB ID, 1LDP	Docking score with mice MHC‐I (H2‐Dd) PDB ID, 5IVX
HLA‐A ^∗^24:02; HLA‐B ^∗^38:01	41	49	9	NYYELTWDL	0.14	1.70	No	Yes	−256.476	−241.131	−241.869	−264.156	−216.554
HLA‐B ^∗^08:01; HLA‐A ^∗^26:01	29	37	9	TVHFHYFKL	0.48	1.60	No	Yes	−278.319	−251.944	−253.86	−321.746	−247.633
HLA‐B ^∗^35:01; HLA‐B ^∗^51:01; HLA‐B ^∗^08:01; HLA‐B ^∗^18:01	27	35	9	DPTVHFHYF	0.12	1.36	No	Yes	−302.882	−256.305	−255.996	−305.713	−247.945
HLA‐A ^∗^24:02	49	57	9	AYGIKSNVV	0.94	1.30	Yes	Yes	−228.951	−229.55	−211.54	−225.578	−207.215
HLA‐B ^∗^58:01	39	47	9	STNYYELTW	0.01	1.19	No	Yes	−254.728	−224.913	−244.817	−264.889	−215.827
HLA‐A ^∗^24:02	37	46	10	TYKNNFTLTV	0.7	1.09	No	Yes	−244.793	−230.715	−227.889	−280.76	−205.265

Abbreviations: MHC, major histocompatibility complex; PDB, protein data bank.

MHC‐II binding analysis identified six peptide sequences with superior characteristics: NAKATYEAALKQYEA, KATYEAALKQYEADL, KATYEAALKQYEAD, AKATYEAALKQYEA, ATYEAALKQYEADL, and EDEQTSIKAALAEL. All of these epitopes were included in the final MEV construct.

Experimental validation data retrieved from the IEDB database were obtained for the initial five sequences, all of which exhibited binding energies lower than −200 kcal/mol. The sequence EDEQTSIKAALAEL displayed a particular affinity for murine MHC‐II, as evidenced by its rank score falling <1.0 (Table 2).

**Table 2:** MHC‐II epitopes.

Alleles with percentile rank score <1	Start	End	Length	Peptide	The most favorable percentile rank for alleles	Vaxijen score	Found in experimental data (Yes/No)?	Binding to the MHC‐II in Mice, based on rank scores?	Docking score with human MHC‐II (HLA‐DRB1‐1101) PDB ID, 6CPL	Docking sscore with mice MHC‐II (H2‐IAd) PDB ID, 2IAD
HLA‐DRB1 ^∗^08:01	361	375	15	NAKATYEAALKQYEA	0.19	0.94	Yes	No	−226.714	−235.12
HLA‐DRB1 ^∗^08:01; HLA‐DRB1 ^∗^11:01	363	377	15	KATYEAALKQYEADL	0.04	0.83	Yes	No	−223.646	−226.523
HLA‐DRB1 ^∗^11:01	363	376	14	KATYEAALKQYEAD	0.56	0.82	Yes	No	−207.249	−206.524
HLA‐DRB1 ^∗^08:01	362	375	14	AKATYEAALKQYEA	0.07	0.75	Yes	No	−210.424	−208.976
HLA‐DRB1 ^∗^08:01	364	377	14	ATYEAALKQYEADL	0.3	0.73	Yes	No	−213.387	−222.196
HLA‐DQA1 ^∗^01:02/DQB1 ^∗^06:02	477	490	14	EDEQTSIKAALAEL	0.68	0.86	No	Yes	−215.355	−184.506

Abbreviations: MHC, major histocompatibility complex; PDB, protein data bank.

### 3.2. B‐Cell Epitopes

Six heptapeptide sequences (PTPTPDQ, PPTRTPD, PPTPTPD, PTRTPDQ, TRTPDQA, and PDQAEPN) were optimal for B‐cell activation (Table 3). Experimentally characterized epitope data from the IEDB database were obtained for each sequence, and all peptides exhibited VaxiJen scores exceeding 1.0. The significance of the epitopes was further substantiated by multiple predictive methodologies, including Chou & Fasman Beta‐Turn, Emini Surface Accessibility, Karplus & Schulz Flexibility, Parker Hydrophilicity, and Bepipred Prediction algorithms. Notably, all constituent residues within these epitopes achieved scores surpassing the established thresholds.

**Table 3:** B‐cell epitopes.

Start	End	Epitope	Vaxijen score	Chou and Fasman score for core residue	Emini score for core residue	Karplus and Schulz score for core residue	Parker score for core residue	Bepipred score for core residue	Confirmed by the Ellipro algorithm
949	955	PTPTPDQ	1.78	1.274	2.571	1.066	4.671	0.585	Yes
870	876	PPTRTPD	1.71	1.27	2.907	1.056	4.414	0.569	Yes
948	954	PPTPTPD	1.36	1.351	2.295	1.06	4.114	0.579	Yes
871	877	PTRTPDQ	1.25	1.193	3.256	1.054	4.971	0.569	Yes
872	878	TRTPDQA	1.24	1.07	2.127	1.049	4.971	0.569	Yes
875	881	PDQAEPN	1.11	1.206	2.246	1.044	5.3	0.578	Yes

*Note:* The established threshold values for the respective algorithms were determined as follows: Chou and Fasman Beta‐Turn (1.003), Emini Surface Accessibility (1.0), Karplus and Schulz Flexibility (1.012), Parker Hydrophilicity (2.571), and Bepipred Prediction (0.5).

The identification of multiple BCEs with high prediction scores suggests that the vaccine construct has strong potential to induce a robust humoral immune response, which is essential for neutralizing *S. mutans* adhesion.

### 3.3. Engineering MEV

A novel MEV has been developed by incorporating immunodominant epitopes isolated from the SpaP antigen. The selection process identified six distinct sequences demonstrating superior MHC‐I‐binding properties. Additionally, six epitope sequences were chosen based on their predicted high‐affinity interactions with MHC‐II molecules. The construct was further enhanced by integrating six B‐cell‐activating epitopes selected for their capacity to elicit robust humoral responses. By systematically permuting these components, 100 distinct vaccine candidates were synthesized, each comprising 402 amino acid residues (Supporting Information 5: Data 5). The comprehensive design strategy yielded 100 unique vaccine configurations, achieved through methodical rearrangement of the MHC‐I, MHC‐II, and BCEs. This systematic approach facilitated the exploration of varied structural arrangements while maximizing immunogenic efficacy.

### 3.4. Assessment of the Prime Vaccine Construct: From Allergenicity to Solubility Parameters

**Figure 2:**
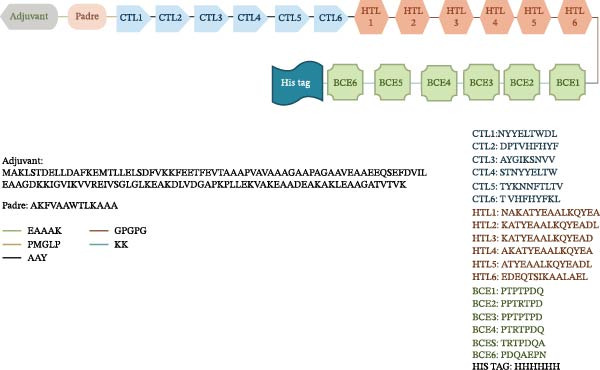
Schematic representation of the multiepitope vaccine’s structural components and their sequential arrangement, highlighting the key elements of the final design. Abbreviations: BCE, B‐cell epitope; CTL, cytotoxic T lymphocyte; HTL, helper T lymphocyte.

A rigorous evaluation framework assessed the portfolio of 100 MEV candidates. Each construct underwent detailed characterization, including assessment of allergenic potential, predicted immunogenicity, physicochemical properties related to solubility, and additional biophysical characteristics (Supporting Information 6: Data 6). The optimal vaccine design was determined through a comprehensive analysis and quantitative assessment of these essential attributes. The selected candidate demonstrated superior properties, including verified nonallergenic status, stability indicated by an instability index of 10.85, structural robustness reflected by an aliphatic index of 67.54, and appropriate acid–base characteristics with a theoretical pI of 5.88. Further favorable characteristics included a predicted mammalian reticulocyte half‐life of 30 h, hydrophilic tendencies indicated by a GRAVY score of −0.498, and strong antigenicity, as indicated by a Vaxijen score of 0.8287. They projected an aqueous solubility likelihood of 98%. The sequential organization of components within the optimized MEV construct is illustrated in Figure 2.

The final vaccine construct demonstrated favorable physicochemical properties—an instability index of 10.85 (high stability), 98% solubility, and a VaxiJen score of 0.8287 (strong antigenicity)—indicating that this MEV is well positioned for experimental production and subsequent evaluation.

### 3.5. Structure Modeling, Refinement, and Evaluation

**Figure 3:**
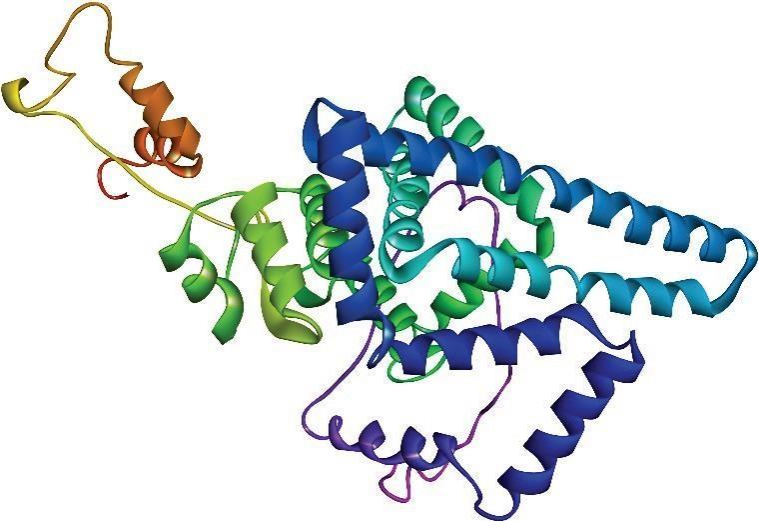
Three‐dimensional structure of the designed multiepitope vaccine, colored in the rainbow spectrum.

**Figure 4:**
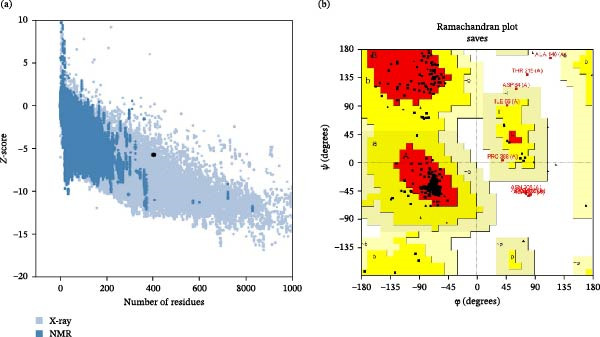
Quality assessment of the refined 3D structure of the vaccine construct. (a) ProSA‐web analysis showing the model’s *Z*‐score of −5.71. (b) Ramachandran plot analysis revealed residue distributions with 88.8% in favored regions, 8.6% in additionally allowed regions, 0.6% in generously allowed regions, and 2.1% in disallowed regions.

The i‐TASSER web server predicted a *C*‐score of −2.54 for the modeled structure. Model quality can be interpreted using the *C*‐score range of −5 to 2, where higher values indicate greater model accuracy. The initial structure was refined using the Galaxy Refine tool, followed by comprehensive validation. The resultant refined structure is illustrated in Figure 3. Analysis conducted via ProSA‐web revealed that the refined model exhibited a *Z*‐score of −5.71, which demonstrates consistency with naturally occurring protein conformations (Figure 4a). Further structural assessment through the PROCHECK server’s Ramachandran plot analysis indicated that 88.8% of the amino acid residues were positioned within most favored regions. In comparison, 8.6%, 0.6%, and 2.1% of residues were distributed across additionally allowed, generously allowed, and disallowed regions, respectively (Figure 4b).

### 3.6. Binding Affinity Assessment Between MEV and TLR4

**Figure 5:**
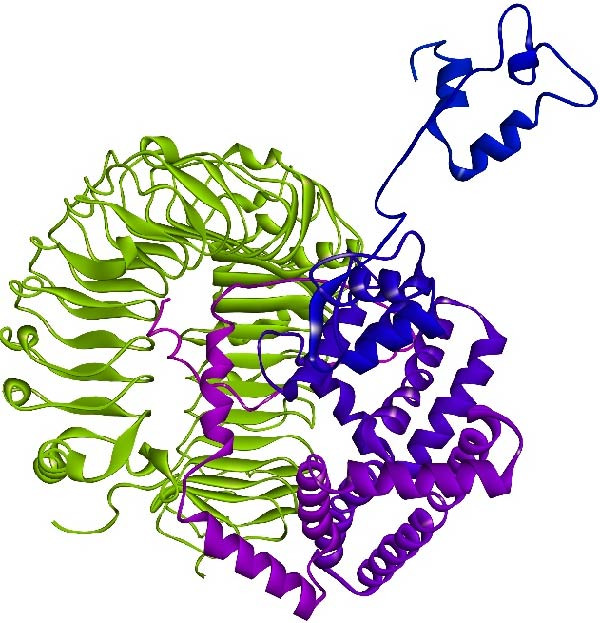
Molecular visualization of the docked complex showing the vaccine construct (blue and violet) bound to the TLR4 receptor (light green).

Among 100 distinct conformational poses, the optimal interaction complex exhibited a binding energy of −315.77 kcal/mol and a 90% confidence score (Figure 5).

### 3.7. Stability of MEV‐TLR4

**Figure 6:**
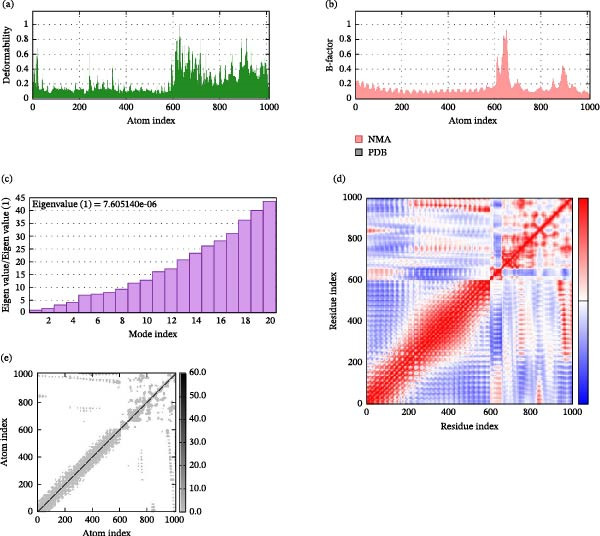
The NMA of the vaccine‐TLR4 complex using the iMODS server. (a) Graph showing structural deformability patterns. (b) B‐factor analysis plot indicating atomic fluctuations. (c) Eigenvalue plot demonstrating the complex’s internal motion characteristics. (d) The covariance matrix displays residue motion correlations: red for coordinated movements, white for uncorrelated, and blue for opposite movements. (e) In the elastic network model, darker gray indicates stronger inter‐residue connections. Abbreviations: NMA, normal mode analysis; PDB, protein data bank; TLR4, Toll‐like receptor 4.

The NMA results revealed that the vaccine and TLR4 bind very stably. Several findings supported this stability. The structure showed very few points where it could easily bend or flex (Figure 6a). The B‐factor analysis showed minimal movement within the vaccine‐TLR4 complex, indicating a strong and rigid structure (Figure 6b). A low eigenvalue (7.61 × 10^−6^) confirmed the rigidity of the complex, suggesting thatit would take a lot of energy to change its shape at the binding interface (Figure 6c). A covariance matrix showed how the different parts of the complex interact. Red areas indicated parts that move together, white areas showed no correlation, and blue areas showed parts that move in opposite directions (Figure 6d). Elastic network modeling highlighted varying degrees of flexibility across the structure. Darker gray areas indicated regions of higher rigidity (Figure 6e).

### 3.8. Reverse Transcription and Codon Optimization

**Figure 7:**
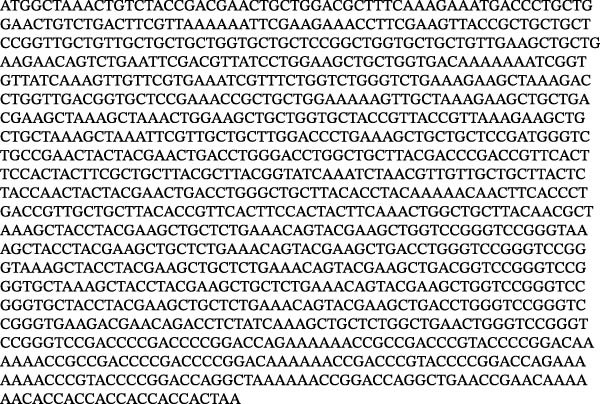
DNA sequence optimization results showing the final nucleotide sequence designed to express the multiepitope vaccine construct.

The resultant analysis revealed optimal codon usage, as evidenced by a Codon Adaptation Index (CAI) of 1.0 for the *E. coli* K12 strain and a GC composition of 53.25% (Figure 7).

### 3.9. In Silico Cloning

**Figure 8:**
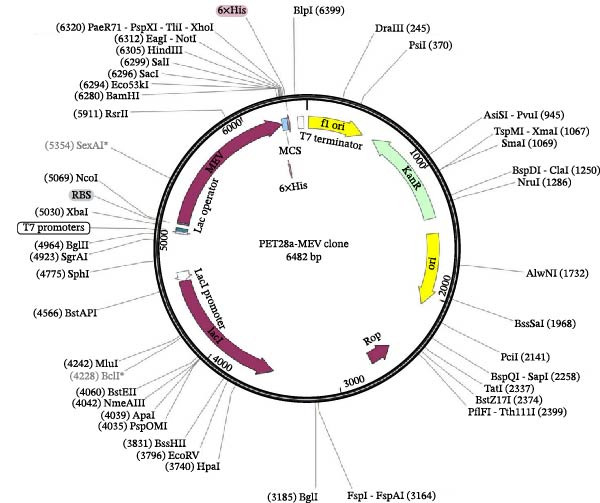
In silico cloning of the optimized MEV gene into the *pET‑28a*(+) vector using NcoI and XhoI sites, generating the recombinant pET28a‑MEV construct. The plasmid map shows key vector elements, including the T7 promoter, lac operator, His‑tag, KanR marker, and the correctly inserted MEV sequence.

The pET28a‐MEV recombinant vector map is shown in Figure 8. The MEV gene (purple) is designed for high‐level expression in *E. coli* within the pET28a(+) backbone, regulated by a *lac* operator. The 6482 bp construct includes a C‐terminal 6×His tag for purification and a *KanR* gene for selection.

## 4. Discussion

Scientists have been researching vaccines to prevent and treat tooth decay [55, 56]. Vaccination offers a promising approach to long‐lasting protection against this common disease [57]. Animal studies using active immunization have yielded encouraging results, demonstrating that various administration routes (subcutaneous, oral, nasal, and direct) can induce protective antibodies, including IgA in saliva and IgG in blood. For instance, a vaccine composed of specific bacterial proteins (cell surface adhesion proteins or Antigen I/II) reduced tooth decay by 70% in monkeys when administered subcutaneously [58]. Recent advancements in reverse vaccinology and immunoinformatics have spurred interest in MEVs [59, 60]. MEVs offer several advantages over traditional vaccines: enhanced immunogenicity, reduced allergenicity, cost‐effectiveness, faster development time, and fewer side effects. Critically, MEVs can simultaneously stimulate multiple immune responses, including humoral, innate, and cellular responses, providing broader protection than single‐component vaccines [61]. Immunoinformatics‐designed MEVs have demonstrated protective effects in vivo; many are currently in clinical trials [62]. While MEVs have shown promise against various pathogens, including SARS‐CoV‐2, *M. tuberculosis*, *Serratia marcescens*, *Staphylococcus aureus*, Group B *Streptococcus*, and Monkeypox virus [63–68], research on their application against *S. mutans* remains limited.

Recent advances in oral vaccine development have underscored the benefits of needle‐free immunization, such as enhanced patient compliance, ease of administration, and the capacity to elicit immune responses at mucosal surfaces. Oral vaccine platforms are also being actively investigated to address challenges related to antigen degradation within the gastrointestinal tract and to improve delivery efficiency through optimized carriers and formulations [69, 70]. These attributes make oral vaccination particularly attractive for pathogens that establish infection at mucosal entry sites. Mucosal immunity can trigger immune responses both locally and at distant mucosal sites while also generating systemic reactions that help prevent further invasion by primary pathogens. Among the various effectors, IgA occupies a central role in mucosal immunity [71, 72]. The present study aimed to design an MEV targeting *S. mutans* to prevent dental caries. Given its inherent advantages, oral delivery is considered the preferred route of administration for this vaccine candidate. However, this route must be validated in appropriate animal models and subsequently in clinical trials.

Recently, Chao et al. [73] analyzed the core proteome of several bacterial species, including *S. mutans*, to identify conserved epitopes for a universal vaccine candidate. While that ambitious approach seeks broad protection against multiple systemic pathogens, the present study employed a more targeted strategy focused specifically on dental caries prevention. Rather than screening a pan‐proteome, this study concentrated on a single, critical virulence factor—the SpaP antigen of *S. mutans*. This focused approach enables the design of a highly specialized construct intended to elicit a precise mucosal immune response against the primary etiological agent of dental caries, representing a tailored strategy for a localized, nonsystemic disease. Both strategies underscore the power of reverse vaccinology. Yet, they diverge in scope, highlighting the adaptability of MEV design for both universal, multipathogen targets and specific, single‐antigen applications such as the present study.

Naorem et al. [74] designed an MEV using computational tools, focusing on five potential vaccine target proteins (PBP2X, PBP2b, MurG, ATP‐F, and AGPAT). The authors employed several in silico tools (CELLO v.2.5, Vaxign v2.0, ANTIGENpro, and AllerTop v2.0) to assess the efficacy and safety of these proteins as vaccine candidates. Subsequently, SVMTrip and NetCTL/NetMHC II were used to identify specific immune‐stimulating epitopes within these proteins. The resulting MEV construct comprised 10 cytotoxic CTL epitopes, 5 HTL epitopes, and 5 linear BCEs, all linked by linkers. Computational analysis of the designed MEV predicted favorable vaccine characteristics, including high antigenicity, good solubility, stability, and the ability to induce both humoral and cellular immune responses. Docking studies with TLRs and MHC molecules showed promising interactions, particularly a strong, stable binding affinity with TLR‐4, characterized by low energy scores and low dissociation probability. Further 40‐nanosecond MD simulations of the MEV‐TLR‐4 complex confirmed its stability, demonstrating minimal structural fluctuations.

De Souza Pereira et al. [75] expressed a modified version of the *S. mutans* GlnH protein (rGlnH) in *Bacillus subtilis* and evaluated its potential as a vaccine in mice. The mice received sublingual vaccinations with rGlnH formulated with the mucosal adjuvant LTK63, a detoxified *E. coli* heat‐labile enterotoxin derivative. The study assessed the induction of anti‐rGlnH antibodies and their protective efficacy. The results demonstrated that sublingual rGlnH vaccination induced a robust antibody response against the target protein. Furthermore, these antibodies passively protected unvaccinated mice from *S. mutans* adherence. Vaccinated mice also exhibited partial protection against oral colonization by the *S. mutans* NG8 strain. The study by De Souza Pereira et al. [75] reported that rGlnH was a promising vaccine candidate, eliciting protective antibodies and conferring partial protection against *S. mutans* colonization. Their study also supported the feasibility of developing a mucosal vaccine against dental caries.

This research aimed to develop an MEV against *S. mutans*, the bacteria responsible for tooth decay. The vaccine was designed using computational methods, focusing on the *S. mutans* SpaP protein. Promising SpaP epitopes were identified as capable of stimulating CTLs, HTLs, and B cells in both human and mouse models. These cell types play crucial roles in both immediate and long‐term immune responses. Two adjuvants, a TLR4 agonist sequence and the PADRE peptide, were incorporated to enhance the vaccine’s immunogenicity. The resulting MEV, comprising the selected epitopes and adjuvants, was then subjected to in silico analysis to assess its binding affinity for the TLR4 immune receptor.

Various linkers were employed to construct the vaccine. The “EAAAK” linker provided stability and maintained appropriate spacing between the TLR4 agonist and PADRE. The TLR4 agonist was connected to the first CTL epitope using a cathepsin S cleavable linker (“PMGLP”) [43]. “AAY” linkers facilitated the accessibility and presentation of CTL epitopes, thereby enhancing their recognition by the immune system. The “GPGPG” linker joined the HTL epitopes, promoting a proper HTL immune response [76, 77]. Finally, a flexible “KK” linker connected the BCEs [78, 79].

Computational analysis of the novel vaccine design revealed favorable properties. The vaccine exhibited a low instability index of 10.85, predicting high stability. Notably, the study also indicated a lack of allergenic potential. A negative GRAVY score of −0.498 suggests good water solubility and hydrophilic character of MEV. The predicted solubility in *E. coli* was high (98%), a crucial factor for production. Furthermore, a Vaxijen score of 0.8277 suggests strong potential to elicit an immune response. These characteristics indicate that this MEV design holds considerable promise for further development.

The docking analysis predicted an extremely strong binding affinity between the vaccine candidate and TLR4, with a Δ*G*_binding_ value of −315.77 kcal/mol. This suggests that the vaccine holds significant promise as a TLR4 agonist, potentially stimulating the immune system.

A relatively high eigenvalue of 7.61 × 10^−6^ was obtained through NMA, by which deformation in the binding interface between the MEV construct and TLR4 was shown to be induced by substantial energy. Therefore, the integrity and functionality of the MEV‐TLR4 complex were suggested to be maintained by a high degree of structural stability and rigidity, which is desirable for the vaccine’s performance.

The computational results obtained in this study should be interpreted in the context of their potential biological relevance. Dental caries prevention requires a vaccine strategy that induces salivary secretory IgA (sIgA) at the oral mucosa [80, 81]. For this reason, the construct is best suited for intranasal, rather than routine intramuscular, administration [80, 80]. The TLR4 agonist included in our design is not proposed solely as a systemic adjuvant; when delivered via mucosal routes, TLR4 agonists can promote both mucosal and systemic immune responses [82, 83]. PADRE is incorporated to provide broad T‐helper support and enhance downstream humoral responses, including those relevant to mucosal immunity [84]. In addition, antibody responses targeting SpaP may reduce *S. mutans*’s ability to bind salivary agglutinin receptors, thereby limiting plaque formation and the subsequent development of dental caries. As the current study is in silico, experimental confirmation of salivary IgA induction will be required in future mucosal vaccination studies.

A CAI of 1.0 for the vaccine’s DNA sequence suggests a perfect match with *E. coli* K12’s preferred codons, predicting efficient protein production in this bacterium. While *E. coli* typically exhibits a GC content of 50.4%−50.8%, the MEV gene’s GC content of 53.25% is reasonably close to this range, suggesting that the vaccine’s mRNA will maintain good stability and function within host cells [26].

Among all computational predictions, four findings were identified as most critical for the vaccine. Strong antigenicity (VaxiJen score: 0.8287) combined with a nonallergenic status was demonstrated by the final MEV construct, indicating its potential to induce a protective immune response without adverse reactions. In addition, efficient activation of innate immunity—a prerequisite for adaptive immune responses—is suggested by the exceptionally strong binding affinity (Δ*G* = −315.77 kcal/mol) observed between MEV and TLR4. Moreover, the feasibility of experimental production is supported by the high predicted solubility (98%) and low instability index (10.85). Additionally, the vaccine’s manufacturability is confirmed by a CAI of 1.0 for *E. coli* K12. Together, the proposed MEV is predicted to be a strong candidate for experimental validation based on these four features.

The strength of this study lies in its comprehensive immunoinformatics workflow, which integrates multiple prediction tools to identify highly antigenic and nontoxic epitopes, thereby enabling the rational design of a targeted MEV candidate against *S. mutans*.

Several limitations and challenges in translating the findings into experimental validation had to be recognized, even though this computational design demonstrated considerable potential for the SpaP‐targeting vaccine. Various obstacles were encountered during the transition from in silico predictions to in vitro and in vivo experiments, whereby computational models could not fully capture biological response variations. The predicted highly antigenic T‐cell and BCEs were found to be potentially limited in their ability to encompass all individual variations in immune responses, due to genetic diversity across populations, suggesting a need to evaluate multiple epitopes and their combinations in future research. Besides, while the present study provides a comprehensive in silico evaluation of the immunogenic and structural characteristics of the MEV construct, the impact of vaccination on *S. mutans* biofilm formation, plaque integration, and the overall ecological composition of dental plaque lies beyond the scope of computational analysis. These parameters can only be reliably assessed through laboratory‐based and in vivo studies that investigate bacterial adhesion, biofilm maturation, and postimmunization shifts in oral microbial communities. Consequently, future experimental investigations should examine whether MEV reduces *S. mutans* colonization within multispecies biofilms and whether it confers broader protective benefits against dental caries and periodontal disease. Ultimately, we aim to advance this designed MEV construct toward the development of a successful next‐generation vaccine candidate for the prevention of dental caries.

## 5. Conclusions

This study describes the rational design of an MEV targeting *S. mutans* SpaP using an integrated immunoinformatics workflow. Combining epitope prediction, structural modeling, and docking analysis, the work highlights SpaP as a promising target for preventing early adhesion and colonization in dental caries. These computational findings provide a mechanistic framework suggesting that a SpaP‐focused multiepitope construct may stimulate protective immune responses against *S. mutans*. Future experimental validation should include in vitro expression, structural characterization, immunogenicity assays, and in vivo studies of mucosal antibody responses and efficacy—essential steps for translating this vaccine candidate into clinical application.

## Author Contributions

**Amir Taherkhani**: conceptualization, data curation, methodology, supervision, investigation, writing – original draft, writing – review and editing. **Ebrahim Yarmohammadi**: conceptualization, data curation, supervision, investigation, and editing. **Fazilat Khademi**: data curation, methodology, formal analysis, and investigation.

## Acknowledgments

The authors acknowledge the support of the Deputy of Research and Technology, the Research Center for Molecular Medicine, and the Dental Research Center, Hamadan University of Medical Sciences, Hamadan, Iran. The manuscript was first edited using Generative AI to improve writing style, then underwent grammatical editing with Grammarly Premium.

## Funding

This study did not receive any specific funding.

## Disclosure

All authors have read and approved the final version of the manuscript. Amir Taherkhani had full access to all data in this study and takes full responsibility for the integrity and accuracy of the data analysis.

## Ethics Statement

The present study has been confirmed by the Ethics Committee of Hamadan University of Medical Sciences, Hamadan, Iran (IR.UMSHA.REC.1402.218).

## Conflicts of Interest

The authors declare no conflicts of interest.

## Supporting Information

Additional supporting information can be found online in the Supporting Information section.

## Supporting information


**Supporting Information 1** Data 1: SpaP sequence.

**Supporting Information 2** Data 2: Experimentally validated epitopes in SpaP.

**Supporting Information 3** Data 3: MHC‐I epitopes with percentile rank scores <1.

**Supporting Information 4** Data 4: MHC‐II epitopes with percentile rank scores <1.

**Supporting Information 5** Data 5: A total of 100 possible multiepitope vaccine sequences.

**Supporting Information 6** Data 6: Multiepitope vaccine sequences evaluation.

## Data Availability

The authors confirm that the data supporting the findings of this study are available within the article and/or its Supporting Information.
