# Supplementary material for: Design of a Novel Peptide‐Based Vaccine Targeting Streptococcus mutans SpaP Antigen for Dental Caries Prevention
Source: Int J Dent. 2026 Jun 29;2026:5545020. doi: 10.1155/ijod/5545020 (PMC13312148; doi:10.1155/ijod/5545020)
Supplement: Supplementary file 1 — Supporting Information 1 Data 1: SpaP sequence. [file IJOD-2026-5545020-s001.docx]

>sp|P11657|PAC_STRMG Major cell-surface adhesin PAc OS=Streptococcus mutans OX=1309 GN=pac PE=1 SV=1

MKVKKTYGFRKSKISKTLCGAVLGTVAAVSVAGQKVFADETTTTSDVDTKVVGTQTGNPA

TNLPEAQGSASKEAEQSQTKLERQMVHTIEVPKTDLDQAAKDAKSAGVNVVQDADVNKGT

VKTPEEAVQKETEIKEDYTKQAEDIKKTTDQYKSDVAAHEAEVAKIKAKNQATKEQYEKD

MAAHKAEVERINAANAASKTAYEAKLAQYQADLAAVQKTNAANQAAYQKALAAYQAELKR

VQEANAAAKAAYDTAVAANNAKNTEIAAANEEIRKRNATAKAEYETKLAQYQAELKRVQE

ANAANEADYQAKLTAYQTELARVQKANADAKATYEAAVAANNAKNAALTAENTAIKQRNE

NAKATYEAALKQYEADLAAVKKANAANEADYQAKLTAYQTELARVQKANADAKAAYEAAV

AANNAANAALTAENTAIKKRNADAKADYEAKLAKYQADLAKYQKDLADYPVKLKAYEDEQ

TSIKAALAELEKHKNEDGNLTEPSAQNLVYDLEPNANLSLTTDGKFLKASAVDDAFSKST

SKAKYDQKILQLDDLDITNLEQSNDVASSMELYGNFGDKAGWSTTVSNNSQVKWGSVLLE

RGQSATATYTNLQNSYYNGKKISKIVYKYTVDPKSKFQGQKVWLGIFTDPTLGVFASAYT

GQVEKNTSIFIKNEFTFYHEDEKPINFDNALLSVTSLNREHNSIEMAKDYSGKFVKISGS

SIGEKNGMIYATDTLNFKQGEGGSRWTMYKNSQAGSGWDSSDAPNSWYGAGAIKMSGPNN

HVTVGATSATNVMPVSDMPVVPGKDNTDGKKPNIWYSLNGKIRAVNVPKVTKEKPTPPVK

PTAPTKPTYETEKPLKPAPVAPNYEKEPTPPTRTPDQAEPNKPTPPTYETEKPLEPAPVE

PSYEAEPTPPTRTPDQAEPNKPTPPTYETEKPLEPAPVEPSYEAEPTPPTPTPDQPEPNK

PVEPTYEVIPTPPTDPVYQDLPTPPSDPTVHFHYFKLAVQPQVNKEIRNNNDINIDRTLV

AKQSVVKFQLKTADLPAGRDETTSFVLVDPLPSGYQFNPEATKAASPGFDVTYDNATNTV

TFKATAATLATFNADLTKSVATIYPTVVGQVLNDGATYKNNFTLTVNDAYGIKSNVVRVT

TPGKPNDPDNPNNNYIKPTKVNKNENGVVIDGKTVLAGSTNYYELTWDLDQYKNDRSSAD

TIQKGFYYVDDYPEEALELRQDLVKITDANGNEVTGVSVDNYTNLEAAPQEIRDVLSKAG

IRPKGAFQIFRADNPREFYDTYVKTGIDLKIVSPMVVKKQMGQTGGSYENQAYQIDFGNG

YASNIVINNVPKINPKKDVTLTLDPADTNNVDGQTIPLNTVFNYRLIGGIIPANHSEELF

EYNFYDDYDQTGDHYTGQYKVFAKVDITLKNGVIIKSGTELTQYTTAEVDTTKGAITIKF

KEAFLRSVSIDSAFQAESYIQMKRIAVGTFENTYINTVNGVTYSSNTVKTTTPEDPADPT

DPQDPSSPRTSTVIIYKPQSTAYQPSSVQETLPNTGVTNNAYMPLLGIIGLVTSFSLLGL

KAKKD
