# Supplementary material for: Design of a Novel Peptide‐Based Vaccine Targeting Streptococcus mutans SpaP Antigen for Dental Caries Prevention
Source: Int J Dent. 2026 Jun 29;2026:5545020. doi: 10.1155/ijod/5545020 (PMC13312148; doi:10.1155/ijod/5545020)
Supplement: Supplementary file 2 — Supporting Information 2 Data 2: Experimentally validated epitopes in SpaP. [file IJOD-2026-5545020-s002.docx]

| Epitope | Epitope | Epitope |
| --- | --- | --- |
| Name | Starting Position | Ending Position |
| NAKATYEAALKQYEADLAAVKKANAA | 361 | 386 |
| AANNAKNAAL | 339 | 348 |
| ADTIQKGFYY | 1199 | 1208 |
| AGIRPKGAFQ | 1259 | 1268 |
| ANEEIRKRNA | 269 | 278 |
| APTKPTYETE | 843 | 852 |
| AQYQAELKRV | 289 | 298 |
| AYGIKSNVVR | 1129 | 1138 |
| GKKPNIWYSL | 809 | 818 |
| KFKEAFLRSV | 1439 | 1448 |
| KQMGQTGGSY | 1299 | 1308 |
| KVTKEKPTPP | 829 | 838 |
| LKIVSPMVVK | 1289 | 1298 |
| LKNGVIIKSG | 1409 | 1418 |
| NVPKINPKKD | 1329 | 1338 |
| PQEIRDVLSK | 1249 | 1258 |
| PTAPTKPTYE | 841 | 850 |
| QTELARVQKA | 317 | 326 |
| SLLGLKAKKD | 1556 | 1565 |
| TAPTKPTYET | 842 | 851 |
| TVTFKATAAT | 1079 | 1088 |
| VKPTAPTKPT | 839 | 848 |
| VQPQVNKEIR | 999 | 1008 |
| YIQMKRIAVG | 1459 | 1468 |
