# Supplementary material for: Design of a Novel Peptide‐Based Vaccine Targeting Streptococcus mutans SpaP Antigen for Dental Caries Prevention
Source: Int J Dent. 2026 Jun 29;2026:5545020. doi: 10.1155/ijod/5545020 (PMC13312148; doi:10.1155/ijod/5545020)
Supplement: Supplementary file 3 — Supporting Information 3 Data 3: MHC‐I epitopes with percentile rank scores <1. [file IJOD-2026-5545020-s003.docx]

| **allele** | **start** | **end** | **length** | **peptide** | **percentile_rank** |
| --- | --- | --- | --- | --- | --- |
| HLA-B*40:01 | 4 | 13 | 10 | KEFTVSGNIL | 0.06 |
| HLA-A*68:01 | 7 | 16 | 10 | TVSGNILTIR | 0.06 |
| HLA-B*07:02 | 3 | 12 | 10 | GPHGGAASGL | 0.07 |
| HLA-B*18:01 | 28 | 36 | 9 | LEFYLAMPF | 0.08 |
| HLA-B*38:01 | 21 | 29 | 9 | DHRQLQLSI | 0.10 |
| HLA-B*35:01 | 34 | 42 | 9 | MPFATPMEA | 0.12 |
| HLA-B*51:01 | 34 | 42 | 9 | MPFATPMEA | 0.12 |
| HLA-B*07:02 | 22 | 30 | 9 | GPESRLLEF | 0.13 |
| HLA-A*02:01 | 48 | 56 | 9 | SLAQDAPPL | 0.14 |
| HLA-B*35:01 | 32 | 40 | 9 | LAMPFATPM | 0.14 |
| HLA-B*40:01 | 40 | 49 | 10 | MEAELARRSL | 0.16 |
| HLA-A*02:01 | 26 | 34 | 9 | RLLEFYLAM | 0.21 |
| HLA-B*07:02 | 44 | 53 | 10 | GPRGAGAARA | 0.21 |
| HLA-B*07:02 | 21 | 30 | 10 | RGPESRLLEF | 0.21 |
| HLA-B*35:01 | 22 | 30 | 9 | GPESRLLEF | 0.22 |
| HLA-B*40:01 | 4 | 12 | 9 | KEFTVSGNI | 0.24 |
| HLA-B*52:01 | 4 | 12 | 9 | KEFTVSGNI | 0.27 |
| HLA-A*68:01 | 51 | 59 | 9 | LAQPPSGQR | 0.28 |
| HLA-B*58:01 | 33 | 41 | 9 | LQQLSLLMW | 0.28 |
| HLA-B*27:05 | 20 | 28 | 9 | ARGPESRLL | 0.28 |
| HLA-B*44:02 | 40 | 49 | 10 | MEAELARRSL | 0.28 |
| HLA-B*35:01 | 36 | 44 | 9 | FATPMEAEL | 0.29 |
| HLA-B*08:01 | 19 | 27 | 9 | AADHRQLQL | 0.29 |
| HLA-B*52:01 | 34 | 42 | 9 | QQLSLLMWI | 0.29 |
| HLA-B*51:01 | 36 | 44 | 9 | FATPMEAEL | 0.31 |
| HLA-B*15:02 | 32 | 40 | 9 | LAMPFATPM | 0.32 |
| HLA-A*68:01 | 50 | 59 | 10 | FLAQPPSGQR | 0.35 |
| HLA-B*52:01 | 7 | 15 | 9 | TVSGNILTI | 0.37 |
| HLA-B*27:05 | 25 | 34 | 10 | SRLLEFYLAM | 0.39 |
| HLA-A*11:01 | 37 | 46 | 10 | ATPMEAELAR | 0.39 |
| HLA-B*51:01 | 32 | 40 | 9 | LAMPFATPM | 0.40 |
| HLA-B*52:01 | 25 | 33 | 9 | LQLSISSCL | 0.41 |
| HLA-A*31:01 | 8 | 16 | 9 | VSGNILTIR | 0.43 |
| HLA-A*02:01 | 50 | 58 | 9 | AQDAPPLPV | 0.43 |
| HLA-B*07:02 | 34 | 42 | 9 | MPFATPMEA | 0.44 |
| HLA-B*07:02 | 19 | 27 | 9 | GARGPESRL | 0.47 |
| HLA-B*44:02 | 22 | 31 | 10 | GPESRLLEFY | 0.48 |
| HLA-A*01:01 | 19 | 27 | 9 | AADHRQLQL | 0.49 |
| HLA-A*31:01 | 51 | 59 | 9 | LAQPPSGQR | 0.50 |
| HLA-B*38:01 | 43 | 51 | 9 | TQCFLPVFL | 0.50 |
| HLA-B*44:02 | 23 | 31 | 9 | PESRLLEFY | 0.50 |
| HLA-A*26:01 | 7 | 15 | 9 | TVSGNILTI | 0.53 |
| HLA-B*08:01 | 22 | 30 | 9 | GPESRLLEF | 0.55 |
| HLA-A*68:01 | 37 | 46 | 10 | ATPMEAELAR | 0.56 |
| HLA-B*08:01 | 12 | 20 | 9 | ILTIRLTAA | 0.56 |
| HLA-B*18:01 | 23 | 31 | 9 | PESRLLEFY | 0.56 |
| HLA-B*15:02 | 38 | 46 | 9 | LLMWITQCF | 0.57 |
| HLA-B*52:01 | 43 | 51 | 9 | TQCFLPVFL | 0.57 |
| HLA-B*38:01 | 20 | 28 | 9 | ARGPESRLL | 0.58 |
| HLA-A*11:01 | 7 | 16 | 10 | TVSGNILTIR | 0.59 |
| HLA-B*38:01 | 4 | 12 | 9 | PHGGAASGL | 0.59 |
| HLA-B*08:01 | 41 | 49 | 9 | EAELARRSL | 0.60 |
| HLA-A*02:01 | 37 | 45 | 9 | SLLMWITQC | 0.63 |
| HLA-B*18:01 | 40 | 49 | 10 | MEAELARRSL | 0.63 |
| HLA-A*02:01 | 28 | 36 | 9 | SISSCLQQL | 0.64 |
| HLA-B*08:01 | 21 | 29 | 9 | DHRQLQLSI | 0.65 |
| HLA-B*44:02 | 28 | 36 | 9 | LEFYLAMPF | 0.65 |
| HLA-B*07:02 | 19 | 27 | 9 | AADHRQLQL | 0.66 |
| HLA-B*44:02 | 4 | 12 | 9 | KEFTVSGNI | 0.66 |
| HLA-B*07:02 | 41 | 49 | 9 | EAELARRSL | 0.67 |
| HLA-B*52:01 | 21 | 29 | 9 | DHRQLQLSI | 0.67 |
| HLA-A*31:01 | 7 | 16 | 10 | TVSGNILTIR | 0.69 |
| HLA-B*27:05 | 25 | 33 | 9 | SRLLEFYLA | 0.69 |
| HLA-B*40:01 | 28 | 36 | 9 | LEFYLAMPF | 0.69 |
| HLA-B*38:01 | 50 | 58 | 9 | AQDAPPLPV | 0.69 |
| HLA-B*58:01 | 17 | 25 | 9 | LTAADHRQL | 0.71 |
| HLA-B*52:01 | 32 | 40 | 9 | LAMPFATPM | 0.74 |
| HLA-A*31:01 | 52 | 60 | 9 | AQPPSGQRR | 0.75 |
| HLA-B*52:01 | 50 | 58 | 9 | AQDAPPLPV | 0.75 |
| HLA-B*52:01 | 19 | 27 | 9 | AADHRQLQL | 0.75 |
| HLA-B*07:02 | 23 | 31 | 9 | IPDGPGGNA | 0.76 |
| HLA-B*51:01 | 7 | 15 | 9 | TVSGNILTI | 0.77 |
| HLA-B*58:01 | 42 | 50 | 9 | ITQCFLPVF | 0.78 |
| HLA-A*68:01 | 51 | 60 | 10 | LAQPPSGQRR | 0.81 |
| HLA-A*02:01 | 7 | 15 | 9 | TVSGNILTI | 0.81 |
| HLA-A*01:01 | 22 | 31 | 10 | GPESRLLEFY | 0.81 |
| HLA-B*35:01 | 22 | 31 | 10 | GPESRLLEFY | 0.82 |
| HLA-B*52:01 | 33 | 41 | 9 | LQQLSLLMW | 0.88 |
| HLA-B*52:01 | 36 | 44 | 9 | FATPMEAEL | 0.89 |
| HLA-B*27:05 | 15 | 23 | 9 | IRLTAADHR | 0.92 |
| HLA-B*40:01 | 50 | 58 | 9 | AQDAPPLPV | 0.93 |
| HLA-B*51:01 | 14 | 23 | 10 | DADGPGGPGI | 0.94 |
| HLA-B*38:01 | 25 | 33 | 9 | LQLSISSCL | 0.95 |
| HLA-B*07:02 | 44 | 52 | 9 | GPRGAGAAR | 0.96 |
| HLA-B*44:02 | 4 | 13 | 10 | KEFTVSGNIL | 0.96 |
| HLA-B*44:02 | 42 | 50 | 9 | AELARRSLA | 0.96 |
| HLA-B*35:01 | 38 | 46 | 9 | TPMEAELAR | 0.97 |
