# Supplementary material for: Design of a Novel Peptide‐Based Vaccine Targeting Streptococcus mutans SpaP Antigen for Dental Caries Prevention
Source: Int J Dent. 2026 Jun 29;2026:5545020. doi: 10.1155/ijod/5545020 (PMC13312148; doi:10.1155/ijod/5545020)
Supplement: Supplementary file 4 — Supporting Information 4 Data 4: MHC‐II epitopes with percentile rank scores <1. [file IJOD-2026-5545020-s004.docx]

| **allele** | **start** | **end** | **peptide** | **rank** |
| --- | --- | --- | --- | --- |
| **HLA-DRB1*04:01** | 1010 | 1024 | NNDINIDRTLVAKQS | 0.01 |
| **HLA-DRB1*04:01** | 962 | 976 | VEPTYEVIPTPPTDP | 0.01 |
| **HLA-DRB1*04:01** | 963 | 977 | EPTYEVIPTPPTDPV | 0.01 |
| **HLA-DRB1*01:01** | 711 | 724 | SGKFVKISGSSIGE | 0.01 |
| **HLA-DRB1*01:01** | 711 | 725 | SGKFVKISGSSIGEK | 0.01 |
| **HLA-DRB1*08:01** | 362 | 376 | AKATYEAALKQYEAD | 0.02 |
| **HLA-DRB1*04:01** | 900 | 914 | EPSYEAEPTPPTRTP | 0.02 |
| **HLA-DRB1*04:01** | 1009 | 1023 | NNNDINIDRTLVAKQ | 0.02 |
| **HLA-DRB1*04:01** | 963 | 976 | EPTYEVIPTPPTDP | 0.02 |
| **HLA-DRB1*04:01** | 1010 | 1023 | NNDINIDRTLVAKQ | 0.02 |
| **HLA-DRB1*11:01** | 374 | 388 | EADLAAVKKANAANE | 0.02 |
| **HLA-DPA1*01:03/DPB1*04:02** | **1451** | **1464** | **DSAFQAESYIQMKR** | **0.02** |
| **HLA-DRB1*15:01** | 310 | 324 | QAKLTAYQTELARVQ | 0.03 |
| **HLA-DRB1*15:01** | 392 | 406 | QAKLTAYQTELARVQ | 0.03 |
| **HLA-DRB1*04:01** | 1011 | 1024 | NDINIDRTLVAKQS | 0.03 |
| **HLA-DRB1*04:01** | 1513 | 1526 | VIIYKPQSTAYQPS | 0.03 |
| **HLA-DRB1*04:01** | 939 | 953 | EPSYEAEPTPPTPTP | 0.03 |
| **HLA-DRB1*03:01** | 1165 | 1179 | ENGVVIDGKTVLAGS | 0.03 |
| **HLA-DPA1*02:01/DPB1*05:01** | 1451 | 1464 | DSAFQAESYIQMKR | 0.03 |
| **HLA-DPA1*01:03/DPB1*04:02** | 1451 | 1465 | DSAFQAESYIQMKRI | 0.03 |
| **HLA-DPA1*01:03/DPB1*04:02** | 1450 | 1464 | IDSAFQAESYIQMKR | 0.03 |
| **HLA-DRB1*01:01** | 710 | 724 | YSGKFVKISGSSIGE | 0.03 |
| **HLA-DRB1*01:01** | 712 | 725 | GKFVKISGSSIGEK | 0.03 |
| **HLA-DRB1*16:02** | 711 | 725 | SGKFVKISGSSIGEK | 0.04 |
| **HLA-DRB1*15:01** | 309 | 322 | YQAKLTAYQTELAR | 0.04 |
| **HLA-DRB1*15:01** | 391 | 404 | YQAKLTAYQTELAR | 0.04 |
| **HLA-DRB1*15:01** | 1236 | 1250 | GVSVDNYTNLEAAPQ | 0.04 |
| **HLA-DRB1*15:01** | 310 | 323 | QAKLTAYQTELARV | 0.04 |
| **HLA-DRB1*15:01** | 392 | 405 | QAKLTAYQTELARV | 0.04 |
| **HLA-DRB1*08:01** | 363 | 377 | KATYEAALKQYEADL | 0.04 |
| **HLA-DRB1*04:01** | 624 | 637 | KIVYKYTVDPKSKF | 0.04 |
| **HLA-DRB1*04:01** | 1513 | 1527 | VIIYKPQSTAYQPSS | 0.04 |
| **HLA-DRB1*04:01** | 624 | 638 | KIVYKYTVDPKSKFQ | 0.04 |
| **HLA-DRB1*03:01** | 1164 | 1178 | NENGVVIDGKTVLAG | 0.04 |
| **HLA-DRB1*03:01** | 1165 | 1178 | ENGVVIDGKTVLAG | 0.04 |
| **HLA-DRB1*16:02** | 711 | 724 | SGKFVKISGSSIGE | 0.05 |
| **HLA-DRB1*04:01** | 1009 | 1022 | NNNDINIDRTLVAK | 0.05 |
| **HLA-DRB1*04:01** | 900 | 913 | EPSYEAEPTPPTRT | 0.05 |
| **HLA-DPA1*01:03/DPB1*02:01** | 987 | 1000 | DPTVHFHYFKLAVQ | 0.05 |
| **HLA-DPA1*01:03/DPB1*02:01** | 987 | 1001 | DPTVHFHYFKLAVQP | 0.05 |
| **HLA-DRB1*16:02** | 963 | 976 | EPTYEVIPTPPTDP | 0.06 |
| **HLA-DRB1*16:02** | 712 | 725 | GKFVKISGSSIGEK | 0.06 |
| **HLA-DRB1*15:01** | 1418 | 1431 | GTELTQYTTAEVDT | 0.06 |
| **HLA-DRB1*15:01** | 449 | 462 | EAKLAKYQADLAKY | 0.06 |
| **HLA-DRB1*15:01** | 1236 | 1249 | GVSVDNYTNLEAAP | 0.06 |
| **HLA-DRB1*15:01** | 1510 | 1523 | TSTVIIYKPQSTAY | 0.06 |
| **HLA-DRB1*15:01** | 311 | 324 | AKLTAYQTELARVQ | 0.06 |
| **HLA-DRB1*15:01** | 393 | 406 | AKLTAYQTELARVQ | 0.06 |
| **HLA-DRB1*15:01** | 1418 | 1432 | GTELTQYTTAEVDTT | 0.06 |
| **HLA-DRB1*15:01** | 309 | 323 | YQAKLTAYQTELARV | 0.06 |
| **HLA-DRB1*15:01** | 391 | 405 | YQAKLTAYQTELARV | 0.06 |
| **HLA-DRB1*15:01** | 449 | 463 | EAKLAKYQADLAKYQ | 0.06 |
| **HLA-DRB1*04:01** | 623 | 636 | SKIVYKYTVDPKSK | 0.06 |
| **HLA-DRB1*04:01** | 149 | 162 | TDQYKSDVAAHEAE | 0.06 |
| **HLA-DRB1*04:01** | 939 | 952 | EPSYEAEPTPPTPT | 0.06 |
| **HLA-DRB1*04:01** | 962 | 975 | VEPTYEVIPTPPTD | 0.06 |
| **HLA-DRB1*04:01** | 1512 | 1526 | TVIIYKPQSTAYQPS | 0.06 |
| **HLA-DRB1*04:01** | 899 | 913 | VEPSYEAEPTPPTRT | 0.06 |
| **HLA-DRB1*07:01** | 711 | 725 | SGKFVKISGSSIGEK | 0.06 |
| **HLA-DRB1*04:01** | 34 | 48 | QKVFADETTTTSDVD | 0.06 |
| **HLA-DRB1*04:01** | 149 | 163 | TDQYKSDVAAHEAEV | 0.06 |
| **HLA-DRB1*11:01** | 373 | 387 | YEADLAAVKKANAAN | 0.06 |
| **HLA-DRB1*11:01** | 374 | 387 | EADLAAVKKANAAN | 0.06 |
| **HLA-DRB1*03:01** | 1010 | 1024 | NNDINIDRTLVAKQS | 0.06 |
| **HLA-DPA1*01:03/DPB1*04:02** | 1450 | 1463 | IDSAFQAESYIQMK | 0.06 |
| **HLA-DRB1*15:01** | 1235 | 1249 | TGVSVDNYTNLEAAP | 0.07 |
| **HLA-DRB1*15:01** | 367 | 381 | EAALKQYEADLAAVK | 0.07 |
| **HLA-DRB1*08:01** | 362 | 375 | AKATYEAALKQYEA | 0.07 |
| **HLA-DRB1*03:01** | 1166 | 1180 | NGVVIDGKTVLAGST | 0.07 |
| **HLA-DRB1*03:01** | 1009 | 1023 | NNNDINIDRTLVAKQ | 0.07 |
| **HLA-DRB1*03:01** | 1163 | 1177 | KNENGVVIDGKTVLA | 0.07 |
| **HLA-DRB1*11:01** | 1456 | 1469 | AESYIQMKRIAVGT | 0.07 |
| **HLA-DRB1*03:01** | 1164 | 1177 | NENGVVIDGKTVLA | 0.07 |
| **HLA-DPA1*02:01/DPB1*05:01** | 1450 | 1464 | IDSAFQAESYIQMKR | 0.07 |
| **HLA-DPA1*01:03/DPB1*02:01** | 988 | 1001 | PTVHFHYFKLAVQP | 0.07 |
| **HLA-DRB1*16:02** | 963 | 977 | EPTYEVIPTPPTDPV | 0.08 |
| **HLA-DRB1*15:01** | 470 | 484 | PVKLKAYEDEQTSIK | 0.08 |
| **HLA-DRB1*15:01** | 203 | 217 | EAKLAQYQADLAAVQ | 0.08 |
| **HLA-DRB1*15:01** | 1510 | 1524 | TSTVIIYKPQSTAYQ | 0.08 |
| **HLA-DRB1*07:01** | 710 | 724 | YSGKFVKISGSSIGE | 0.08 |
| **HLA-DRB1*07:01** | 711 | 724 | SGKFVKISGSSIGE | 0.08 |
| **HLA-DRB1*04:01** | 964 | 977 | PTYEVIPTPPTDPV | 0.08 |
| **HLA-DRB1*04:01** | 148 | 162 | TTDQYKSDVAAHEAE | 0.08 |
| **HLA-DRB1*04:01** | 938 | 952 | VEPSYEAEPTPPTPT | 0.08 |
| **HLA-DRB1*11:01** | 288 | 302 | LAQYQAELKRVQEAN | 0.08 |
| **HLA-DRB1*11:01** | 1455 | 1469 | QAESYIQMKRIAVGT | 0.08 |
| **HLA-DRB1*03:01** | 1166 | 1179 | NGVVIDGKTVLAGS | 0.08 |
| **HLA-DPA1*01:03/DPB1*04:02** | 1452 | 1465 | SAFQAESYIQMKRI | 0.08 |
| **HLA-DRB1*16:02** | 710 | 724 | YSGKFVKISGSSIGE | 0.09 |
| **HLA-DRB1*16:02** | 962 | 976 | VEPTYEVIPTPPTDP | 0.09 |
| **HLA-DRB1*15:01** | 1417 | 1431 | SGTELTQYTTAEVDT | 0.09 |
| **HLA-DRB1*04:01** | 623 | 637 | SKIVYKYTVDPKSKF | 0.09 |
| **HLA-DRB1*11:01** | 317 | 331 | QTELARVQKANADAK | 0.09 |
| **HLA-DRB1*11:01** | 399 | 413 | QTELARVQKANADAK | 0.09 |
| **HLA-DRB1*03:01** | 1010 | 1023 | NNDINIDRTLVAKQ | 0.09 |
| **HLA-DPA1*02:01/DPB1*05:01** | 1451 | 1465 | DSAFQAESYIQMKRI | 0.09 |
| **HLA-DPA1*01:03/DPB1*02:01** | 986 | 1000 | SDPTVHFHYFKLAVQ | 0.09 |
| **HLA-DRB1*01:01** | 710 | 723 | YSGKFVKISGSSIG | 0.09 |
| **HLA-DRB1*03:01** | 1009 | 1022 | NNNDINIDRTLVAK | 0.1 |
| **HLA-DRB1*03:01** | 1011 | 1024 | NDINIDRTLVAKQS | 0.1 |
| **HLA-DRB1*11:01** | 231 | 245 | LAAYQAELKRVQEAN | 0.1 |
| **HLA-DRB1*16:02** | 743 | 757 | GSRWTMYKNSQAGSG | 0.11 |
| **HLA-DRB1*15:01** | 1237 | 1250 | VSVDNYTNLEAAPQ | 0.11 |
| **HLA-DRB1*04:01** | 901 | 914 | PSYEAEPTPPTRTP | 0.11 |
| **HLA-DRB1*04:01** | 1514 | 1527 | IIYKPQSTAYQPSS | 0.11 |
| **HLA-DRB1*04:01** | 1512 | 1525 | TVIIYKPQSTAYQP | 0.11 |
| **HLA-DRB1*04:01** | 34 | 47 | QKVFADETTTTSDV | 0.11 |
| **HLA-DRB1*04:01** | 961 | 975 | PVEPTYEVIPTPPTD | 0.11 |
| **HLA-DRB1*11:01** | 1455 | 1468 | QAESYIQMKRIAVG | 0.11 |
| **HLA-DRB1*11:01** | 288 | 301 | LAQYQAELKRVQEA | 0.11 |
| **HLA-DRB1*11:01** | 375 | 388 | ADLAAVKKANAANE | 0.11 |
| **HLA-DRB1*16:02** | 743 | 756 | GSRWTMYKNSQAGS | 0.12 |
| **HLA-DRB1*15:01** | 1511 | 1524 | STVIIYKPQSTAYQ | 0.12 |
| **HLA-DRB1*15:01** | 203 | 216 | EAKLAQYQADLAAV | 0.12 |
| **HLA-DRB1*15:01** | 1509 | 1522 | RTSTVIIYKPQSTA | 0.12 |
| **HLA-DRB1*15:01** | 448 | 462 | YEAKLAKYQADLAKY | 0.12 |
| **HLA-DRB1*04:01** | 899 | 912 | VEPSYEAEPTPPTR | 0.12 |
| **HLA-DRB1*03:01** | 517 | 530 | NLSLTTDGKFLKAS | 0.12 |
| **HLA-DRB1*03:01** | 682 | 696 | EKPINFDNALLSVTS | 0.12 |
| **HLA-DRB1*03:01** | 517 | 531 | NLSLTTDGKFLKASA | 0.12 |
| **HLA-DPA1*02:01/DPB1*05:01** | 987 | 1001 | DPTVHFHYFKLAVQP | 0.12 |
| **HLA-DPA1*01:03/DPB1*02:01** | 988 | 1002 | PTVHFHYFKLAVQPQ | 0.12 |
| **HLA-DRB1*15:01** | 1509 | 1523 | RTSTVIIYKPQSTAY | 0.13 |
| **HLA-DRB1*15:01** | 308 | 322 | DYQAKLTAYQTELAR | 0.13 |
| **HLA-DRB1*15:01** | 390 | 404 | DYQAKLTAYQTELAR | 0.13 |
| **HLA-DRB1*04:01** | 625 | 638 | IVYKYTVDPKSKFQ | 0.13 |
| **HLA-DRB1*04:01** | 938 | 951 | VEPSYEAEPTPPTP | 0.13 |
| **HLA-DRB1*04:01** | 940 | 953 | PSYEAEPTPPTPTP | 0.13 |
| **HLA-DRB1*03:01** | 682 | 695 | EKPINFDNALLSVT | 0.13 |
| **HLA-DRB1*04:01** | 33 | 47 | GQKVFADETTTTSDV | 0.13 |
| **HLA-DRB1*03:01** | 1008 | 1022 | RNNNDINIDRTLVAK | 0.13 |
| **HLA-DRB1*11:01** | 1456 | 1470 | AESYIQMKRIAVGTF | 0.13 |
| **HLA-DRB1*11:01** | 373 | 386 | YEADLAAVKKANAA | 0.13 |
| **HLA-DRB1*16:02** | 710 | 723 | YSGKFVKISGSSIG | 0.14 |
| **HLA-DRB1*16:02** | 742 | 756 | GGSRWTMYKNSQAGS | 0.14 |
| **HLA-DRB1*11:01** | 289 | 302 | AQYQAELKRVQEAN | 0.14 |
| **HLA-DRB1*11:01** | 231 | 244 | LAAYQAELKRVQEA | 0.14 |
| **HLA-DRB1*01:01** | 712 | 726 | GKFVKISGSSIGEKN | 0.14 |
| **HLA-DRB1*04:01** | 1054 | 1067 | GYQFNPEATKAASP | 0.15 |
| **HLA-DRB1*04:01** | 148 | 161 | TTDQYKSDVAAHEA | 0.15 |
| **HLA-DRB1*04:01** | 150 | 163 | DQYKSDVAAHEAEV | 0.15 |
| **HLA-DRB1*04:01** | 33 | 46 | GQKVFADETTTTSD | 0.15 |
| **HLA-DRB1*04:01** | 622 | 636 | ISKIVYKYTVDPKSK | 0.15 |
| **HLA-DRB1*04:01** | 1008 | 1022 | RNNNDINIDRTLVAK | 0.15 |
| **HLA-DRB1*11:01** | 232 | 245 | AAYQAELKRVQEAN | 0.15 |
| **HLA-DRB1*15:01** | 450 | 463 | AKLAKYQADLAKYQ | 0.16 |
| **HLA-DRB1*15:01** | 1419 | 1432 | TELTQYTTAEVDTT | 0.16 |
| **HLA-DRB1*15:01** | 367 | 380 | EAALKQYEADLAAV | 0.16 |
| **HLA-DRB1*15:01** | 448 | 461 | YEAKLAKYQADLAK | 0.16 |
| **HLA-DRB1*04:01** | 975 | 989 | DPVYQDLPTPPSDPT | 0.16 |
| **HLA-DPA1*01:03/DPB1*02:01** | 986 | 999 | SDPTVHFHYFKLAV | 0.16 |
| **HLA-DRB1*16:02** | 962 | 975 | VEPTYEVIPTPPTD | 0.17 |
| **HLA-DRB1*16:02** | 964 | 977 | PTYEVIPTPPTDPV | 0.17 |
| **HLA-DRB1*16:02** | 712 | 726 | GKFVKISGSSIGEKN | 0.17 |
| **HLA-DRB1*04:01** | 975 | 988 | DPVYQDLPTPPSDP | 0.17 |
| **HLA-DRB1*07:01** | 710 | 723 | YSGKFVKISGSSIG | 0.17 |
| **HLA-DRB1*04:01** | 1011 | 1025 | NDINIDRTLVAKQSV | 0.17 |
| **HLA-DRB1*04:01** | 1054 | 1068 | GYQFNPEATKAASPG | 0.17 |
| **HLA-DRB1*04:01** | 964 | 978 | PTYEVIPTPPTDPVY | 0.17 |
| **HLA-DRB1*11:01** | 317 | 330 | QTELARVQKANADA | 0.17 |
| **HLA-DRB1*11:01** | 399 | 412 | QTELARVQKANADA | 0.17 |
| **HLA-DRB1*15:01** | 202 | 216 | YEAKLAQYQADLAAV | 0.18 |
| **HLA-DRB1*04:01** | 974 | 988 | TDPVYQDLPTPPSDP | 0.18 |
| **HLA-DRB1*04:01** | 861 | 875 | APNYEKEPTPPTRTP | 0.18 |
| **HLA-DRB1*03:01** | 516 | 530 | ANLSLTTDGKFLKAS | 0.18 |
| **HLA-DPA1*01:03/DPB1*02:01** | 1451 | 1464 | DSAFQAESYIQMKR | 0.18 |
| **HLA-DRB1*04:01** | 35 | 48 | KVFADETTTTSDVD | 0.19 |
| **HLA-DRB1*04:01** | 207 | 220 | AQYQADLAAVQKTN | 0.19 |
| **HLA-DRB1*07:01** | 712 | 725 | GKFVKISGSSIGEK | 0.19 |
| **HLA-DRB1*04:01** | 898 | 912 | PVEPSYEAEPTPPTR | 0.19 |
| **HLA-DRB1*08:01** | 361 | 375 | NAKATYEAALKQYEA | 0.19 |
| **HLA-DRB1*11:01** | 287 | 301 | KLAQYQAELKRVQEA | 0.19 |
| **HLA-DPA1*01:03/DPB1*04:02** | 1449 | 1463 | SIDSAFQAESYIQMK | 0.19 |
| **HLA-DRB1*16:02** | 961 | 975 | PVEPTYEVIPTPPTD | 0.2 |
| **HLA-DRB1*15:01** | 1417 | 1430 | SGTELTQYTTAEVD | 0.2 |
| **HLA-DRB1*15:01** | 470 | 483 | PVKLKAYEDEQTSI | 0.2 |
| **HLA-DRB1*04:01** | 206 | 219 | LAQYQADLAAVQKT | 0.2 |
| **HLA-DRB1*04:01** | 901 | 915 | PSYEAEPTPPTRTPD | 0.2 |
| **HLA-DRB1*04:01** | 1511 | 1525 | STVIIYKPQSTAYQP | 0.2 |
| **HLA-DRB1*04:01** | 1053 | 1067 | SGYQFNPEATKAASP | 0.2 |
| **HLA-DRB1*04:01** | 206 | 220 | LAQYQADLAAVQKTN | 0.2 |
| **HLA-DRB1*04:01** | 937 | 951 | PVEPSYEAEPTPPTP | 0.2 |
| **HLA-DRB1*03:01** | 1011 | 1025 | NDINIDRTLVAKQSV | 0.2 |
| **HLA-DRB1*03:01** | 681 | 695 | DEKPINFDNALLSVT | 0.2 |
| **HLA-DPA1*01:03/DPB1*02:01** | 985 | 999 | PSDPTVHFHYFKLAV | 0.2 |
| **HLA-DRB1*15:01** | 311 | 325 | AKLTAYQTELARVQK | 0.21 |
| **HLA-DRB1*15:01** | 393 | 407 | AKLTAYQTELARVQK | 0.21 |
| **HLA-DRB1*15:01** | 366 | 380 | YEAALKQYEADLAAV | 0.21 |
| **HLA-DRB1*07:01** | 1079 | 1093 | TVTFKATAATLATFN | 0.21 |
| **HLA-DRB1*13:01** | 374 | 388 | EADLAAVKKANAANE | 0.21 |
| **HLA-DRB1*04:01** | 32 | 46 | AGQKVFADETTTTSD | 0.21 |
| **HLA-DRB1*04:01** | 940 | 954 | PSYEAEPTPPTPTPD | 0.21 |
| **HLA-DRB1*11:01** | 372 | 386 | QYEADLAAVKKANAA | 0.21 |
| **HLA-DRB1*11:01** | 375 | 389 | ADLAAVKKANAANEA | 0.21 |
| **HLA-DPA1*02:01/DPB1*05:01** | 1450 | 1463 | IDSAFQAESYIQMK | 0.22 |
| **HLA-DRB1*15:01** | 1235 | 1248 | TGVSVDNYTNLEAA | 0.23 |
| **HLA-DRB1*07:01** | 1078 | 1091 | NTVTFKATAATLAT | 0.23 |
| **HLA-DRB1*03:01** | 516 | 529 | ANLSLTTDGKFLKA | 0.23 |
| **HLA-DRB1*16:02** | 744 | 757 | SRWTMYKNSQAGSG | 0.24 |
| **HLA-DRB1*16:02** | 742 | 755 | GGSRWTMYKNSQAG | 0.24 |
| **HLA-DRB1*08:01** | 374 | 388 | EADLAAVKKANAANE | 0.24 |
| **HLA-DRB1*11:01** | 230 | 244 | ALAAYQAELKRVQEA | 0.24 |
| **HLA-DRB1*16:02** | 964 | 978 | PTYEVIPTPPTDPVY | 0.25 |
| **HLA-DRB1*15:01** | 1237 | 1251 | VSVDNYTNLEAAPQE | 0.25 |
| **HLA-DRB1*11:01** | 287 | 300 | KLAQYQAELKRVQE | 0.25 |
| **HLA-DRB1*11:01** | 1457 | 1470 | ESYIQMKRIAVGTF | 0.25 |
| **HLA-DRB1*11:01** | 1454 | 1468 | FQAESYIQMKRIAVG | 0.25 |
| **HLA-DRB1*11:01** | 316 | 330 | YQTELARVQKANADA | 0.25 |
| **HLA-DRB1*11:01** | 398 | 412 | YQTELARVQKANADA | 0.25 |
| **HLA-DRB1*15:01** | 368 | 381 | AALKQYEADLAAVK | 0.26 |
| **HLA-DRB1*07:01** | 709 | 723 | DYSGKFVKISGSSIG | 0.26 |
| **HLA-DRB1*04:01** | 147 | 161 | KTTDQYKSDVAAHEA | 0.26 |
| **HLA-DPA1*01:03/DPB1*02:01** | 1450 | 1464 | IDSAFQAESYIQMKR | 0.26 |
| **HLA-DRB1*16:02** | 709 | 723 | DYSGKFVKISGSSIG | 0.27 |
| **HLA-DRB1*04:01** | 1220 | 1234 | RQDLVKITDANGNEV | 0.27 |
| **HLA-DRB1*04:01** | 1514 | 1528 | IIYKPQSTAYQPSSV | 0.27 |
| **HLA-DRB1*04:01** | 860 | 874 | VAPNYEKEPTPPTRT | 0.27 |
| **HLA-DPA1*01:03/DPB1*02:01** | 1451 | 1465 | DSAFQAESYIQMKRI | 0.27 |
| **HLA-DRB1*04:01** | 861 | 874 | APNYEKEPTPPTRT | 0.28 |
| **HLA-DRB1*04:01** | 579 | 593 | KAGWSTTVSNNSQVK | 0.28 |
| **HLA-DRB1*04:01** | 1042 | 1056 | TTSFVLVDPLPSGYQ | 0.28 |
| **HLA-DRB1*11:01** | 318 | 331 | TELARVQKANADAK | 0.28 |
| **HLA-DRB1*11:01** | 400 | 413 | TELARVQKANADAK | 0.28 |
| **HLA-DRB1*15:01** | 469 | 483 | YPVKLKAYEDEQTSI | 0.29 |
| **HLA-DRB1*11:01** | 1392 | 1406 | GDHYTGQYKVFAKVD | 0.29 |
| **HLA-DRB1*15:01** | 202 | 215 | YEAKLAQYQADLAA | 0.3 |
| **HLA-DRB1*07:01** | 1079 | 1092 | TVTFKATAATLATF | 0.3 |
| **HLA-DRB1*04:01** | 1219 | 1233 | LRQDLVKITDANGNE | 0.3 |
| **HLA-DRB1*04:01** | 1042 | 1055 | TTSFVLVDPLPSGY | 0.3 |
| **HLA-DRB1*04:01** | 150 | 164 | DQYKSDVAAHEAEVA | 0.3 |
| **HLA-DRB1*08:01** | 364 | 377 | ATYEAALKQYEADL | 0.3 |
| **HLA-DRB1*01:01** | 1079 | 1093 | TVTFKATAATLATFN | 0.3 |
| **HLA-DRB1*04:01** | 625 | 639 | IVYKYTVDPKSKFQG | 0.31 |
| **HLA-DRB1*16:02** | 819 | 833 | NGKIRAVNVPKVTKE | 0.32 |
| **HLA-DRB1*07:01** | 1077 | 1091 | TNTVTFKATAATLAT | 0.32 |
| **HLA-DRB1*07:01** | 1078 | 1092 | NTVTFKATAATLATF | 0.32 |
| **HLA-DRB1*15:01** | 819 | 833 | NGKIRAVNVPKVTKE | 0.32 |
| **HLA-DRB1*08:01** | 306 | 319 | EADYQAKLTAYQTE | 0.32 |
| **HLA-DRB1*08:01** | 388 | 401 | EADYQAKLTAYQTE | 0.32 |
| **HLA-DRB1*11:01** | 230 | 243 | ALAAYQAELKRVQE | 0.32 |
| **HLA-DRB1*01:01** | 331 | 345 | KATYEAAVAANNAKN | 0.32 |
| **HLA-DRB1*01:01** | 709 | 723 | DYSGKFVKISGSSIG | 0.32 |
| **HLA-DRB1*16:02** | 819 | 832 | NGKIRAVNVPKVTK | 0.33 |
| **HLA-DRB1*07:01** | 712 | 726 | GKFVKISGSSIGEKN | 0.33 |
| **HLA-DRB1*15:01** | 1508 | 1522 | PRTSTVIIYKPQSTA | 0.33 |
| **HLA-DRB1*04:01** | 682 | 696 | EKPINFDNALLSVTS | 0.33 |
| **HLA-DRB1*08:01** | 374 | 387 | EADLAAVKKANAAN | 0.33 |
| **HLA-DRB1*15:01** | 819 | 832 | NGKIRAVNVPKVTK | 0.34 |
| **HLA-DRB1*15:01** | 366 | 379 | YEAALKQYEADLAA | 0.34 |
| **HLA-DRB1*15:01** | 204 | 217 | AKLAQYQADLAAVQ | 0.34 |
| **HLA-DRB1*03:01** | 518 | 531 | LSLTTDGKFLKASA | 0.34 |
| **HLA-DRB1*03:01** | 681 | 694 | DEKPINFDNALLSV | 0.34 |
| **HLA-DPA1*01:03/DPB1*04:02** | 987 | 1001 | DPTVHFHYFKLAVQP | 0.34 |
| **HLA-DRB1*01:01** | 963 | 977 | EPTYEVIPTPPTDPV | 0.34 |
| **HLA-DRB1*08:01** | 373 | 387 | YEADLAAVKKANAAN | 0.35 |
| **HLA-DRB1*08:01** | 305 | 319 | NEADYQAKLTAYQTE | 0.35 |
| **HLA-DRB1*08:01** | 387 | 401 | NEADYQAKLTAYQTE | 0.35 |
| **HLA-DRB1*03:01** | 1163 | 1176 | KNENGVVIDGKTVL | 0.35 |
| **HLA-DRB1*01:01** | 1079 | 1092 | TVTFKATAATLATF | 0.35 |
| **HLA-DRB1*07:01** | 819 | 833 | NGKIRAVNVPKVTKE | 0.36 |
| **HLA-DRB1*15:01** | 447 | 461 | DYEAKLAKYQADLAK | 0.36 |
| **HLA-DRB1*04:01** | 35 | 49 | KVFADETTTTSDVDT | 0.36 |
| **HLA-DRB1*04:01** | 205 | 219 | KLAQYQADLAAVQKT | 0.36 |
| **HLA-DRB1*04:01** | 1220 | 1233 | RQDLVKITDANGNE | 0.37 |
| **HLA-DRB1*13:01** | 1433 | 1447 | KGAITIKFKEAFLRS | 0.37 |
| **HLA-DRB1*15:01** | 471 | 484 | VKLKAYEDEQTSIK | 0.38 |
| **HLA-DRB1*04:01** | 1189 | 1203 | LDQYKNDRSSADTIQ | 0.38 |
| **HLA-DPA1*01:03/DPB1*04:02** | 1452 | 1466 | SAFQAESYIQMKRIA | 0.38 |
| **HLA-DRB1*04:01** | 682 | 695 | EKPINFDNALLSVT | 0.39 |
| **HLA-DRB1*04:01** | 974 | 987 | TDPVYQDLPTPPSD | 0.39 |
| **HLA-DRB1*04:01** | 976 | 989 | PVYQDLPTPPSDPT | 0.39 |
| **HLA-DRB1*04:01** | 862 | 875 | PNYEKEPTPPTRTP | 0.39 |
| **HLA-DRB1*03:01** | 683 | 696 | KPINFDNALLSVTS | 0.39 |
| **HLA-DRB1*11:01** | 1392 | 1405 | GDHYTGQYKVFAKV | 0.39 |
| **HLA-DRB1*16:02** | 741 | 755 | EGGSRWTMYKNSQAG | 0.4 |
| **HLA-DRB1*04:01** | 1055 | 1068 | YQFNPEATKAASPG | 0.4 |
| **HLA-DRB1*03:01** | 626 | 639 | VYKYTVDPKSKFQG | 0.4 |
| **HLA-DRB1*11:01** | 210 | 224 | QADLAAVQKTNAANQ | 0.4 |
| **HLA-DRB1*15:01** | 743 | 756 | GSRWTMYKNSQAGS | 0.41 |
| **HLA-DRB1*15:01** | 1416 | 1430 | KSGTELTQYTTAEVD | 0.41 |
| **HLA-DRB1*03:01** | 680 | 694 | EDEKPINFDNALLSV | 0.41 |
| **HLA-DRB1*15:01** | 469 | 482 | YPVKLKAYEDEQTS | 0.42 |
| **HLA-DRB1*04:01** | 1041 | 1054 | ETTSFVLVDPLPSG | 0.42 |
| **HLA-DRB1*08:01** | 1456 | 1469 | AESYIQMKRIAVGT | 0.42 |
| **HLA-DRB1*11:01** | 289 | 303 | AQYQAELKRVQEANA | 0.42 |
| **HLA-DPA1*02:01/DPB1*05:01** | 988 | 1001 | PTVHFHYFKLAVQP | 0.42 |
| **HLA-DPA1*01:03/DPB1*04:02** | 987 | 1000 | DPTVHFHYFKLAVQ | 0.42 |
| **HLA-DRB1*01:01** | 963 | 976 | EPTYEVIPTPPTDP | 0.42 |
| **HLA-DRB1*13:01** | 373 | 387 | YEADLAAVKKANAAN | 0.43 |
| **HLA-DRB1*03:01** | 515 | 529 | NANLSLTTDGKFLKA | 0.43 |
| **HLA-DRB1*16:02** | 624 | 638 | KIVYKYTVDPKSKFQ | 0.44 |
| **HLA-DRB1*07:01** | 819 | 832 | NGKIRAVNVPKVTK | 0.44 |
| **HLA-DRB1*04:01** | 205 | 218 | KLAQYQADLAAVQK | 0.44 |
| **HLA-DRB1*04:01** | 860 | 873 | VAPNYEKEPTPPTR | 0.44 |
| **HLA-DRB1*03:01** | 627 | 640 | YKYTVDPKSKFQGQ | 0.44 |
| **HLA-DRB1*03:01** | 626 | 640 | VYKYTVDPKSKFQGQ | 0.44 |
| **HLA-DRB1*16:02** | 624 | 637 | KIVYKYTVDPKSKF | 0.45 |
| **HLA-DRB1*15:01** | 1419 | 1433 | TELTQYTTAEVDTTK | 0.45 |
| **HLA-DRB1*01:01** | 962 | 976 | VEPTYEVIPTPPTDP | 0.45 |
| **HLA-DRB1*04:01** | 1053 | 1066 | SGYQFNPEATKAAS | 0.46 |
| **HLA-DRB1*13:01** | 374 | 387 | EADLAAVKKANAAN | 0.46 |
| **HLA-DPA1*02:01/DPB1*05:01** | 987 | 1000 | DPTVHFHYFKLAVQ | 0.46 |
| **HLA-DRB1*01:01** | 413 | 427 | KAAYEAAVAANNAAN | 0.46 |
| **HLA-DRB1*08:01** | 373 | 386 | YEADLAAVKKANAA | 0.47 |
| **HLA-DRB1*08:01** | 305 | 318 | NEADYQAKLTAYQT | 0.47 |
| **HLA-DRB1*08:01** | 387 | 400 | NEADYQAKLTAYQT | 0.47 |
| **HLA-DRB1*04:01** | 973 | 987 | PTDPVYQDLPTPPSD | 0.47 |
| **HLA-DRB1*03:01** | 1167 | 1180 | GVVIDGKTVLAGST | 0.47 |
| **HLA-DPA1*01:03/DPB1*02:01** | 1450 | 1463 | IDSAFQAESYIQMK | 0.47 |
| **HLA-DRB1*07:01** | 818 | 832 | LNGKIRAVNVPKVTK | 0.48 |
| **HLA-DRB1*15:01** | 1234 | 1248 | VTGVSVDNYTNLEAA | 0.48 |
| **HLA-DRB1*01:01** | 331 | 344 | KATYEAAVAANNAK | 0.48 |
| **HLA-DRB1*08:01** | 306 | 320 | EADYQAKLTAYQTEL | 0.49 |
| **HLA-DRB1*08:01** | 388 | 402 | EADYQAKLTAYQTEL | 0.49 |
| **HLA-DPA1*02:01/DPB1*05:01** | 988 | 1002 | PTVHFHYFKLAVQPQ | 0.49 |
| **HLA-DRB1*15:01** | 1511 | 1525 | STVIIYKPQSTAYQP | 0.5 |
| **HLA-DRB1*13:01** | 628 | 642 | KYTVDPKSKFQGQKV | 0.5 |
| **HLA-DRB1*03:01** | 625 | 638 | IVYKYTVDPKSKFQ | 0.5 |
| **HLA-DRB1*03:01** | 625 | 639 | IVYKYTVDPKSKFQG | 0.5 |
| **HLA-DRB1*11:01** | 232 | 246 | AAYQAELKRVQEANA | 0.5 |
| **HLA-DRB1*08:01** | 1455 | 1469 | QAESYIQMKRIAVGT | 0.51 |
| **HLA-DPA1*01:03/DPB1*04:02** | 988 | 1001 | PTVHFHYFKLAVQP | 0.51 |
| **HLA-DPA1*01:03/DPB1*02:01** | 1452 | 1465 | SAFQAESYIQMKRI | 0.51 |
| **HLA-DRB1*01:01** | 330 | 344 | AKATYEAAVAANNAK | 0.51 |
| **HLA-DRB1*07:01** | 1080 | 1093 | VTFKATAATLATFN | 0.52 |
| **HLA-DRB1*08:01** | 1455 | 1468 | QAESYIQMKRIAVG | 0.52 |
| **HLA-DRB1*16:02** | 744 | 758 | SRWTMYKNSQAGSGW | 0.53 |
| **HLA-DRB1*13:01** | 1434 | 1447 | GAITIKFKEAFLRS | 0.53 |
| **HLA-DRB1*11:01** | 210 | 223 | QADLAAVQKTNAAN | 0.53 |
| **HLA-DRB1*11:01** | 316 | 329 | YQTELARVQKANAD | 0.53 |
| **HLA-DRB1*11:01** | 398 | 411 | YQTELARVQKANAD | 0.53 |
| **HLA-DPA1*01:03/DPB1*02:01** | 985 | 998 | PSDPTVHFHYFKLA | 0.53 |
| **HLA-DRB1*07:01** | 811 | 824 | KPNIWYSLNGKIRA | 0.54 |
| **HLA-DPA1*01:03/DPB1*02:01** | 989 | 1002 | TVHFHYFKLAVQPQ | 0.54 |
| **HLA-DRB1*15:01** | 744 | 757 | SRWTMYKNSQAGSG | 0.55 |
| **HLA-DRB1*04:01** | 1043 | 1056 | TSFVLVDPLPSGYQ | 0.55 |
| **HLA-DRB1*04:01** | 1189 | 1202 | LDQYKNDRSSADTI | 0.55 |
| **HLA-DRB1*04:01** | 579 | 592 | KAGWSTTVSNNSQV | 0.55 |
| **HLA-DRB1*04:01** | 1239 | 1253 | VDNYTNLEAAPQEIR | 0.55 |
| **HLA-DRB1*04:01** | 1041 | 1055 | ETTSFVLVDPLPSGY | 0.55 |
| **HLA-DRB1*15:01** | 201 | 215 | AYEAKLAQYQADLAA | 0.56 |
| **HLA-DRB1*15:01** | 818 | 832 | LNGKIRAVNVPKVTK | 0.56 |
| **HLA-DRB1*15:01** | 743 | 757 | GSRWTMYKNSQAGSG | 0.56 |
| **HLA-DRB1*13:01** | 317 | 331 | QTELARVQKANADAK | 0.56 |
| **HLA-DRB1*13:01** | 399 | 413 | QTELARVQKANADAK | 0.56 |
| **HLA-DRB1*11:01** | 363 | 376 | KATYEAALKQYEAD | 0.56 |
| **HLA-DRB1*01:01** | 413 | 426 | KAAYEAAVAANNAA | 0.56 |
| **HLA-DRB1*01:01** | 1239 | 1253 | VDNYTNLEAAPQEIR | 0.56 |
| **HLA-DRB1*01:01** | 1078 | 1092 | NTVTFKATAATLATF | 0.56 |
| **HLA-DRB1*15:01** | 820 | 833 | GKIRAVNVPKVTKE | 0.57 |
| **HLA-DRB1*08:01** | 375 | 388 | ADLAAVKKANAANE | 0.57 |
| **HLA-DPA1*02:01/DPB1*05:01** | 374 | 388 | EADLAAVKKANAANE | 0.57 |
| **HLA-DPA1*01:03/DPB1*04:02** | 986 | 1000 | SDPTVHFHYFKLAVQ | 0.57 |
| **HLA-DRB1*15:01** | 742 | 755 | GGSRWTMYKNSQAG | 0.58 |
| **HLA-DRB1*13:01** | 1433 | 1446 | KGAITIKFKEAFLR | 0.58 |
| **HLA-DRB1*01:01** | 412 | 426 | AKAAYEAAVAANNAA | 0.58 |
| **HLA-DRB1*16:02** | 1079 | 1093 | TVTFKATAATLATFN | 0.59 |
| **HLA-DRB1*15:01** | 649 | 662 | DPTLGVFASAYTGQ | 0.59 |
| **HLA-DRB1*07:01** | 818 | 831 | LNGKIRAVNVPKVT | 0.59 |
| **HLA-DRB1*04:01** | 1190 | 1203 | DQYKNDRSSADTIQ | 0.59 |
| **HLA-DRB1*15:01** | 450 | 464 | AKLAKYQADLAKYQK | 0.59 |
| **HLA-DRB1*01:01** | 1080 | 1093 | VTFKATAATLATFN | 0.59 |
| **HLA-DRB1*01:01** | 1078 | 1091 | NTVTFKATAATLAT | 0.59 |
| **HLA-DRB1*16:02** | 818 | 832 | LNGKIRAVNVPKVTK | 0.6 |
| **HLA-DRB1*15:01** | 365 | 379 | TYEAALKQYEADLAA | 0.6 |
| **HLA-DRB1*04:01** | 976 | 990 | PVYQDLPTPPSDPTV | 0.6 |
| **HLA-DRB1*11:01** | 286 | 300 | TKLAQYQAELKRVQE | 0.6 |
| **HLA-DRB1*11:01** | 1391 | 1405 | TGDHYTGQYKVFAKV | 0.6 |
| **HLA-DRB1*04:01** | 452 | 465 | LAKYQADLAKYQKD | 0.61 |
| **HLA-DRB1*04:01** | 207 | 221 | AQYQADLAAVQKTNA | 0.61 |
| **HLA-DRB1*03:01** | 683 | 697 | KPINFDNALLSVTSL | 0.61 |
| **HLA-DRB1*03:01** | 1162 | 1176 | NKNENGVVIDGKTVL | 0.61 |
| **HLA-DPA1*02:01/DPB1*05:01** | 1452 | 1465 | SAFQAESYIQMKRI | 0.61 |
| **HLA-DRB1*04:01** | 131 | 144 | ETEIKEDYTKQAED | 0.62 |
| **HLA-DRB1*11:01** | 362 | 376 | AKATYEAALKQYEAD | 0.62 |
| **HLA-DRB1*13:01** | 627 | 641 | YKYTVDPKSKFQGQK | 0.63 |
| **HLA-DRB1*11:01** | 1042 | 1056 | TTSFVLVDPLPSGYQ | 0.63 |
| **HLA-DRB1*11:01** | 318 | 332 | TELARVQKANADAKA | 0.64 |
| **HLA-DRB1*11:01** | 400 | 414 | TELARVQKANADAKA | 0.64 |
| **HLA-DRB1*11:01** | 209 | 223 | YQADLAAVQKTNAAN | 0.64 |
| **HLA-DPA1*01:03/DPB1*04:02** | 986 | 999 | SDPTVHFHYFKLAV | 0.64 |
| **HLA-DRB1*15:01** | 468 | 482 | DYPVKLKAYEDEQTS | 0.65 |
| **HLA-DRB1*15:01** | 368 | 382 | AALKQYEADLAAVKK | 0.65 |
| **HLA-DRB1*15:01** | 742 | 756 | GGSRWTMYKNSQAGS | 0.65 |
| **HLA-DRB1*08:01** | 199 | 212 | KTAYEAKLAQYQAD | 0.65 |
| **HLA-DRB1*03:01** | 644 | 657 | LGIFTDPTLGVFAS | 0.65 |
| **HLA-DRB1*03:01** | 518 | 532 | LSLTTDGKFLKASAV | 0.65 |
| **HLA-DPA1*01:03/DPB1*02:01** | 1394 | 1407 | HYTGQYKVFAKVDI | 0.65 |
| **HLA-DRB1*07:01** | 1077 | 1090 | TNTVTFKATAATLA | 0.66 |
| **HLA-DRB1*11:01** | 1042 | 1055 | TTSFVLVDPLPSGY | 0.66 |
| **HLA-DPA1*02:01/DPB1*05:01** | 819 | 833 | NGKIRAVNVPKVTKE | 0.66 |
| **HLA-DRB1*04:01** | 1239 | 1252 | VDNYTNLEAAPQEI | 0.67 |
| **HLA-DRB1*08:01** | 963 | 977 | EPTYEVIPTPPTDPV | 0.67 |
| **HLA-DRB1*03:01** | 627 | 641 | YKYTVDPKSKFQGQK | 0.67 |
| **HLA-DRB1*16:02** | 1079 | 1092 | TVTFKATAATLATF | 0.68 |
| **HLA-DRB1*07:01** | 709 | 722 | DYSGKFVKISGSSI | 0.68 |
| **HLA-DRB1*13:01** | 1322 | 1336 | ASNIVINNVPKINPK | 0.68 |
| **HLA-DRB1*11:01** | 1391 | 1404 | TGDHYTGQYKVFAK | 0.69 |
| **HLA-DRB1*01:01** | 964 | 977 | PTYEVIPTPPTDPV | 0.69 |
| **HLA-DRB1*16:02** | 623 | 636 | SKIVYKYTVDPKSK | 0.7 |
| **HLA-DRB1*16:02** | 623 | 637 | SKIVYKYTVDPKSKF | 0.7 |
| **HLA-DRB1*15:01** | 649 | 663 | DPTLGVFASAYTGQV | 0.7 |
| **HLA-DPA1*01:03/DPB1*02:01** | 1394 | 1408 | HYTGQYKVFAKVDIT | 0.7 |
| **HLA-DRB1*07:01** | 820 | 833 | GKIRAVNVPKVTKE | 0.71 |
| **HLA-DRB1*08:01** | 963 | 976 | EPTYEVIPTPPTDP | 0.71 |
| **HLA-DRB1*04:01** | 1219 | 1232 | LRQDLVKITDANGN | 0.72 |
| **HLA-DRB1*13:01** | 1435 | 1448 | AITIKFKEAFLRSV | 0.72 |
| **HLA-DRB1*08:01** | 317 | 331 | QTELARVQKANADAK | 0.72 |
| **HLA-DRB1*08:01** | 399 | 413 | QTELARVQKANADAK | 0.72 |
| **HLA-DRB1*08:01** | 364 | 378 | ATYEAALKQYEADLA | 0.72 |
| **HLA-DRB1*13:01** | 1435 | 1449 | AITIKFKEAFLRSVS | 0.72 |
| **HLA-DRB1*01:01** | 1042 | 1056 | TTSFVLVDPLPSGYQ | 0.72 |
| **HLA-DRB1*07:01** | 810 | 823 | KKPNIWYSLNGKIR | 0.73 |
| **HLA-DRB1*03:01** | 405 | 419 | VQKANADAKAAYEAA | 0.73 |
| **HLA-DRB1*04:01** | 1203 | 1216 | QKGFYYVDDYPEEA | 0.74 |
| **HLA-DRB1*04:01** | 819 | 832 | NGKIRAVNVPKVTK | 0.74 |
| **HLA-DRB1*04:01** | 681 | 694 | DEKPINFDNALLSV | 0.74 |
| **HLA-DRB1*15:01** | 204 | 218 | AKLAQYQADLAAVQK | 0.74 |
| **HLA-DRB1*07:01** | 764 | 778 | PNSWYGAGAIKMSGP | 0.74 |
| **HLA-DRB1*13:01** | 628 | 641 | KYTVDPKSKFQGQK | 0.74 |
| **HLA-DRB1*13:01** | 375 | 388 | ADLAAVKKANAANE | 0.74 |
| **HLA-DRB1*04:01** | 859 | 873 | PVAPNYEKEPTPPTR | 0.74 |
| **HLA-DRB1*04:01** | 681 | 695 | DEKPINFDNALLSVT | 0.74 |
| **HLA-DRB1*03:01** | 643 | 657 | WLGIFTDPTLGVFAS | 0.74 |
| **HLA-DRB1*11:01** | 229 | 243 | KALAAYQAELKRVQE | 0.74 |
| **HLA-DPA1*02:01/DPB1*05:01** | 541 | 554 | SKAKYDQKILQLDD | 0.74 |
| **HLA-DRB1*08:01** | 962 | 976 | VEPTYEVIPTPPTDP | 0.75 |
| **HLA-DRB1*11:01** | 363 | 377 | KATYEAALKQYEADL | 0.75 |
| **HLA-DRB1*01:01** | 332 | 345 | ATYEAAVAANNAKN | 0.75 |
| **HLA-DRB1*16:02** | 820 | 833 | GKIRAVNVPKVTKE | 0.76 |
| **HLA-DRB1*04:01** | 1381 | 1394 | EYNFYDDYDQTGDH | 0.76 |
| **HLA-DRB1*08:01** | 372 | 386 | QYEADLAAVKKANAA | 0.76 |
| **HLA-DRB1*08:01** | 304 | 318 | ANEADYQAKLTAYQT | 0.76 |
| **HLA-DRB1*08:01** | 386 | 400 | ANEADYQAKLTAYQT | 0.76 |
| **HLA-DRB1*13:01** | 1434 | 1448 | GAITIKFKEAFLRSV | 0.76 |
| **HLA-DRB1*08:01** | 1456 | 1470 | AESYIQMKRIAVGTF | 0.77 |
| **HLA-DRB1*03:01** | 624 | 638 | KIVYKYTVDPKSKFQ | 0.77 |
| **HLA-DRB1*01:01** | 962 | 975 | VEPTYEVIPTPPTD | 0.77 |
| **HLA-DRB1*08:01** | 198 | 212 | SKTAYEAKLAQYQAD | 0.78 |
| **HLA-DRB1*13:01** | 1432 | 1446 | TKGAITIKFKEAFLR | 0.78 |
| **HLA-DPA1*01:03/DPB1*04:02** | 988 | 1002 | PTVHFHYFKLAVQPQ | 0.78 |
| **HLA-DRB1*11:01** | 813 | 826 | NIWYSLNGKIRAVN | 0.79 |
| **HLA-DRB1*11:01** | 1457 | 1471 | ESYIQMKRIAVGTFE | 0.79 |
| **HLA-DPA1*01:03/DPB1*02:01** | 1449 | 1463 | SIDSAFQAESYIQMK | 0.79 |
| **HLA-DRB1*13:01** | 1258 | 1272 | KAGIRPKGAFQIFRA | 0.8 |
| **HLA-DRB1*15:01** | 228 | 241 | QKALAAYQAELKRV | 0.81 |
| **HLA-DRB1*04:01** | 1221 | 1234 | QDLVKITDANGNEV | 0.81 |
| **HLA-DRB1*15:01** | 471 | 485 | VKLKAYEDEQTSIKA | 0.81 |
| **HLA-DRB1*04:01** | 578 | 592 | DKAGWSTTVSNNSQV | 0.81 |
| **HLA-DRB1*04:01** | 862 | 876 | PNYEKEPTPPTRTPD | 0.81 |
| **HLA-DRB1*16:02** | 1239 | 1253 | VDNYTNLEAAPQEIR | 0.82 |
| **HLA-DRB1*04:01** | 1238 | 1251 | SVDNYTNLEAAPQE | 0.82 |
| **HLA-DRB1*13:01** | 1322 | 1335 | ASNIVINNVPKINP | 0.82 |
| **HLA-DRB1*08:01** | 288 | 301 | LAQYQAELKRVQEA | 0.82 |
| **HLA-DRB1*08:01** | 317 | 330 | QTELARVQKANADA | 0.82 |
| **HLA-DRB1*08:01** | 399 | 412 | QTELARVQKANADA | 0.82 |
| **HLA-DRB1*08:01** | 445 | 458 | KADYEAKLAKYQAD | 0.82 |
| **HLA-DRB1*03:01** | 643 | 656 | WLGIFTDPTLGVFA | 0.82 |
| **HLA-DRB1*11:01** | 315 | 329 | AYQTELARVQKANAD | 0.82 |
| **HLA-DRB1*11:01** | 397 | 411 | AYQTELARVQKANAD | 0.82 |
| **HLA-DRB1*01:01** | 1239 | 1252 | VDNYTNLEAAPQEI | 0.82 |
| **HLA-DPA1*02:01/DPB1*05:01** | 811 | 824 | KPNIWYSLNGKIRA | 0.83 |
| **HLA-DPA1*01:03/DPB1*02:01** | 1393 | 1406 | DHYTGQYKVFAKVD | 0.83 |
| **HLA-DRB1*04:01** | 961 | 974 | PVEPTYEVIPTPPT | 0.84 |
| **HLA-DRB1*04:01** | 1188 | 1201 | DLDQYKNDRSSADT | 0.84 |
| **HLA-DPA1*02:01/DPB1*05:01** | 374 | 387 | EADLAAVKKANAAN | 0.84 |
| **HLA-DRB1*01:01** | 185 | 199 | KAEVERINAANAASK | 0.84 |
| **HLA-DRB1*07:01** | 1467 | 1480 | VGTFENTYINTVNG | 0.85 |
| **HLA-DRB1*04:01** | 1240 | 1253 | DNYTNLEAAPQEIR | 0.85 |
| **HLA-DRB1*07:01** | 810 | 824 | KKPNIWYSLNGKIRA | 0.85 |
| **HLA-DPA1*02:01/DPB1*05:01** | 819 | 832 | NGKIRAVNVPKVTK | 0.85 |
| **HLA-DRB1*01:01** | 330 | 343 | AKATYEAAVAANNA | 0.85 |
| **HLA-DRB1*01:01** | 764 | 778 | PNSWYGAGAIKMSGP | 0.85 |
| **HLA-DRB1*15:01** | 818 | 831 | LNGKIRAVNVPKVT | 0.86 |
| **HLA-DRB1*07:01** | 1286 | 1299 | GIDLKIVSPMVVKK | 0.86 |
| **HLA-DRB1*15:01** | 648 | 662 | TDPTLGVFASAYTGQ | 0.86 |
| **HLA-DRB1*07:01** | 811 | 825 | KPNIWYSLNGKIRAV | 0.86 |
| **HLA-DRB1*11:01** | 1393 | 1406 | DHYTGQYKVFAKVD | 0.86 |
| **HLA-DRB1*01:01** | 412 | 425 | AKAAYEAAVAANNA | 0.86 |
| **HLA-DRB1*01:01** | 1042 | 1055 | TTSFVLVDPLPSGY | 0.86 |
| **HLA-DRB1*04:01** | 683 | 696 | KPINFDNALLSVTS | 0.87 |
| **HLA-DRB1*08:01** | 288 | 302 | LAQYQAELKRVQEAN | 0.87 |
| **HLA-DRB1*04:01** | 131 | 145 | ETEIKEDYTKQAEDI | 0.87 |
| **HLA-DRB1*08:01** | 281 | 294 | KAEYETKLAQYQAE | 0.88 |
| **HLA-DRB1*04:01** | 891 | 905 | EKPLEPAPVEPSYEA | 0.88 |
| **HLA-DRB1*04:01** | 930 | 944 | EKPLEPAPVEPSYEA | 0.88 |
| **HLA-DPA1*01:03/DPB1*02:01** | 614 | 628 | NSYYNGKKISKIVYK | 0.88 |
| **HLA-DRB1*16:02** | 1042 | 1056 | TTSFVLVDPLPSGYQ | 0.89 |
| **HLA-DRB1*16:02** | 331 | 345 | KATYEAAVAANNAKN | 0.89 |
| **HLA-DRB1*08:01** | 542 | 556 | KAKYDQKILQLDDLD | 0.89 |
| **HLA-DRB1*03:01** | 131 | 145 | ETEIKEDYTKQAEDI | 0.89 |
| **HLA-DPA1*02:01/DPB1*05:01** | 986 | 1000 | SDPTVHFHYFKLAVQ | 0.89 |
| **HLA-DPA1*01:03/DPB1*02:01** | 1393 | 1407 | DHYTGQYKVFAKVDI | 0.89 |
| **HLA-DRB1*04:01** | 819 | 833 | NGKIRAVNVPKVTKE | 0.9 |
| **HLA-DRB1*08:01** | 231 | 244 | LAAYQAELKRVQEA | 0.91 |
| **HLA-DPA1*02:01/DPB1*05:01** | 149 | 162 | TDQYKSDVAAHEAE | 0.91 |
| **HLA-DRB1*16:02** | 1042 | 1055 | TTSFVLVDPLPSGY | 0.92 |
| **HLA-DRB1*03:01** | 1012 | 1025 | DINIDRTLVAKQSV | 0.92 |
| **HLA-DRB1*07:01** | 1286 | 1300 | GIDLKIVSPMVVKKQ | 0.93 |
| **HLA-DRB1*03:01** | 323 | 337 | VQKANADAKATYEAA | 0.93 |
| **HLA-DRB1*16:02** | 900 | 914 | EPSYEAEPTPPTRTP | 0.94 |
| **HLA-DRB1*13:01** | 1258 | 1271 | KAGIRPKGAFQIFR | 0.94 |
| **HLA-DRB1*13:01** | 373 | 386 | YEADLAAVKKANAA | 0.94 |
| **HLA-DRB1*13:01** | 1323 | 1336 | SNIVINNVPKINPK | 0.94 |
| **HLA-DPA1*02:01/DPB1*05:01** | 1435 | 1448 | AITIKFKEAFLRSV | 0.94 |
| **HLA-DRB1*01:01** | 414 | 427 | AAYEAAVAANNAAN | 0.94 |
| **HLA-DRB1*03:01** | 405 | 418 | VQKANADAKAAYEA | 0.95 |
| **HLA-DRB1*03:01** | 1445 | 1458 | LRSVSIDSAFQAES | 0.95 |
| **HLA-DRB1*03:01** | 131 | 144 | ETEIKEDYTKQAED | 0.95 |
| **HLA-DRB1*03:01** | 1008 | 1021 | RNNNDINIDRTLVA | 0.95 |
| **HLA-DRB1*04:01** | 1052 | 1066 | PSGYQFNPEATKAAS | 0.95 |
| **HLA-DRB1*04:01** | 1188 | 1202 | DLDQYKNDRSSADTI | 0.95 |
| **HLA-DRB1*11:01** | 963 | 977 | EPTYEVIPTPPTDPV | 0.95 |
| **HLA-DPA1*01:03/DPB1*04:02** | 1449 | 1462 | SIDSAFQAESYIQM | 0.95 |
| **HLA-DRB1*16:02** | 1078 | 1091 | NTVTFKATAATLAT | 0.96 |
| **HLA-DRB1*15:01** | 1258 | 1272 | KAGIRPKGAFQIFRA | 0.96 |
| **HLA-DRB1*07:01** | 1466 | 1480 | AVGTFENTYINTVNG | 0.96 |
| **HLA-DRB1*07:01** | 1467 | 1481 | VGTFENTYINTVNGV | 0.96 |
| **HLA-DRB1*03:01** | 628 | 641 | KYTVDPKSKFQGQK | 0.96 |
| **HLA-DRB1*04:01** | 1381 | 1395 | EYNFYDDYDQTGDHY | 0.96 |
| **HLA-DRB1*04:01** | 1055 | 1069 | YQFNPEATKAASPGF | 0.96 |
| **HLA-DRB1*04:01** | 130 | 144 | KETEIKEDYTKQAED | 0.96 |
| **HLA-DPA1*01:03/DPB1*04:02** | 1462 | 1476 | MKRIAVGTFENTYIN | 0.96 |
| **HLA-DRB1*01:01** | 1240 | 1253 | DNYTNLEAAPQEIR | 0.96 |
| **HLA-DRB1*16:02** | 1078 | 1092 | NTVTFKATAATLATF | 0.97 |
| **HLA-DRB1*15:01** | 228 | 242 | QKALAAYQAELKRVQ | 0.97 |
| **HLA-DRB1*03:01** | 130 | 144 | KETEIKEDYTKQAED | 0.97 |
| **HLA-DPA1*02:01/DPB1*05:01** | 1449 | 1463 | SIDSAFQAESYIQMK | 0.97 |
| **HLA-DRB1*11:01** | 211 | 224 | ADLAAVQKTNAANQ | 0.98 |
| **HLA-DPA1*01:03/DPB1*02:01** | 984 | 998 | PPSDPTVHFHYFKLA | 0.98 |
| **HLA-DRB1*15:01** | 1258 | 1271 | KAGIRPKGAFQIFR | 0.99 |
| **HLA-DRB1*07:01** | 817 | 831 | SLNGKIRAVNVPKVT | 0.99 |
| **HLA-DRB1*08:01** | 444 | 458 | AKADYEAKLAKYQAD | 0.99 |
| **HLA-DRB1*08:01** | 1454 | 1468 | FQAESYIQMKRIAVG | 0.99 |
