# Supplementary material for: Design of a Novel Peptide‐Based Vaccine Targeting Streptococcus mutans SpaP Antigen for Dental Caries Prevention
Source: Int J Dent. 2026 Jun 29;2026:5545020. doi: 10.1155/ijod/5545020 (PMC13312148; doi:10.1155/ijod/5545020)
Supplement: Supplementary file 5 — Supporting Information 5 Data 5: A total of 100 possible multiepitope vaccine sequences. [file IJOD-2026-5545020-s005.docx]

**MHC-I (CTL):**

| **Allele** | **Start** | **End** | **Length** | **Peptide** |
| --- | --- | --- | --- | --- |
|  |  |  |  |  |
| HLA-A*24:02 | 41 | 49 | 9 | NYYELTWDL |
| HLA-B*08:01 | 29 | 37 | 9 | TVHFHYFKL |
| HLA-B*35:01 | 27 | 35 | 9 | DPTVHFHYF |
| HLA-A*24:02 | 49 | 57 | 9 | AYGIKSNVV |
| HLA-B*58:01 | 39 | 47 | 9 | STNYYELTW |
| HLA-A*24:02 | 37 | 46 | 10 | TYKNNFTLTV |

**MHC-II (HTL):**

| HLA-DRB1*08:01 | 361 | 375 | 15 | NAKATYEAALKQYEA |
| --- | --- | --- | --- | --- |
| HLA-DRB1*08:01 | 363 | 377 | 15 | KATYEAALKQYEADL |
| HLA-DRB1*11:01 | 363 | 376 | 14 | KATYEAALKQYEAD |
| HLA-DRB1*08:01 | 362 | 375 | 14 | AKATYEAALKQYEA |
| HLA-DRB1*08:01 | 364 | 377 | 14 | ATYEAALKQYEADL |
| HLA-DQA1*01:02/DQB1*06:02 | 477 | 490 | 14 | EDEQTSIKAALAEL |

**BCE:**

| **start** | **end** | **Epitope** |
| --- | --- | --- |
| 949 | 955 | PTPTPDQ |
| 870 | 876 | PPTRTPD |
| 948 | 954 | PPTPTPD |
| 871 | 877 | PTRTPDQ |
| 872 | 878 | TRTPDQA |
| 875 | 881 | PDQAEPN |

**AdjuvantEAAAKPADREPMGLPCTLAAYCTLAAYCTLAAYCTLAAYCTLAAYCTLAAYHTLGPGPGHTLGPGPGHTLGPGPGHTLGPGPGHTLGPGPGHTLGPGPGBCEKKBCEKKBCEKKBCEKKBCEKKBCEKKHisTag**

**Adjuvant (130 Aa): (1, 2)**

**MAKLSTDELLDAFKEMTLLELSDFVKKFEETFEVTAAAPVAVAAAGAAPAGAAVEAAEEQSEFDVILEAAGDKKIGVIKVVREIVSGLGLKEAKDLVDGAPKPLLEKVAKEAADEAKAKLEAAGATVTVK**

**PADRE: (13 Aa)**

**AKFVAAWTLKAAA**

1. MAKLSTDELLDAFKEMTLLELSDFVKKFEETFEVTAAAPVAVAAAGAAPAGAAVEAAEEQSEFDVILEAAGDKKIGVIKVVREIVSGLGLKEAKDLVDGAPKPLLEKVAKEAADEAKAKLEAAGATVTVKEAAAKAKFVAAWTLKAAAPMGLPNYYELTWDLAAYTVHFHYFKLAAYDPTVHFHYFAAYAYGIKSNVVAAYSTNYYELTWAAYTYKNNFTLTVAAYNAKATYEAALKQYEAGPGPGKATYEAALKQYEADLGPGPGKATYEAALKQYEADGPGPGAKATYEAALKQYEAGPGPGATYEAALKQYEADLGPGPGEDEQTSIKAALAELGPGPGPTPTPDQKKPPTRTPDKKPPTPTPDKKPTRTPDQKKTRTPDQAKKPDQAEPNKKHHHHHH

2. MAKLSTDELLDAFKEMTLLELSDFVKKFEETFEVTAAAPVAVAAAGAAPAGAAVEAAEEQSEFDVILEAAGDKKIGVIKVVREIVSGLGLKEAKDLVDGAPKPLLEKVAKEAADEAKAKLEAAGATVTVKEAAAKAKFVAAWTLKAAA**PMGLP**NYYELTWDL**AAY**DPTVHFHYF**AAY**AYGIKSNVV**AAY**STNYYELTW**AAY**TYKNNFTLTV**AAY**TVHFHYFKL**AAY**NAKATYEAALKQYEAGPGPGKATYEAALKQYEADLGPGPGKATYEAALKQYEADGPGPGAKATYEAALKQYEAGPGPGATYEAALKQYEADLGPGPGEDEQTSIKAALAELGPGPGPTPTPDQKKPPTRTPDKKPPTPTPDKKPTRTPDQKKTRTPDQAKKPDQAEPNKKHHHHHH

3. MAKLSTDELLDAFKEMTLLELSDFVKKFEETFEVTAAAPVAVAAAGAAPAGAAVEAAEEQSEFDVILEAAGDKKIGVIKVVREIVSGLGLKEAKDLVDGAPKPLLEKVAKEAADEAKAKLEAAGATVTVKEAAAKAKFVAAWTLKAAA**PMGLP**NYYELTWDL**AAY**AYGIKSNVV**AAY**STNYYELTW**AAY**TYKNNFTLTV**AAY**TVHFHYFKL**AAY**DPTVHFHYF**AAY**NAKATYEAALKQYEAGPGPGKATYEAALKQYEADLGPGPGKATYEAALKQYEADGPGPGAKATYEAALKQYEAGPGPGATYEAALKQYEADLGPGPGEDEQTSIKAALAELGPGPGPTPTPDQKKPPTRTPDKKPPTPTPDKK PTRTPDQKKTRTPDQAKKPDQAEPNKKHHHHHH

**4.**

MAKLSTDELLDAFKEMTLLELSDFVKKFEETFEVTAAAPVAVAAAGAAPAGAAVEAAEEQSEFDVILEAAGDKKIGVIKVVREIVSGLGLKEAKDLVDGAPKPLLEKVAKEAADEAKAKLEAAGATVTVKEAAAKAKFVAAWTLKAAA**PMGLP**NYYELTWDL**AAY**AYGIKSNVV**AAY**STNYYELTW**AAY**TYKNNFTLTV**AAY**DPTVHFHYF**AAY**TVHFHYFKL**AAY**NAKATYEAALKQYEAGPGPGKATYEAALKQYEADLGPGPGKATYEAALKQYEADGPGPGAKATYEAALKQYEAGPGPGATYEAALKQYEADLGPGPGEDEQTSIKAALAELGPGPGPTPTPDQKKPPTRTPDKKPPTPTPDKKPTRTPDQKKTRTPDQAKKPDQAEPNKKHHHHHH

**5.**

MAKLSTDELLDAFKEMTLLELSDFVKKFEETFEVTAAAPVAVAAAGAAPAGAAVEAAEEQSEFDVILEAAGDKKIGVIKVVREIVSGLGLKEAKDLVDGAPKPLLEKVAKEAADEAKAKLEAAGATVTVKEAAAKAKFVAAWTLKAAA**PMGLP**NYYELTWDL**AAY**STNYYELTW**AAY**TYKNNFTLTV**AAY**TVHFHYFKL**AAY**DPTVHFHYF**AAY**AYGIKSNVV**AAY**NAKATYEAALKQYEAGPGPGKATYEAALKQYEADLGPGPGKATYEAALKQYEADGPGPGAKATYEAALKQYEAGPGPGATYEAALKQYEADLGPGPGEDEQTSIKAALAELGPGPGPTPTPDQKKPPTRTPDKKPPTPTPDKKPTRTPDQKKTRTPDQAKKPDQAEPNKKHHHHHH

**6.**

MAKLSTDELLDAFKEMTLLELSDFVKKFEETFEVTAAAPVAVAAAGAAPAGAAVEAAEEQSEFDVILEAAGDKKIGVIKVVREIVSGLGLKEAKDLVDGAPKPLLEKVAKEAADEAKAKLEAAGATVTVKEAAAKAKFVAAWTLKAAA**PMGLP**NYYELTWDL**AAY**STNYYELTW**AAY**TYKNNFTLTV**AAY**TVHFHYFKL**AAY**AYGIKSNVV**AAY**DPTVHFHYF**AAY**NAKATYEAALKQYEAGPGPGKATYEAALKQYEADLGPGPGKATYEAALKQYEADGPGPGAKATYEAALKQYEAGPGPGATYEAALKQYEADLGPGPGEDEQTSIKAALAELGPGPGPTPTPDQKKPPTRTPDKKPPTPTPDKKPTRTPDQKKTRTPDQAKKPDQAEPNKKHHHHHH

**7.**

MAKLSTDELLDAFKEMTLLELSDFVKKFEETFEVTAAAPVAVAAAGAAPAGAAVEAAEEQSEFDVILEAAGDKKIGVIKVVREIVSGLGLKEAKDLVDGAPKPLLEKVAKEAADEAKAKLEAAGATVTVKEAAAKAKFVAAWTLKAAA**PMGLP**NYYELTWDL**AAY**STNYYELTW**AAY**TYKNNFTLTV**AAY**AYGIKSNVV**AAY**DPTVHFHYF**AAY**TVHFHYFKL**AAY**NAKATYEAALKQYEAGPGPGKATYEAALKQYEADLGPGPGKATYEAALKQYEADGPGPGAKATYEAALKQYEAGPGPGATYEAALKQYEADLGPGPGEDEQTSIKAALAELGPGPGPTPTPDQKKPPTRTPDKKPPTPTPDKKPTRTPDQKKTRTPDQAKKPDQAEPNKKHHHHHH

**8.**

MAKLSTDELLDAFKEMTLLELSDFVKKFEETFEVTAAAPVAVAAAGAAPAGAAVEAAEEQSEFDVILEAAGDKKIGVIKVVREIVSGLGLKEAKDLVDGAPKPLLEKVAKEAADEAKAKLEAAGATVTVKEAAAKAKFVAAWTLKAAA**PMGLP**NYYELTWDL**AAY**STNYYELTW**AAY**TYKNNFTLTV**AAY**AYGIKSNVV**AAY**TVHFHYFKL**AAY**DPTVHFHYF**AAY**NAKATYEAALKQYEAGPGPGKATYEAALKQYEADLGPGPGKATYEAALKQYEADGPGPGAKATYEAALKQYEAGPGPGATYEAALKQYEADLGPGPGEDEQTSIKAALAELGPGPGPTPTPDQKKPPTRTPDKKPPTPTPDKKPTRTPDQKKTRTPDQAKKPDQAEPNKKHHHHHH

**9.**

MAKLSTDELLDAFKEMTLLELSDFVKKFEETFEVTAAAPVAVAAAGAAPAGAAVEAAEEQSEFDVILEAAGDKKIGVIKVVREIVSGLGLKEAKDLVDGAPKPLLEKVAKEAADEAKAKLEAAGATVTVKEAAAKAKFVAAWTLKAAA**PMGLP**NYYELTWDL**AAY**TYKNNFTLTV**AAY**TVHFHYFKL**AAY**DPTVHFHYF**AAY**AYGIKSNVV**AAY**STNYYELTW**AAY**NAKATYEAALKQYEAGPGPGKATYEAALKQYEADLGPGPGKATYEAALKQYEADGPGPGAKATYEAALKQYEAGPGPGATYEAALKQYEADLGPGPGEDEQTSIKAALAELGPGPGPTPTPDQKKPPTRTPDKKPPTPTPDKKPTRTPDQKKTRTPDQAKKPDQAEPNKKHHHHHH

**10.**

MAKLSTDELLDAFKEMTLLELSDFVKKFEETFEVTAAAPVAVAAAGAAPAGAAVEAAEEQSEFDVILEAAGDKKIGVIKVVREIVSGLGLKEAKDLVDGAPKPLLEKVAKEAADEAKAKLEAAGATVTVKEAAAKAKFVAAWTLKAAA**PMGLP**NYYELTWDL**AAY**TYKNNFTLTV**AAY**DPTVHFHYF**AAY**AYGIKSNVV**AAY**STNYYELTW**AAY**TVHFHYFKL**AAY**NAKATYEAALKQYEAGPGPGKATYEAALKQYEADLGPGPGKATYEAALKQYEADGPGPGAKATYEAALKQYEAGPGPGATYEAALKQYEADLGPGPGEDEQTSIKAALAELGPGPGPTPTPDQKKPPTRTPDKKPPTPTPDKKPTRTPDQKKTRTPDQAKKPDQAEPNKKHHHHHH

**11.**

MAKLSTDELLDAFKEMTLLELSDFVKKFEETFEVTAAAPVAVAAAGAAPAGAAVEAAEEQSEFDVILEAAGDKKIGVIKVVREIVSGLGLKEAKDLVDGAPKPLLEKVAKEAADEAKAKLEAAGATVTVKEAAAKAKFVAAWTLKAAA**PMGLP**NYYELTWDL**AAY**TYKNNFTLTV**AAY**AYGIKSNVV**AAY**STNYYELTW**AAY**TVHFHYFKL**AAY**DPTVHFHYF**AAY**NAKATYEAALKQYEAGPGPGKATYEAALKQYEADLGPGPGKATYEAALKQYEADGPGPGAKATYEAALKQYEAGPGPGATYEAALKQYEADLGPGPGEDEQTSIKAALAELGPGPGPTPTPDQKKPPTRTPDKKPPTPTPDKKPTRTPDQKKTRTPDQAKKPDQAEPNKKHHHHHH

**12.**

MAKLSTDELLDAFKEMTLLELSDFVKKFEETFEVTAAAPVAVAAAGAAPAGAAVEAAEEQSEFDVILEAAGDKKIGVIKVVREIVSGLGLKEAKDLVDGAPKPLLEKVAKEAADEAKAKLEAAGATVTVKEAAAKAKFVAAWTLKAAA**PMGLP**NYYELTWDL**AAY**TYKNNFTLTV**AAY**STNYYELTW**AAY**TVHFHYFKL**AAY**DPTVHFHYF**AAY**AYGIKSNVV**AAY**NAKATYEAALKQYEAGPGPGKATYEAALKQYEADLGPGPGKATYEAALKQYEADGPGPGAKATYEAALKQYEAGPGPGATYEAALKQYEADLGPGPGEDEQTSIKAALAELGPGPGPTPTPDQKKPPTRTPDKKPPTPTPDKKPTRTPDQKKTRTPDQAKKPDQAEPNKKHHHHHH

**13.**

MAKLSTDELLDAFKEMTLLELSDFVKKFEETFEVTAAAPVAVAAAGAAPAGAAVEAAEEQSEFDVILEAAGDKKIGVIKVVREIVSGLGLKEAKDLVDGAPKPLLEKVAKEAADEAKAKLEAAGATVTVKEAAAKAKFVAAWTLKAAA**PMGLP**NYYELTWDL**AAY**TYKNNFTLTV**AAY**STNYYELTW**AAY**AYGIKSNVV**AAY**DPTVHFHYF**AAY**TVHFHYFKL**AAY**NAKATYEAALKQYEAGPGPGKATYEAALKQYEADLGPGPGKATYEAALKQYEADGPGPGAKATYEAALKQYEAGPGPGATYEAALKQYEADLGPGPGEDEQTSIKAALAELGPGPGPTPTPDQKKPPTRTPDKKPPTPTPDKKPTRTPDQKKTRTPDQAKKPDQAEPNKKHHHHHH

**14.**

MAKLSTDELLDAFKEMTLLELSDFVKKFEETFEVTAAAPVAVAAAGAAPAGAAVEAAEEQSEFDVILEAAGDKKIGVIKVVREIVSGLGLKEAKDLVDGAPKPLLEKVAKEAADEAKAKLEAAGATVTVKEAAAKAKFVAAWTLKAAA**PMGLP**NYYELTWDL**AAY**TYKNNFTLTV**AAY**STNYYELTW**AAY**DPTVHFHYF**AAY**AYGIKSNVV**AAY**TVHFHYFKL**AAY**NAKATYEAALKQYEAGPGPGKATYEAALKQYEADLGPGPGKATYEAALKQYEADGPGPGAKATYEAALKQYEAGPGPGATYEAALKQYEADLGPGPGEDEQTSIKAALAELGPGPGPTPTPDQKKPPTRTPDKKPPTPTPDKKPTRTPDQKKTRTPDQAKKPDQAEPNKKHHHHHH

**15.**

MAKLSTDELLDAFKEMTLLELSDFVKKFEETFEVTAAAPVAVAAAGAAPAGAAVEAAEEQSEFDVILEAAGDKKIGVIKVVREIVSGLGLKEAKDLVDGAPKPLLEKVAKEAADEAKAKLEAAGATVTVKEAAAKAKFVAAWTLKAAA**PMGLP**NYYELTWDL**AAY**TYKNNFTLTV**AAY**STNYYELTW**AAY**TVHFHYFKL**AAY**AYGIKSNVV**AAY**DPTVHFHYF**AAY**NAKATYEAALKQYEAGPGPGKATYEAALKQYEADLGPGPGKATYEAALKQYEADGPGPGAKATYEAALKQYEAGPGPGATYEAALKQYEADLGPGPGEDEQTSIKAALAELGPGPGPTPTPDQKKPPTRTPDKKPPTPTPDKKPTRTPDQKKTRTPDQAKKPDQAEPNKKHHHHHH

**16.**

MAKLSTDELLDAFKEMTLLELSDFVKKFEETFEVTAAAPVAVAAAGAAPAGAAVEAAEEQSEFDVILEAAGDKKIGVIKVVREIVSGLGLKEAKDLVDGAPKPLLEKVAKEAADEAKAKLEAAGATVTVKEAAAKAKFVAAWTLKAAA**PMGLP**NYYELTWDL**AAY**TYKNNFTLTV**AAY**AYGIKSNVV**AAY**DPTVHFHYF**AAY**TVHFHYFKL**AAY**STNYYELTW**AAY**NAKATYEAALKQYEAGPGPGKATYEAALKQYEADLGPGPGKATYEAALKQYEADGPGPGAKATYEAALKQYEAGPGPGATYEAALKQYEADLGPGPGEDEQTSIKAALAELGPGPGPTPTPDQKKPPTRTPDKKPPTPTPDKKPTRTPDQKKTRTPDQAKKPDQAEPNKKHHHHHH

**17.**

MAKLSTDELLDAFKEMTLLELSDFVKKFEETFEVTAAAPVAVAAAGAAPAGAAVEAAEEQSEFDVILEAAGDKKIGVIKVVREIVSGLGLKEAKDLVDGAPKPLLEKVAKEAADEAKAKLEAAGATVTVKEAAAKAKFVAAWTLKAAA**PMGLP**NYYELTWDL**AAY**TYKNNFTLTV**AAY**AYGIKSNVV**AAY**STNYYELTW**AAY**DPTVHFHYF**AAY**TVHFHYFKL**AAY**NAKATYEAALKQYEAGPGPGKATYEAALKQYEADLGPGPGKATYEAALKQYEADGPGPGAKATYEAALKQYEAGPGPGATYEAALKQYEADLGPGPGEDEQTSIKAALAELGPGPGPTPTPDQKKPPTRTPDKKPPTPTPDKKPTRTPDQKKTRTPDQAKKPDQAEPNKKHHHHHH

**18.**MAKLSTDELLDAFKEMTLLELSDFVKKFEETFEVTAAAPVAVAAAGAAPAGAAVEAAEEQSEFDVILEAAGDKKIGVIKVVREIVSGLGLKEAKDLVDGAPKPLLEKVAKEAADEAKAKLEAAGATVTVKEAAAKAKFVAAWTLKAAA**PMGLP**NYYELTWDL**AAY**TYKNNFTLTV**AAY**AYGIKSNVV**AAY**TVHFHYFKL**AAY**DPTVHFHYF**AAY**STNYYELTW**AAY**NAKATYEAALKQYEAGPGPGKATYEAALKQYEADLGPGPGKATYEAALKQYEADGPGPGAKATYEAALKQYEAGPGPGATYEAALKQYEADLGPGPGEDEQTSIKAALAELGPGPGPTPTPDQKKPPTRTPDKKPPTPTPDKKPTRTPDQKKTRTPDQAKKPDQAEPNKKHHHHHH

**19.**

MAKLSTDELLDAFKEMTLLELSDFVKKFEETFEVTAAAPVAVAAAGAAPAGAAVEAAEEQSEFDVILEAAGDKKIGVIKVVREIVSGLGLKEAKDLVDGAPKPLLEKVAKEAADEAKAKLEAAGATVTVKEAAAKAKFVAAWTLKAAA**PMGLP**NYYELTWDL**AAY**TYKNNFTLTV**AAY**AYGIKSNVV**AAY**DPTVHFHYF**AAY**STNYYELTW**AAY**TVHFHYFKL**AAY**NAKATYEAALKQYEAGPGPGKATYEAALKQYEADLGPGPGKATYEAALKQYEADGPGPGAKATYEAALKQYEAGPGPGATYEAALKQYEADLGPGPGEDEQTSIKAALAELGPGPGPTPTPDQKKPPTRTPDKKPPTPTPDKKPTRTPDQKKTRTPDQAKKPDQAEPNKKHHHHHH

**20.**

MAKLSTDELLDAFKEMTLLELSDFVKKFEETFEVTAAAPVAVAAAGAAPAGAAVEAAEEQSEFDVILEAAGDKKIGVIKVVREIVSGLGLKEAKDLVDGAPKPLLEKVAKEAADEAKAKLEAAGATVTVKEAAAKAKFVAAWTLKAAA**PMGLP**TVHFHYFKL**AAY**DPTVHFHYF**AAY**AYGIKSNVV**AAY**STNYYELTW**AAY**TYKNNFTLTV**AAY**NYYELTWDL**AAY**NAKATYEAALKQYEAGPGPGKATYEAALKQYEADLGPGPGKATYEAALKQYEADGPGPGAKATYEAALKQYEAGPGPGATYEAALKQYEADLGPGPGEDEQTSIKAALAELGPGPGPTPTPDQKKPPTRTPDKKPPTPTPDKKPTRTPDQKKTRTPDQAKKPDQAEPNKKHHHHHH

**21.**

MAKLSTDELLDAFKEMTLLELSDFVKKFEETFEVTAAAPVAVAAAGAAPAGAAVEAAEEQSEFDVILEAAGDKKIGVIKVVREIVSGLGLKEAKDLVDGAPKPLLEKVAKEAADEAKAKLEAAGATVTVKEAAAKAKFVAAWTLKAAA**PMGLP**DPTVHFHYF**AAY**AYGIKSNVV**AAY**STNYYELTW**AAY**TYKNNFTLTV**AAY**NYYELTWDL**AAY**TVHFHYFKL**AAY**NAKATYEAALKQYEAGPGPGKATYEAALKQYEADLGPGPGKATYEAALKQYEADGPGPGAKATYEAALKQYEAGPGPGATYEAALKQYEADLGPGPGEDEQTSIKAALAELGPGPGPTPTPDQKKPPTRTPDKKPPTPTPDKKPTRTPDQKKTRTPDQAKKPDQAEPNKKHHHHHH

**22.**

MAKLSTDELLDAFKEMTLLELSDFVKKFEETFEVTAAAPVAVAAAGAAPAGAAVEAAEEQSEFDVILEAAGDKKIGVIKVVREIVSGLGLKEAKDLVDGAPKPLLEKVAKEAADEAKAKLEAAGATVTVKEAAAKAKFVAAWTLKAAA**PMGLP**DPTVHFHYF**AAY**AYGIKSNVV**AAY**STNYYELTW**AAY**TYKNNFTLTV**AAY**TVHFHYFKL**AAY**NYYELTWDL**AAY**NAKATYEAALKQYEAGPGPGKATYEAALKQYEADLGPGPGKATYEAALKQYEADGPGPGAKATYEAALKQYEAGPGPGATYEAALKQYEADLGPGPGEDEQTSIKAALAELGPGPGPTPTPDQKKPPTRTPDKKPPTPTPDKKPTRTPDQKKTRTPDQAKKPDQAEPNKKHHHHHH

**23.**

MAKLSTDELLDAFKEMTLLELSDFVKKFEETFEVTAAAPVAVAAAGAAPAGAAVEAAEEQSEFDVILEAAGDKKIGVIKVVREIVSGLGLKEAKDLVDGAPKPLLEKVAKEAADEAKAKLEAAGATVTVKEAAAKAKFVAAWTLKAAA**PMGLP**AYGIKSNVV**AAY**STNYYELTW**AAY**TYKNNFTLTV**AAY**NYYELTWDL**AAY**TVHFHYFKL**AAY**DPTVHFHYF**AAY**NAKATYEAALKQYEAGPGPGKATYEAALKQYEADLGPGPGKATYEAALKQYEADGPGPGAKATYEAALKQYEAGPGPGATYEAALKQYEADLGPGPGEDEQTSIKAALAELGPGPGPTPTPDQKKPPTRTPDKKPPTPTPDKKPTRTPDQKKTRTPDQAKKPDQAEPNKKHHHHHH

**24.**

MAKLSTDELLDAFKEMTLLELSDFVKKFEETFEVTAAAPVAVAAAGAAPAGAAVEAAEEQSEFDVILEAAGDKKIGVIKVVREIVSGLGLKEAKDLVDGAPKPLLEKVAKEAADEAKAKLEAAGATVTVKEAAAKAKFVAAWTLKAAA**PMGLP**AYGIKSNVV**AAY**STNYYELTW**AAY**TYKNNFTLTV**AAY**TVHFHYFKL**AAY**DPTVHFHYF**AAY**NYYELTWDL**AAY**NAKATYEAALKQYEAGPGPGKATYEAALKQYEADLGPGPGKATYEAALKQYEADGPGPGAKATYEAALKQYEAGPGPGATYEAALKQYEADLGPGPGEDEQTSIKAALAELGPGPGPTPTPDQKKPPTRTPDKKPPTPTPDKKPTRTPDQKKTRTPDQAKKPDQAEPNKKHHHHHH

**25.**

MAKLSTDELLDAFKEMTLLELSDFVKKFEETFEVTAAAPVAVAAAGAAPAGAAVEAAEEQSEFDVILEAAGDKKIGVIKVVREIVSGLGLKEAKDLVDGAPKPLLEKVAKEAADEAKAKLEAAGATVTVKEAAAKAKFVAAWTLKAAA**PMGLP**AYGIKSNVV**AAY**STNYYELTW**AAY**TYKNNFTLTV**AAY**DPTVHFHYF**AAY**TVHFHYFKL**AAY**NYYELTWDL**AAY**NAKATYEAALKQYEAGPGPGKATYEAALKQYEADLGPGPGKATYEAALKQYEADGPGPGAKATYEAALKQYEAGPGPGATYEAALKQYEADLGPGPGEDEQTSIKAALAELGPGPGPTPTPDQKKPPTRTPDKKPPTPTPDKKPTRTPDQKKTRTPDQAKKPDQAEPNKKHHHHHH

**26.**

MAKLSTDELLDAFKEMTLLELSDFVKKFEETFEVTAAAPVAVAAAGAAPAGAAVEAAEEQSEFDVILEAAGDKKIGVIKVVREIVSGLGLKEAKDLVDGAPKPLLEKVAKEAADEAKAKLEAAGATVTVKEAAAKAKFVAAWTLKAAA**PMGLP**AYGIKSNVV**AAY**STNYYELTW**AAY**TYKNNFTLTV**AAY**DPTVHFHYF**AAY**NYYELTWDL**AAY**TVHFHYFKL**AAY**NAKATYEAALKQYEAGPGPGKATYEAALKQYEADLGPGPGKATYEAALKQYEADGPGPGAKATYEAALKQYEAGPGPGATYEAALKQYEADLGPGPGEDEQTSIKAALAELGPGPGPTPTPDQKKPPTRTPDKKPPTPTPDKKPTRTPDQKKTRTPDQAKKPDQAEPNKKHHHHHH

**27.** MAKLSTDELLDAFKEMTLLELSDFVKKFEETFEVTAAAPVAVAAAGAAPAGAAVEAAEEQSEFDVILEAAGDKKIGVIKVVREIVSGLGLKEAKDLVDGAPKPLLEKVAKEAADEAKAKLEAAGATVTVKEAAAKAKFVAAWTLKAAA**PMGLP**AYGIKSNVV**AAY**STNYYELTW**AAY**TYKNNFTLTV**AAY**TVHFHYFKL**AAY**NYYELTWDL**AAY**DPTVHFHYF**AAY**NAKATYEAALKQYEAGPGPGKATYEAALKQYEADLGPGPGKATYEAALKQYEADGPGPGAKATYEAALKQYEAGPGPGATYEAALKQYEADLGPGPGEDEQTSIKAALAELGPGPGPTPTPDQKKPPTRTPDKKPPTPTPDKKPTRTPDQKKTRTPDQAKKPDQAEPNKKHHHHHH

**28.**

MAKLSTDELLDAFKEMTLLELSDFVKKFEETFEVTAAAPVAVAAAGAAPAGAAVEAAEEQSEFDVILEAAGDKKIGVIKVVREIVSGLGLKEAKDLVDGAPKPLLEKVAKEAADEAKAKLEAAGATVTVKEAAAKAKFVAAWTLKAAA**PMGLP**STNYYELTW**AAY**TYKNNFTLTV**AAY**NYYELTWDL**AAY**TVHFHYFKL**AAY**DPTVHFHYF**AAY**AYGIKSNVV**AAY**NAKATYEAALKQYEAGPGPGKATYEAALKQYEADLGPGPGKATYEAALKQYEADGPGPGAKATYEAALKQYEAGPGPGATYEAALKQYEADLGPGPGEDEQTSIKAALAELGPGPGPTPTPDQKKPPTRTPDKKPPTPTPDKKPTRTPDQKKTRTPDQAKKPDQAEPNKKHHHHHH

**29.**

MAKLSTDELLDAFKEMTLLELSDFVKKFEETFEVTAAAPVAVAAAGAAPAGAAVEAAEEQSEFDVILEAAGDKKIGVIKVVREIVSGLGLKEAKDLVDGAPKPLLEKVAKEAADEAKAKLEAAGATVTVKEAAAKAKFVAAWTLKAAA**PMGLP**STNYYELTW**AAY**TYKNNFTLTV**AAY**TVHFHYFKL**AAY**DPTVHFHYF**AAY**AYGIKSNVV**AAY**NYYELTWDL**AAY**NAKATYEAALKQYEAGPGPGKATYEAALKQYEADLGPGPGKATYEAALKQYEADGPGPGAKATYEAALKQYEAGPGPGATYEAALKQYEADLGPGPGEDEQTSIKAALAELGPGPGPTPTPDQKKPPTRTPDKKPPTPTPDKKPTRTPDQKKTRTPDQAKKPDQAEPNKKHHHHHH

**30.**

MAKLSTDELLDAFKEMTLLELSDFVKKFEETFEVTAAAPVAVAAAGAAPAGAAVEAAEEQSEFDVILEAAGDKKIGVIKVVREIVSGLGLKEAKDLVDGAPKPLLEKVAKEAADEAKAKLEAAGATVTVKEAAAKAKFVAAWTLKAAA**PMGLP**STNYYELTW**AAY**TYKNNFTLTV**AAY**DPTVHFHYF**AAY**AYGIKSNVV**AAY**NYYELTWDL**AAY**TVHFHYFKL**AAY**NAKATYEAALKQYEAGPGPGKATYEAALKQYEADLGPGPGKATYEAALKQYEADGPGPGAKATYEAALKQYEAGPGPGATYEAALKQYEADLGPGPGEDEQTSIKAALAELGPGPGPTPTPDQKKPPTRTPDKKPPTPTPDKKPTRTPDQKKTRTPDQAKKPDQAEPNKKHHHHHH

**31.**

MAKLSTDELLDAFKEMTLLELSDFVKKFEETFEVTAAAPVAVAAAGAAPAGAAVEAAEEQSEFDVILEAAGDKKIGVIKVVREIVSGLGLKEAKDLVDGAPKPLLEKVAKEAADEAKAKLEAAGATVTVKEAAAKAKFVAAWTLKAAA**PMGLP**STNYYELTW**AAY**TYKNNFTLTV**AAY**AYGIKSNVV**AAY**NYYELTWDL**AAY**TVHFHYFKL**AAY**DPTVHFHYF**AAY**NAKATYEAALKQYEAGPGPGKATYEAALKQYEADLGPGPGKATYEAALKQYEADGPGPGAKATYEAALKQYEAGPGPGATYEAALKQYEADLGPGPGEDEQTSIKAALAELGPGPGPTPTPDQKKPPTRTPDKKPPTPTPDKKPTRTPDQKKTRTPDQAKKPDQAEPNKKHHHHHH

**32.**

MAKLSTDELLDAFKEMTLLELSDFVKKFEETFEVTAAAPVAVAAAGAAPAGAAVEAAEEQSEFDVILEAAGDKKIGVIKVVREIVSGLGLKEAKDLVDGAPKPLLEKVAKEAADEAKAKLEAAGATVTVKEAAAKAKFVAAWTLKAAA**PMGLP**STNYYELTW**AAY**TYKNNFTLTV**AAY**TVHFHYFKL**AAY**NYYELTWDL**AAY**DPTVHFHYF**AAY**AYGIKSNVV**AAY**NAKATYEAALKQYEAGPGPGKATYEAALKQYEADLGPGPGKATYEAALKQYEADGPGPGAKATYEAALKQYEAGPGPGATYEAALKQYEADLGPGPGEDEQTSIKAALAELGPGPGPTPTPDQKKPPTRTPDKKPPTPTPDKKPTRTPDQKKTRTPDQAKKPDQAEPNKKHHHHHH

**33.**

MAKLSTDELLDAFKEMTLLELSDFVKKFEETFEVTAAAPVAVAAAGAAPAGAAVEAAEEQSEFDVILEAAGDKKIGVIKVVREIVSGLGLKEAKDLVDGAPKPLLEKVAKEAADEAKAKLEAAGATVTVKEAAAKAKFVAAWTLKAAA**PMGLP**STNYYELTW**AAY**TYKNNFTLTV**AAY**TVHFHYFKL**AAY**DPTVHFHYF**AAY**NYYELTWDL**AAY**AYGIKSNVV**AAY**NAKATYEAALKQYEAGPGPGKATYEAALKQYEADLGPGPGKATYEAALKQYEADGPGPGAKATYEAALKQYEAGPGPGATYEAALKQYEADLGPGPGEDEQTSIKAALAELGPGPGPTPTPDQKKPPTRTPDKKPPTPTPDKKPTRTPDQKKTRTPDQAKKPDQAEPNKKHHHHHH

**34.**

MAKLSTDELLDAFKEMTLLELSDFVKKFEETFEVTAAAPVAVAAAGAAPAGAAVEAAEEQSEFDVILEAAGDKKIGVIKVVREIVSGLGLKEAKDLVDGAPKPLLEKVAKEAADEAKAKLEAAGATVTVKEAAAKAKFVAAWTLKAAA**PMGLP**STNYYELTW**AAY**TYKNNFTLTV**AAY**TVHFHYFKL**AAY**AYGIKSNVV**AAY**NYYELTWDL**AAY**DPTVHFHYF**AAY**NAKATYEAALKQYEAGPGPGKATYEAALKQYEADLGPGPGKATYEAALKQYEADGPGPGAKATYEAALKQYEAGPGPGATYEAALKQYEADLGPGPGEDEQTSIKAALAELGPGPGPTPTPDQKKPPTRTPDKKPPTPTPDKKPTRTPDQKKTRTPDQAKKPDQAEPNKKHHHHHH

**35.**

MAKLSTDELLDAFKEMTLLELSDFVKKFEETFEVTAAAPVAVAAAGAAPAGAAVEAAEEQSEFDVILEAAGDKKIGVIKVVREIVSGLGLKEAKDLVDGAPKPLLEKVAKEAADEAKAKLEAAGATVTVKEAAAKAKFVAAWTLKAAA**PMGLP**STNYYELTW**AAY**TYKNNFTLTV**AAY**TVHFHYFKL**AAY**AYGIKSNVV**AAY**DPTVHFHYF**AAY**NYYELTWDL**AAY**NAKATYEAALKQYEAGPGPGKATYEAALKQYEADLGPGPGKATYEAALKQYEADGPGPGAKATYEAALKQYEAGPGPGATYEAALKQYEADLGPGPGEDEQTSIKAALAELGPGPGPTPTPDQKKPPTRTPDKKPPTPTPDKKPTRTPDQKKTRTPDQAKKPDQAEPNKKHHHHHH

**36.**

MAKLSTDELLDAFKEMTLLELSDFVKKFEETFEVTAAAPVAVAAAGAAPAGAAVEAAEEQSEFDVILEAAGDKKIGVIKVVREIVSGLGLKEAKDLVDGAPKPLLEKVAKEAADEAKAKLEAAGATVTVKEAAAKAKFVAAWTLKAAA**PMGLP**STNYYELTW**AAY**TYKNNFTLTV**AAY**DPTVHFHYF**AAY**TVHFHYFKL**AAY**NYYELTWDL**AAY**AYGIKSNVV**AAY**NAKATYEAALKQYEAGPGPGKATYEAALKQYEADLGPGPGKATYEAALKQYEADGPGPGAKATYEAALKQYEAGPGPGATYEAALKQYEADLGPGPGEDEQTSIKAALAELGPGPGPTPTPDQKKPPTRTPDKKPPTPTPDKKPTRTPDQKKTRTPDQAKKPDQAEPNKKHHHHHH

**37.**

MAKLSTDELLDAFKEMTLLELSDFVKKFEETFEVTAAAPVAVAAAGAAPAGAAVEAAEEQSEFDVILEAAGDKKIGVIKVVREIVSGLGLKEAKDLVDGAPKPLLEKVAKEAADEAKAKLEAAGATVTVKEAAAKAKFVAAWTLKAAA**PMGLP**STNYYELTW**AAY**TYKNNFTLTV**AAY**DPTVHFHYF**AAY**AYGIKSNVV**AAY**TVHFHYFKL**AAY**NYYELTWDL**AAY**NAKATYEAALKQYEAGPGPGKATYEAALKQYEADLGPGPGKATYEAALKQYEADGPGPGAKATYEAALKQYEAGPGPGATYEAALKQYEADLGPGPGEDEQTSIKAALAELGPGPGPTPTPDQKKPPTRTPDKKPPTPTPDKKPTRTPDQKKTRTPDQAKKPDQAEPNKKHHHHHH

۳۸.

MAKLSTDELLDAFKEMTLLELSDFVKKFEETFEVTAAAPVAVAAAGAAPAGAAVEAAEEQSEFDVILEAAGDKKIGVIKVVREIVSGLGLKEAKDLVDGAPKPLLEKVAKEAADEAKAKLEAAGATVTVKEAAAKAKFVAAWTLKAAA**PMGLP**STNYYELTW**AAY**TYKNNFTLTV**AAY**DPTVHFHYF**AAY**NYYELTWDL**AAY**TVHFHYFKL**AAY**AYGIKSNVV**AAY**NAKATYEAALKQYEAGPGPGKATYEAALKQYEADLGPGPGKATYEAALKQYEADGPGPGAKATYEAALKQYEAGPGPGATYEAALKQYEADLGPGPGEDEQTSIKAALAELGPGPGPTPTPDQKKPPTRTPDKKPPTPTPDKKPTRTPDQKKTRTPDQAKKPDQAEPNKKHHHHHH

39.

MAKLSTDELLDAFKEMTLLELSDFVKKFEETFEVTAAAPVAVAAAGAAPAGAAVEAAEEQSEFDVILEAAGDKKIGVIKVVREIVSGLGLKEAKDLVDGAPKPLLEKVAKEAADEAKAKLEAAGATVTVKEAAAKAKFVAAWTLKAAA**PMGLP**STNYYELTW**AAY**TYKNNFTLTV**AAY**DPTVHFHYF**AAY**NYYELTWDL**AAY**AYGIKSNVV**AAY**TVHFHYFKL**AAY**NAKATYEAALKQYEAGPGPGKATYEAALKQYEADLGPGPGKATYEAALKQYEADGPGPGAKATYEAALKQYEAGPGPGATYEAALKQYEADLGPGPGEDEQTSIKAALAELGPGPGPTPTPDQKKPPTRTPDKKPPTPTPDKKPTRTPDQKKTRTPDQAKKPDQAEPNKKHHHHHH

40.

MAKLSTDELLDAFKEMTLLELSDFVKKFEETFEVTAAAPVAVAAAGAAPAGAAVEAAEEQSEFDVILEAAGDKKIGVIKVVREIVSGLGLKEAKDLVDGAPKPLLEKVAKEAADEAKAKLEAAGATVTVKEAAAKAKFVAAWTLKAAA**PMGLP**TYKNNFTLTV**AAY**NYYELTWDL**AAY**TVHFHYFKL**AAY**DPTVHFHYF**AAY**AYGIKSNVV**AAY**STNYYELTW**AAY**NAKATYEAALKQYEAGPGPGKATYEAALKQYEADLGPGPGKATYEAALKQYEADGPGPGAKATYEAALKQYEAGPGPGATYEAALKQYEADLGPGPGEDEQTSIKAALAELGPGPGPTPTPDQKKPPTRTPDKKPPTPTPDKKPTRTPDQKKTRTPDQAKKPDQAEPNKKHHHHHH

41.

MAKLSTDELLDAFKEMTLLELSDFVKKFEETFEVTAAAPVAVAAAGAAPAGAAVEAAEEQSEFDVILEAAGDKKIGVIKVVREIVSGLGLKEAKDLVDGAPKPLLEKVAKEAADEAKAKLEAAGATVTVKEAAAKAKFVAAWTLKAAA**PMGLP**TYKNNFTLTV**AAY**TVHFHYFKL**AAY**DPTVHFHYF**AAY**AYGIKSNVV**AAY**STNYYELTW**AAY**NYYELTWDL**AAY**NAKATYEAALKQYEAGPGPGKATYEAALKQYEADLGPGPGKATYEAALKQYEADGPGPGAKATYEAALKQYEAGPGPGATYEAALKQYEADLGPGPGEDEQTSIKAALAELGPGPGPTPTPDQKKPPTRTPDKKPPTPTPDKKPTRTPDQKKTRTPDQAKKPDQAEPNKKHHHHHH

42.

MAKLSTDELLDAFKEMTLLELSDFVKKFEETFEVTAAAPVAVAAAGAAPAGAAVEAAEEQSEFDVILEAAGDKKIGVIKVVREIVSGLGLKEAKDLVDGAPKPLLEKVAKEAADEAKAKLEAAGATVTVKEAAAKAKFVAAWTLKAAA**PMGLP**TYKNNFTLTV**AAY**NYYELTWDL**AAY**DPTVHFHYF**AAY**AYGIKSNVV**AAY**STNYYELTW**AAY**TVHFHYFKL**AAY**NAKATYEAALKQYEAGPGPGKATYEAALKQYEADLGPGPGKATYEAALKQYEADGPGPGAKATYEAALKQYEAGPGPGATYEAALKQYEADLGPGPGEDEQTSIKAALAELGPGPGPTPTPDQKKPPTRTPDKKPPTPTPDKKPTRTPDQKKTRTPDQAKKPDQAEPNKKHHHHHH

43.

MAKLSTDELLDAFKEMTLLELSDFVKKFEETFEVTAAAPVAVAAAGAAPAGAAVEAAEEQSEFDVILEAAGDKKIGVIKVVREIVSGLGLKEAKDLVDGAPKPLLEKVAKEAADEAKAKLEAAGATVTVKEAAAKAKFVAAWTLKAAA**PMGLP**TYKNNFTLTV**AAY**NYYELTWDL**AAY**AYGIKSNVV**AAY**STNYYELTW**AAY**TVHFHYFKL**AAY**DPTVHFHYF**AAY**NAKATYEAALKQYEAGPGPGKATYEAALKQYEADLGPGPGKATYEAALKQYEADGPGPGAKATYEAALKQYEAGPGPGATYEAALKQYEADLGPGPGEDEQTSIKAALAELGPGPGPTPTPDQKKPPTRTPDKKPPTPTPDKKPTRTPDQKKTRTPDQAKKPDQAEPNKKHHHHHH

44.

MAKLSTDELLDAFKEMTLLELSDFVKKFEETFEVTAAAPVAVAAAGAAPAGAAVEAAEEQSEFDVILEAAGDKKIGVIKVVREIVSGLGLKEAKDLVDGAPKPLLEKVAKEAADEAKAKLEAAGATVTVKEAAAKAKFVAAWTLKAAA**PMGLP**TYKNNFTLTV**AAY**NYYELTWDL**AAY**AYGIKSNVV**AAY**STNYYELTW**AAY**DPTVHFHYF**AAY**TVHFHYFKL**AAY**NAKATYEAALKQYEAGPGPGKATYEAALKQYEADLGPGPGKATYEAALKQYEADGPGPGAKATYEAALKQYEAGPGPGATYEAALKQYEADLGPGPGEDEQTSIKAALAELGPGPGPTPTPDQKKPPTRTPDKKPPTPTPDKKPTRTPDQKKTRTPDQAKKPDQAEPNKKHHHHHH

45.

MAKLSTDELLDAFKEMTLLELSDFVKKFEETFEVTAAAPVAVAAAGAAPAGAAVEAAEEQSEFDVILEAAGDKKIGVIKVVREIVSGLGLKEAKDLVDGAPKPLLEKVAKEAADEAKAKLEAAGATVTVKEAAAKAKFVAAWTLKAAA**PMGLP**TYKNNFTLTV**AAY**NYYELTWDL**AAY**STNYYELTW**AAY**AYGIKSNVV**AAY**DPTVHFHYF**AAY**TVHFHYFKL**AAY**NAKATYEAALKQYEAGPGPGKATYEAALKQYEADLGPGPGKATYEAALKQYEADGPGPGAKATYEAALKQYEAGPGPGATYEAALKQYEADLGPGPGEDEQTSIKAALAELGPGPGPTPTPDQKKPPTRTPDKKPPTPTPDKKPTRTPDQKKTRTPDQAKKPDQAEPNKKHHHHHH

46.

MAKLSTDELLDAFKEMTLLELSDFVKKFEETFEVTAAAPVAVAAAGAAPAGAAVEAAEEQSEFDVILEAAGDKKIGVIKVVREIVSGLGLKEAKDLVDGAPKPLLEKVAKEAADEAKAKLEAAGATVTVKEAAAKAKFVAAWTLKAAA**PMGLP**TYKNNFTLTV**AAY**NYYELTWDL**AAY**STNYYELTW**AAY**AYGIKSNVV**AAY**TVHFHYFKL**AAY**DPTVHFHYF**AAY**NAKATYEAALKQYEAGPGPGKATYEAALKQYEADLGPGPGKATYEAALKQYEADGPGPGAKATYEAALKQYEAGPGPGATYEAALKQYEADLGPGPGEDEQTSIKAALAELGPGPGPTPTPDQKKPPTRTPDKKPPTPTPDKKPTRTPDQKKTRTPDQAKKPDQAEPNKKHHHHHH

47.

MAKLSTDELLDAFKEMTLLELSDFVKKFEETFEVTAAAPVAVAAAGAAPAGAAVEAAEEQSEFDVILEAAGDKKIGVIKVVREIVSGLGLKEAKDLVDGAPKPLLEKVAKEAADEAKAKLEAAGATVTVKEAAAKAKFVAAWTLKAAA**PMGLP**TYKNNFTLTV**AAY**NYYELTWDL**AAY**AYGIKSNVV**AAY**STNYYELTW**AAY**DPTVHFHYF**AAY**TVHFHYFKL**AAY**NAKATYEAALKQYEAGPGPGKATYEAALKQYEADLGPGPGKATYEAALKQYEADGPGPGAKATYEAALKQYEAGPGPGATYEAALKQYEADLGPGPGEDEQTSIKAALAELGPGPGPTPTPDQKKPPTRTPDKKPPTPTPDKKPTRTPDQKKTRTPDQAKKPDQAEPNKKHHHHHH

48.

MAKLSTDELLDAFKEMTLLELSDFVKKFEETFEVTAAAPVAVAAAGAAPAGAAVEAAEEQSEFDVILEAAGDKKIGVIKVVREIVSGLGLKEAKDLVDGAPKPLLEKVAKEAADEAKAKLEAAGATVTVKEAAAKAKFVAAWTLKAAA**PMGLP**TYKNNFTLTV**AAY**NYYELTWDL**AAY**AYGIKSNVV**AAY**STNYYELTW**AAY**TVHFHYFKL**AAY**DPTVHFHYF**AAY**NAKATYEAALKQYEAGPGPGKATYEAALKQYEADLGPGPGKATYEAALKQYEADGPGPGAKATYEAALKQYEAGPGPGATYEAALKQYEADLGPGPGEDEQTSIKAALAELGPGPGPTPTPDQKKPPTRTPDKKPPTPTPDKKPTRTPDQKKTRTPDQAKKPDQAEPNKKHHHHHH

49.

MAKLSTDELLDAFKEMTLLELSDFVKKFEETFEVTAAAPVAVAAAGAAPAGAAVEAAEEQSEFDVILEAAGDKKIGVIKVVREIVSGLGLKEAKDLVDGAPKPLLEKVAKEAADEAKAKLEAAGATVTVKEAAAKAKFVAAWTLKAAAPMGLPNYYELTWDLAAYTVHFHYFKLAAYDPTVHFHYFAAYAYGIKSNVVAAYSTNYYELTWAAYTYKNNFTLTVAAYNAKATYEAALKQYEA**GPGPG**KATYEAALKQYEADL**GPGPG**KATYEAALKQYEAD**GPGPG**AKATYEAALKQYEA**GPGPG**ATYEAALKQYEADL**GPGPG**EDEQTSIKAALAEL**GPGPG**PTPTPDQKKPPTRTPDKKPPTPTPDKKPTRTPDQKKTRTPDQAKKPDQAEPNKKHHHHHH

50.

MAKLSTDELLDAFKEMTLLELSDFVKKFEETFEVTAAAPVAVAAAGAAPAGAAVEAAEEQSEFDVILEAAGDKKIGVIKVVREIVSGLGLKEAKDLVDGAPKPLLEKVAKEAADEAKAKLEAAGATVTVKEAAAKAKFVAAWTLKAAAPMGLPNYYELTWDLAAYTVHFHYFKLAAYDPTVHFHYFAAYAYGIKSNVVAAYSTNYYELTWAAYTYKNNFTLTVAAYNAKATYEAALKQYEA**GPGPG**KATYEAALKQYEADL**GPGPG**KATYEAALKQYEAD**GPGPG**AKATYEAALKQYEA**GPGPG**EDEQTSIKAALAEL**GPGPG**ATYEAALKQYEADL**GPGPG**PTPTPDQKKPPTRTPDKKPPTPTPDKKPTRTPDQKKTRTPDQAKKPDQAEPNKKHHHHHH

51.

MAKLSTDELLDAFKEMTLLELSDFVKKFEETFEVTAAAPVAVAAAGAAPAGAAVEAAEEQSEFDVILEAAGDKKIGVIKVVREIVSGLGLKEAKDLVDGAPKPLLEKVAKEAADEAKAKLEAAGATVTVKEAAAKAKFVAAWTLKAAAPMGLPNYYELTWDLAAYTVHFHYFKLAAYDPTVHFHYFAAYAYGIKSNVVAAYSTNYYELTWAAYTYKNNFTLTVAAYNAKATYEAALKQYEA**GPGPG**KATYEAALKQYEADL**GPGPG**KATYEAALKQYEAD**GPGPG**EDEQTSIKAALAEL**GPGPG**ATYEAALKQYEADL**GPGPG**AKATYEAALKQYEA**GPGPG**PTPTPDQKKPPTRTPDKKPPTPTPDKKPTRTPDQKKTRTPDQAKKPDQAEPNKKHHHHHH

52.

MAKLSTDELLDAFKEMTLLELSDFVKKFEETFEVTAAAPVAVAAAGAAPAGAAVEAAEEQSEFDVILEAAGDKKIGVIKVVREIVSGLGLKEAKDLVDGAPKPLLEKVAKEAADEAKAKLEAAGATVTVKEAAAKAKFVAAWTLKAAAPMGLPNYYELTWDLAAYTVHFHYFKLAAYDPTVHFHYFAAYAYGIKSNVVAAYSTNYYELTWAAYTYKNNFTLTVAAYNAKATYEAALKQYEA**GPGPG**KATYEAALKQYEADL**GPGPG**KATYEAALKQYEAD**GPGPG**EDEQTSIKAALAEL**GPGPG**AKATYEAALKQYEA**GPGPG**ATYEAALKQYEADL**GPGPG**PTPTPDQKKPPTRTPDKKPPTPTPDKKPTRTPDQKKTRTPDQAKKPDQAEPNKKHHHHHH

53.

MAKLSTDELLDAFKEMTLLELSDFVKKFEETFEVTAAAPVAVAAAGAAPAGAAVEAAEEQSEFDVILEAAGDKKIGVIKVVREIVSGLGLKEAKDLVDGAPKPLLEKVAKEAADEAKAKLEAAGATVTVKEAAAKAKFVAAWTLKAAAPMGLPNYYELTWDLAAYTVHFHYFKLAAYDPTVHFHYFAAYAYGIKSNVVAAYSTNYYELTWAAYTYKNNFTLTVAAYNAKATYEAALKQYEA**GPGPG**KATYEAALKQYEADL**GPGPG**KATYEAALKQYEAD**GPGPG**ATYEAALKQYEADL**GPGPG**EDEQTSIKAALAEL**GPGPG**AKATYEAALKQYEA**GPGPG**PTPTPDQKKPPTRTPDKKPPTPTPDKKPTRTPDQKKTRTPDQAKKPDQAEPNKKHHHHHH

54.

MAKLSTDELLDAFKEMTLLELSDFVKKFEETFEVTAAAPVAVAAAGAAPAGAAVEAAEEQSEFDVILEAAGDKKIGVIKVVREIVSGLGLKEAKDLVDGAPKPLLEKVAKEAADEAKAKLEAAGATVTVKEAAAKAKFVAAWTLKAAAPMGLPNYYELTWDLAAYTVHFHYFKLAAYDPTVHFHYFAAYAYGIKSNVVAAYSTNYYELTWAAYTYKNNFTLTVAAYNAKATYEAALKQYEA**GPGPG**KATYEAALKQYEADL**GPGPG**KATYEAALKQYEAD**GPGPG**ATYEAALKQYEADL**GPGPG**AKATYEAALKQYEA**GPGPG**EDEQTSIKAALAEL**GPGPG**PTPTPDQKKPPTRTPDKKPPTPTPDKKPTRTPDQKKTRTPDQAKKPDQAEPNKKHHHHHH

55.

MAKLSTDELLDAFKEMTLLELSDFVKKFEETFEVTAAAPVAVAAAGAAPAGAAVEAAEEQSEFDVILEAAGDKKIGVIKVVREIVSGLGLKEAKDLVDGAPKPLLEKVAKEAADEAKAKLEAAGATVTVKEAAAKAKFVAAWTLKAAAPMGLPNYYELTWDLAAYTVHFHYFKLAAYDPTVHFHYFAAYAYGIKSNVVAAYSTNYYELTWAAYTYKNNFTLTVAAYNAKATYEAALKQYEA**GPGPG**KATYEAALKQYEADL**GPGPG**AKATYEAALKQYEA**GPGPG**KATYEAALKQYEAD**GPGPG**ATYEAALKQYEADL**GPGPG**EDEQTSIKAALAEL**GPGPG**PTPTPDQKKPPTRTPDKKPPTPTPDKKPTRTPDQKKTRTPDQAKKPDQAEPNKKHHHHHH

56.

MAKLSTDELLDAFKEMTLLELSDFVKKFEETFEVTAAAPVAVAAAGAAPAGAAVEAAEEQSEFDVILEAAGDKKIGVIKVVREIVSGLGLKEAKDLVDGAPKPLLEKVAKEAADEAKAKLEAAGATVTVKEAAAKAKFVAAWTLKAAAPMGLPNYYELTWDLAAYTVHFHYFKLAAYDPTVHFHYFAAYAYGIKSNVVAAYSTNYYELTWAAYTYKNNFTLTVAAYNAKATYEAALKQYEA**GPGPG**KATYEAALKQYEADL**GPGPG**AKATYEAALKQYEA**GPGPG**KATYEAALKQYEAD**GPGPG**EDEQTSIKAALAEL**GPGPG**ATYEAALKQYEADL**GPGPG**PTPTPDQKKPPTRTPDKKPPTPTPDKKPTRTPDQKKTRTPDQAKKPDQAEPNKKHHHHHH

57.

MAKLSTDELLDAFKEMTLLELSDFVKKFEETFEVTAAAPVAVAAAGAAPAGAAVEAAEEQSEFDVILEAAGDKKIGVIKVVREIVSGLGLKEAKDLVDGAPKPLLEKVAKEAADEAKAKLEAAGATVTVKEAAAKAKFVAAWTLKAAAPMGLPNYYELTWDLAAYTVHFHYFKLAAYDPTVHFHYFAAYAYGIKSNVVAAYSTNYYELTWAAYTYKNNFTLTVAAYNAKATYEAALKQYEA**GPGPG**KATYEAALKQYEADL**GPGPG**AKATYEAALKQYEA**GPGPG**EDEQTSIKAALAEL**GPGPG**ATYEAALKQYEADL**GPGPG**KATYEAALKQYEAD**GPGPG**PTPTPDQKKPPTRTPDKKPPTPTPDKKPTRTPDQKKTRTPDQAKKPDQAEPNKKHHHHHH

58.

MAKLSTDELLDAFKEMTLLELSDFVKKFEETFEVTAAAPVAVAAAGAAPAGAAVEAAEEQSEFDVILEAAGDKKIGVIKVVREIVSGLGLKEAKDLVDGAPKPLLEKVAKEAADEAKAKLEAAGATVTVKEAAAKAKFVAAWTLKAAAPMGLPNYYELTWDLAAYTVHFHYFKLAAYDPTVHFHYFAAYAYGIKSNVVAAYSTNYYELTWAAYTYKNNFTLTVAAYNAKATYEAALKQYEA**GPGPG**KATYEAALKQYEADL**GPGPG**AKATYEAALKQYEA**GPGPG**EDEQTSIKAALAEL**GPGPG**KATYEAALKQYEAD**GPGPG**ATYEAALKQYEADL**GPGPG**PTPTPDQKKPPTRTPDKKPPTPTPDKKPTRTPDQKKTRTPDQAKKPDQAEPNKKHHHHHH

59.

MAKLSTDELLDAFKEMTLLELSDFVKKFEETFEVTAAAPVAVAAAGAAPAGAAVEAAEEQSEFDVILEAAGDKKIGVIKVVREIVSGLGLKEAKDLVDGAPKPLLEKVAKEAADEAKAKLEAAGATVTVKEAAAKAKFVAAWTLKAAAPMGLPNYYELTWDLAAYTVHFHYFKLAAYDPTVHFHYFAAYAYGIKSNVVAAYSTNYYELTWAAYTYKNNFTLTVAAYNAKATYEAALKQYEA**GPGPG**KATYEAALKQYEADL**GPGPG**AKATYEAALKQYEA**GPGPG**ATYEAALKQYEADL**GPGPG**KATYEAALKQYEAD**GPGPG**EDEQTSIKAALAEL**GPGPG**PTPTPDQKKPPTRTPDKKPPTPTPDKKPTRTPDQKKTRTPDQAKKPDQAEPNKKHHHHHH

60.

MAKLSTDELLDAFKEMTLLELSDFVKKFEETFEVTAAAPVAVAAAGAAPAGAAVEAAEEQSEFDVILEAAGDKKIGVIKVVREIVSGLGLKEAKDLVDGAPKPLLEKVAKEAADEAKAKLEAAGATVTVKEAAAKAKFVAAWTLKAAAPMGLPNYYELTWDLAAYTVHFHYFKLAAYDPTVHFHYFAAYAYGIKSNVVAAYSTNYYELTWAAYTYKNNFTLTVAAYNAKATYEAALKQYEA**GPGPG**KATYEAALKQYEADL**GPGPG**AKATYEAALKQYEA**GPGPG**ATYEAALKQYEADL**GPGPG**EDEQTSIKAALAEL**GPGPG**KATYEAALKQYEAD**GPGPG**PTPTPDQKKPPTRTPDKKPPTPTPDKKPTRTPDQKKTRTPDQAKKPDQAEPNKKHHHHHH

61.

MAKLSTDELLDAFKEMTLLELSDFVKKFEETFEVTAAAPVAVAAAGAAPAGAAVEAAEEQSEFDVILEAAGDKKIGVIKVVREIVSGLGLKEAKDLVDGAPKPLLEKVAKEAADEAKAKLEAAGATVTVKEAAAKAKFVAAWTLKAAAPMGLPNYYELTWDLAAYTVHFHYFKLAAYDPTVHFHYFAAYAYGIKSNVVAAYSTNYYELTWAAYTYKNNFTLTVAAYNAKATYEAALKQYEA**GPGPG**KATYEAALKQYEADL**GPGPG**ATYEAALKQYEADL**GPGPG**EDEQTSIKAALAEL**GPGPG**KATYEAALKQYEAD**GPGPG**AKATYEAALKQYEA**GPGPG**PTPTPDQKKPPTRTPDKKPPTPTPDKKPTRTPDQKKTRTPDQAKKPDQAEPNKKHHHHHH

62.

MAKLSTDELLDAFKEMTLLELSDFVKKFEETFEVTAAAPVAVAAAGAAPAGAAVEAAEEQSEFDVILEAAGDKKIGVIKVVREIVSGLGLKEAKDLVDGAPKPLLEKVAKEAADEAKAKLEAAGATVTVKEAAAKAKFVAAWTLKAAAPMGLPNYYELTWDLAAYTVHFHYFKLAAYDPTVHFHYFAAYAYGIKSNVVAAYSTNYYELTWAAYTYKNNFTLTVAAYNAKATYEAALKQYEA**GPGPG**KATYEAALKQYEADL**GPGPG**ATYEAALKQYEADL**GPGPG**EDEQTSIKAALAEL**GPGPG**AKATYEAALKQYEA**GPGPG**KATYEAALKQYEAD**GPGPG**PTPTPDQKKPPTRTPDKKPPTPTPDKKPTRTPDQKKTRTPDQAKKPDQAEPNKKHHHHHH

63.

MAKLSTDELLDAFKEMTLLELSDFVKKFEETFEVTAAAPVAVAAAGAAPAGAAVEAAEEQSEFDVILEAAGDKKIGVIKVVREIVSGLGLKEAKDLVDGAPKPLLEKVAKEAADEAKAKLEAAGATVTVKEAAAKAKFVAAWTLKAAAPMGLPNYYELTWDLAAYTVHFHYFKLAAYDPTVHFHYFAAYAYGIKSNVVAAYSTNYYELTWAAYTYKNNFTLTVAAYNAKATYEAALKQYEA**GPGPG**KATYEAALKQYEADL**GPGPG**AKATYEAALKQYEA**GPGPG**ATYEAALKQYEADL**GPGPG**KATYEAALKQYEAD**GPGPG**EDEQTSIKAALAEL**GPGPG**PTPTPDQKKPPTRTPDKKPPTPTPDKKPTRTPDQKKTRTPDQAKKPDQAEPNKKHHHHHH

64.

MAKLSTDELLDAFKEMTLLELSDFVKKFEETFEVTAAAPVAVAAAGAAPAGAAVEAAEEQSEFDVILEAAGDKKIGVIKVVREIVSGLGLKEAKDLVDGAPKPLLEKVAKEAADEAKAKLEAAGATVTVKEAAAKAKFVAAWTLKAAAPMGLPNYYELTWDLAAYTVHFHYFKLAAYDPTVHFHYFAAYAYGIKSNVVAAYSTNYYELTWAAYTYKNNFTLTVAAYNAKATYEAALKQYEA**GPGPG**KATYEAALKQYEADL**GPGPG**ATYEAALKQYEADL**GPGPG**AKATYEAALKQYEA**GPGPG**EDEQTSIKAALAEL**GPGPG**KATYEAALKQYEAD**GPGPG**PTPTPDQKKPPTRTPDKKPPTPTPDKKPTRTPDQKKTRTPDQAKKPDQAEPNKKHHHHHH

65.

MAKLSTDELLDAFKEMTLLELSDFVKKFEETFEVTAAAPVAVAAAGAAPAGAAVEAAEEQSEFDVILEAAGDKKIGVIKVVREIVSGLGLKEAKDLVDGAPKPLLEKVAKEAADEAKAKLEAAGATVTVKEAAAKAKFVAAWTLKAAAPMGLPNYYELTWDLAAYTVHFHYFKLAAYDPTVHFHYFAAYAYGIKSNVVAAYSTNYYELTWAAYTYKNNFTLTVAAYNAKATYEAALKQYEA**GPGPG**KATYEAALKQYEADL**GPGPG**ATYEAALKQYEADL**GPGPG**KATYEAALKQYEAD**GPGPG**AKATYEAALKQYEA**GPGPG**EDEQTSIKAALAEL**GPGPG**PTPTPDQKKPPTRTPDKKPPTPTPDKKPTRTPDQKKTRTPDQAKKPDQAEPNKKHHHHHH

66.

MAKLSTDELLDAFKEMTLLELSDFVKKFEETFEVTAAAPVAVAAAGAAPAGAAVEAAEEQSEFDVILEAAGDKKIGVIKVVREIVSGLGLKEAKDLVDGAPKPLLEKVAKEAADEAKAKLEAAGATVTVKEAAAKAKFVAAWTLKAAAPMGLPNYYELTWDLAAYTVHFHYFKLAAYDPTVHFHYFAAYAYGIKSNVVAAYSTNYYELTWAAYTYKNNFTLTVAAYNAKATYEAALKQYEA**GPGPG**KATYEAALKQYEADL**GPGPG**ATYEAALKQYEADL**GPGPG**KATYEAALKQYEAD**GPGPG**EDEQTSIKAALAEL**GPGPG**AKATYEAALKQYEA**GPGPG**PTPTPDQKKPPTRTPDKKPPTPTPDKKPTRTPDQKKTRTPDQAKKPDQAEPNKKHHHHHH

67.

MAKLSTDELLDAFKEMTLLELSDFVKKFEETFEVTAAAPVAVAAAGAAPAGAAVEAAEEQSEFDVILEAAGDKKIGVIKVVREIVSGLGLKEAKDLVDGAPKPLLEKVAKEAADEAKAKLEAAGATVTVKEAAAKAKFVAAWTLKAAAPMGLPNYYELTWDLAAYTVHFHYFKLAAYDPTVHFHYFAAYAYGIKSNVVAAYSTNYYELTWAAYTYKNNFTLTVAAYNAKATYEAALKQYEA**GPGPG**KATYEAALKQYEADL**GPGPG**EDEQTSIKAALAEL**GPGPG**KATYEAALKQYEAD**GPGPG**AKATYEAALKQYEA**GPGPG**ATYEAALKQYEADL**GPGPG**PTPTPDQKKPPTRTPDKKPPTPTPDKKPTRTPDQKKTRTPDQAKKPDQAEPNKKHHHHHH

68.

MAKLSTDELLDAFKEMTLLELSDFVKKFEETFEVTAAAPVAVAAAGAAPAGAAVEAAEEQSEFDVILEAAGDKKIGVIKVVREIVSGLGLKEAKDLVDGAPKPLLEKVAKEAADEAKAKLEAAGATVTVKEAAAKAKFVAAWTLKAAAPMGLPNYYELTWDLAAYTVHFHYFKLAAYDPTVHFHYFAAYAYGIKSNVVAAYSTNYYELTWAAYTYKNNFTLTVAAYNAKATYEAALKQYEA**GPGPG**KATYEAALKQYEADL**GPGPG**EDEQTSIKAALAEL**GPGPG**KATYEAALKQYEAD**GPGPG**ATYEAALKQYEADL**GPGPG**AKATYEAALKQYEA**GPGPG**PTPTPDQKKPPTRTPDKKPPTPTPDKKPTRTPDQKKTRTPDQAKKPDQAEPNKKHHHHHH

69.

MAKLSTDELLDAFKEMTLLELSDFVKKFEETFEVTAAAPVAVAAAGAAPAGAAVEAAEEQSEFDVILEAAGDKKIGVIKVVREIVSGLGLKEAKDLVDGAPKPLLEKVAKEAADEAKAKLEAAGATVTVKEAAAKAKFVAAWTLKAAAPMGLPNYYELTWDLAAYTVHFHYFKLAAYDPTVHFHYFAAYAYGIKSNVVAAYSTNYYELTWAAYTYKNNFTLTVAAYNAKATYEAALKQYEA**GPGPG**KATYEAALKQYEADL**GPGPG**EDEQTSIKAALAEL**GPGPG**ATYEAALKQYEADL**GPGPG**AKATYEAALKQYEA**GPGPG**KATYEAALKQYEAD**GPGPG**PTPTPDQKKPPTRTPDKKPPTPTPDKKPTRTPDQKKTRTPDQAKKPDQAEPNKKHHHHHH

70.

MAKLSTDELLDAFKEMTLLELSDFVKKFEETFEVTAAAPVAVAAAGAAPAGAAVEAAEEQSEFDVILEAAGDKKIGVIKVVREIVSGLGLKEAKDLVDGAPKPLLEKVAKEAADEAKAKLEAAGATVTVKEAAAKAKFVAAWTLKAAAPMGLPNYYELTWDLAAYTVHFHYFKLAAYDPTVHFHYFAAYAYGIKSNVVAAYSTNYYELTWAAYTYKNNFTLTVAAYNAKATYEAALKQYEA**GPGPG**KATYEAALKQYEADL**GPGPG**EDEQTSIKAALAEL**GPGPG**ATYEAALKQYEADL**GPGPG**KATYEAALKQYEAD**GPGPG**AKATYEAALKQYEA**GPGPG**PTPTPDQKKPPTRTPDKKPPTPTPDKKPTRTPDQKKTRTPDQAKKPDQAEPNKKHHHHHH

**71.**

MAKLSTDELLDAFKEMTLLELSDFVKKFEETFEVTAAAPVAVAAAGAAPAGAAVEAAEEQSEFDVILEAAGDKKIGVIKVVREIVSGLGLKEAKDLVDGAPKPLLEKVAKEAADEAKAKLEAAGATVTVKEAAAKAKFVAAWTLKAAAPMGLPNYYELTWDLAAYTVHFHYFKLAAYDPTVHFHYFAAYAYGIKSNVVAAYSTNYYELTWAAYTYKNNFTLTVAAYNAKATYEAALKQYEA**GPGPG**KATYEAALKQYEADL**GPGPG**EDEQTSIKAALAEL**GPGPG**AKATYEAALKQYEA**GPGPG**KATYEAALKQYEAD**GPGPG**ATYEAALKQYEADL**GPGPG**PTPTPDQKKPPTRTPDKKPPTPTPDKKPTRTPDQKKTRTPDQAKKPDQAEPNKKHHHHHH

**72.**

MAKLSTDELLDAFKEMTLLELSDFVKKFEETFEVTAAAPVAVAAAGAAPAGAAVEAAEEQSEFDVILEAAGDKKIGVIKVVREIVSGLGLKEAKDLVDGAPKPLLEKVAKEAADEAKAKLEAAGATVTVKEAAAKAKFVAAWTLKAAAPMGLPNYYELTWDLAAYTVHFHYFKLAAYDPTVHFHYFAAYAYGIKSNVVAAYSTNYYELTWAAYTYKNNFTLTVAAYNAKATYEAALKQYEA**GPGPG**KATYEAALKQYEADL**GPGPG**EDEQTSIKAALAEL**GPGPG**AKATYEAALKQYEA**GPGPG**ATYEAALKQYEADL**GPGPG**KATYEAALKQYEAD**GPGPG**PTPTPDQKKPPTRTPDKKPPTPTPDKKPTRTPDQKKTRTPDQAKKPDQAEPNKKHHHHHH

**73.**

MAKLSTDELLDAFKEMTLLELSDFVKKFEETFEVTAAAPVAVAAAGAAPAGAAVEAAEEQSEFDVILEAAGDKKIGVIKVVREIVSGLGLKEAKDLVDGAPKPLLEKVAKEAADEAKAKLEAAGATVTVKEAAAKAKFVAAWTLKAAAPMGLPNYYELTWDLAAYTVHFHYFKLAAYDPTVHFHYFAAYAYGIKSNVVAAYSTNYYELTWAAYTYKNNFTLTVAAYNAKATYEAALKQYEA**GPGPG**KATYEAALKQYEAD**GPGPG**AKATYEAALKQYEA**GPGPG**ATYEAALKQYEADL**GPGPG**EDEQTSIKAALAEL**GPGPG**KATYEAALKQYEADL**GPGPG**PTPTPDQKKPPTRTPDKKPPTPTPDKKPTRTPDQKKTRTPDQAKKPDQAEPNKKHHHHHH

**74.**

MAKLSTDELLDAFKEMTLLELSDFVKKFEETFEVTAAAPVAVAAAGAAPAGAAVEAAEEQSEFDVILEAAGDKKIGVIKVVREIVSGLGLKEAKDLVDGAPKPLLEKVAKEAADEAKAKLEAAGATVTVKEAAAKAKFVAAWTLKAAAPMGLPNYYELTWDLAAYTVHFHYFKLAAYDPTVHFHYFAAYAYGIKSNVVAAYSTNYYELTWAAYTYKNNFTLTVAAYNAKATYEAALKQYEA**GPGPG**KATYEAALKQYEAD**GPGPG**AKATYEAALKQYEA**GPGPG**ATYEAALKQYEADL**GPGPG**KATYEAALKQYEADL**GPGPG**EDEQTSIKAALAEL**GPGPG**PTPTPDQKKPPTRTPDKKPPTPTPDKKPTRTPDQKKTRTPDQAKKPDQAEPNKKHHHHHH

**75.**

MAKLSTDELLDAFKEMTLLELSDFVKKFEETFEVTAAAPVAVAAAGAAPAGAAVEAAEEQSEFDVILEAAGDKKIGVIKVVREIVSGLGLKEAKDLVDGAPKPLLEKVAKEAADEAKAKLEAAGATVTVKEAAAKAKFVAAWTLKAAAPMGLPNYYELTWDLAAYTVHFHYFKLAAYDPTVHFHYFAAYAYGIKSNVVAAYSTNYYELTWAAYTYKNNFTLTVAAYNAKATYEAALKQYEA**GPGPG**KATYEAALKQYEAD**GPGPG**AKATYEAALKQYEA**GPGPG**EDEQTSIKAALAEL**GPGPG**ATYEAALKQYEADL**GPGPG**KATYEAALKQYEADL**GPGPG**PTPTPDQKKPPTRTPDKKPPTPTPDKKPTRTPDQKKTRTPDQAKKPDQAEPNKKHHHHHH

**76.**

MAKLSTDELLDAFKEMTLLELSDFVKKFEETFEVTAAAPVAVAAAGAAPAGAAVEAAEEQSEFDVILEAAGDKKIGVIKVVREIVSGLGLKEAKDLVDGAPKPLLEKVAKEAADEAKAKLEAAGATVTVKEAAAKAKFVAAWTLKAAAPMGLPNYYELTWDLAAYTVHFHYFKLAAYDPTVHFHYFAAYAYGIKSNVVAAYSTNYYELTWAAYTYKNNFTLTVAAYNAKATYEAALKQYEA**GPGPG**KATYEAALKQYEAD**GPGPG**AKATYEAALKQYEA**GPGPG**EDEQTSIKAALAEL**GPGPG**KATYEAALKQYEADL**GPGPG**ATYEAALKQYEADL**GPGPG**PTPTPDQKKPPTRTPDKKPPTPTPDKKPTRTPDQKKTRTPDQAKKPDQAEPNKKHHHHHH

**77.**

MAKLSTDELLDAFKEMTLLELSDFVKKFEETFEVTAAAPVAVAAAGAAPAGAAVEAAEEQSEFDVILEAAGDKKIGVIKVVREIVSGLGLKEAKDLVDGAPKPLLEKVAKEAADEAKAKLEAAGATVTVKEAAAKAKFVAAWTLKAAAPMGLPNYYELTWDLAAYTVHFHYFKLAAYDPTVHFHYFAAYAYGIKSNVVAAYSTNYYELTWAAYTYKNNFTLTVAAYNAKATYEAALKQYEA**GPGPG**KATYEAALKQYEAD**GPGPG**AKATYEAALKQYEA**GPGPG**KATYEAALKQYEADL**GPGPG**EDEQTSIKAALAEL**GPGPG**ATYEAALKQYEADL**GPGPG**PTPTPDQKKPPTRTPDKKPPTPTPDKKPTRTPDQKKTRTPDQAKKPDQAEPNKKHHHHHH

**78.**

MAKLSTDELLDAFKEMTLLELSDFVKKFEETFEVTAAAPVAVAAAGAAPAGAAVEAAEEQSEFDVILEAAGDKKIGVIKVVREIVSGLGLKEAKDLVDGAPKPLLEKVAKEAADEAKAKLEAAGATVTVKEAAAKAKFVAAWTLKAAAPMGLPNYYELTWDLAAYTVHFHYFKLAAYDPTVHFHYFAAYAYGIKSNVVAAYSTNYYELTWAAYTYKNNFTLTVAAYNAKATYEAALKQYEA**GPGPG**KATYEAALKQYEAD**GPGPG**AKATYEAALKQYEA**GPGPG**KATYEAALKQYEADL**GPGPG**ATYEAALKQYEADL**GPGPG**EDEQTSIKAALAEL**GPGPG**PTPTPDQKKPPTRTPDKKPPTPTPDKKPTRTPDQKKTRTPDQAKKPDQAEPNKKHHHHHH

**79.**

MAKLSTDELLDAFKEMTLLELSDFVKKFEETFEVTAAAPVAVAAAGAAPAGAAVEAAEEQSEFDVILEAAGDKKIGVIKVVREIVSGLGLKEAKDLVDGAPKPLLEKVAKEAADEAKAKLEAAGATVTVKEAAAKAKFVAAWTLKAAAPMGLPNYYELTWDLAAYTVHFHYFKLAAYDPTVHFHYFAAYAYGIKSNVVAAYSTNYYELTWAAYTYKNNFTLTVAAYNAKATYEAALKQYEA**GPGPG**KATYEAALKQYEAD**GPGPG**KATYEAALKQYEADL**GPGPG**AKATYEAALKQYEA**GPGPG**ATYEAALKQYEADL**GPGPG**EDEQTSIKAALAEL**GPGPG**PTPTPDQKKPPTRTPDKKPPTPTPDKKPTRTPDQKKTRTPDQAKKPDQAEPNKKHHHHHH

**80.**

MAKLSTDELLDAFKEMTLLELSDFVKKFEETFEVTAAAPVAVAAAGAAPAGAAVEAAEEQSEFDVILEAAGDKKIGVIKVVREIVSGLGLKEAKDLVDGAPKPLLEKVAKEAADEAKAKLEAAGATVTVKEAAAKAKFVAAWTLKAAAPMGLPNYYELTWDLAAYTVHFHYFKLAAYDPTVHFHYFAAYAYGIKSNVVAAYSTNYYELTWAAYTYKNNFTLTVAAYNAKATYEAALKQYEA**GPGPG**KATYEAALKQYEAD**GPGPG**KATYEAALKQYEADL**GPGPG**EDEQTSIKAALAEL**GPGPG**ATYEAALKQYEADL**GPGPG**AKATYEAALKQYEA**GPGPG**PTPTPDQKKPPTRTPDKKPPTPTPDKKPTRTPDQKKTRTPDQAKKPDQAEPNKKHHHHHH

**81.**

MAKLSTDELLDAFKEMTLLELSDFVKKFEETFEVTAAAPVAVAAAGAAPAGAAVEAAEEQSEFDVILEAAGDKKIGVIKVVREIVSGLGLKEAKDLVDGAPKPLLEKVAKEAADEAKAKLEAAGATVTVKEAAAKAKFVAAWTLKAAAPMGLPNYYELTWDLAAYTVHFHYFKLAAYDPTVHFHYFAAYAYGIKSNVVAAYSTNYYELTWAAYTYKNNFTLTVAAYNAKATYEAALKQYEA**GPGPG**KATYEAALKQYEAD**GPGPG**KATYEAALKQYEADL**GPGPG**AKATYEAALKQYEA**GPGPG**EDEQTSIKAALAEL**GPGPG**ATYEAALKQYEADL**GPGPG**PTPTPDQKKPPTRTPDKKPPTPTPDKKPTRTPDQKKTRTPDQAKKPDQAEPNKKHHHHHH

**82.**

MAKLSTDELLDAFKEMTLLELSDFVKKFEETFEVTAAAPVAVAAAGAAPAGAAVEAAEEQSEFDVILEAAGDKKIGVIKVVREIVSGLGLKEAKDLVDGAPKPLLEKVAKEAADEAKAKLEAAGATVTVKEAAAKAKFVAAWTLKAAAPMGLPNYYELTWDLAAYTVHFHYFKLAAYDPTVHFHYFAAYAYGIKSNVVAAYSTNYYELTWAAYTYKNNFTLTVAAYNAKATYEAALKQYEA**GPGPG**KATYEAALKQYEAD**GPGPG**KATYEAALKQYEADL**GPGPG**EDEQTSIKAALAEL**GPGPG**AKATYEAALKQYEA**GPGPG**ATYEAALKQYEADL**GPGPG**PTPTPDQKKPPTRTPDKKPPTPTPDKKPTRTPDQKKTRTPDQAKKPDQAEPNKKHHHHHH

**83.**

MAKLSTDELLDAFKEMTLLELSDFVKKFEETFEVTAAAPVAVAAAGAAPAGAAVEAAEEQSEFDVILEAAGDKKIGVIKVVREIVSGLGLKEAKDLVDGAPKPLLEKVAKEAADEAKAKLEAAGATVTVKEAAAKAKFVAAWTLKAAA**PMGLP**NYYELTWDL**AAY**AYGIKSNVV**AAY**STNYYELTW**AAY**TYKNNFTLTV**AAY**TVHFHYFKL**AAY**DPTVHFHYFAAYNAKATYEAALKQYEA**GPGPG**KATYEAALKQYEAD**GPGPG**KATYEAALKQYEADL**GPGPG**EDEQTSIKAALAEL**GPGPG**AKATYEAALKQYEA**GPGPG**ATYEAALKQYEADL**GPGPG**PTPTPDQKKPPTRTPDKKPPTPTPDKKPTRTPDQKKTRTPDQAKKPDQAEPNKKHHHHHH

**84.**

MAKLSTDELLDAFKEMTLLELSDFVKKFEETFEVTAAAPVAVAAAGAAPAGAAVEAAEEQSEFDVILEAAGDKKIGVIKVVREIVSGLGLKEAKDLVDGAPKPLLEKVAKEAADEAKAKLEAAGATVTVKEAAAKAKFVAAWTLKAAA**PMGLP**NYYELTWDL**AAY**AYGIKSNVV**AAY**STNYYELTW**AAY**TYKNNFTLTV**AAY**TVHFHYFKL**AAY**DPTVHFHYF**AAY**NAKATYEAALKQYEA**GPGPG**KATYEAALKQYEAD**GPGPG**KATYEAALKQYEADL**GPGPG**EDEQTSIKAALAEL**GPGPG**ATYEAALKQYEADL**GPGPG**AKATYEAALKQYEA**GPGPG**PTPTPDQKKPPTRTPDKKPPTPTPDKKPTRTPDQKKTRTPDQAKKPDQAEPNKKHHHHHH

**85.**

MAKLSTDELLDAFKEMTLLELSDFVKKFEETFEVTAAAPVAVAAAGAAPAGAAVEAAEEQSEFDVILEAAGDKKIGVIKVVREIVSGLGLKEAKDLVDGAPKPLLEKVAKEAADEAKAKLEAAGATVTVKEAAAKAKFVAAWTLKAAA**PMGLP**NYYELTWDL**AAY**AYGIKSNVV**AAY**STNYYELTW**AAY**TYKNNFTLTV**AAY**TVHFHYFKL**AAY**DPTVHFHYF**AAY**NAKATYEAALKQYEA**GPGPG**KATYEAALKQYEAD**GPGPG**KATYEAALKQYEADL**GPGPG**AKATYEAALKQYEA**GPGPG**ATYEAALKQYEADL**GPGPG**EDEQTSIKAALAEL**GPGPG**PTPTPDQKKPPTRTPDKKPPTPTPDKKPTRTPDQKKTRTPDQAKKPDQAEPNKKHHHHHH

**86.**

MAKLSTDELLDAFKEMTLLELSDFVKKFEETFEVTAAAPVAVAAAGAAPAGAAVEAAEEQSEFDVILEAAGDKKIGVIKVVREIVSGLGLKEAKDLVDGAPKPLLEKVAKEAADEAKAKLEAAGATVTVKEAAAKAKFVAAWTLKAAA**PMGLP**NYYELTWDL**AAY**AYGIKSNVV**AAY**STNYYELTW**AAY**TYKNNFTLTV**AAY**TVHFHYFKL**AAY**DPTVHFHYF**AAY**NAKATYEAALKQYEA**GPGPG**KATYEAALKQYEAD**GPGPG**AKATYEAALKQYEA**GPGPG**KATYEAALKQYEADL**GPGPG**ATYEAALKQYEADL**GPGPG**EDEQTSIKAALAEL**GPGPG**PTPTPDQKKPPTRTPDKKPPTPTPDKKPTRTPDQKKTRTPDQAKKPDQAEPNKKHHHHHH

**87.**

MAKLSTDELLDAFKEMTLLELSDFVKKFEETFEVTAAAPVAVAAAGAAPAGAAVEAAEEQSEFDVILEAAGDKKIGVIKVVREIVSGLGLKEAKDLVDGAPKPLLEKVAKEAADEAKAKLEAAGATVTVKEAAAKAKFVAAWTLKAAA**PMGLP**NYYELTWDL**AAY**AYGIKSNVV**AAY**STNYYELTW**AAY**TYKNNFTLTV**AAY**TVHFHYFKL**AAY**DPTVHFHYF**AAY**NAKATYEAALKQYEA**GPGPG**KATYEAALKQYEAD**GPGPG**AKATYEAALKQYEA**GPGPG**KATYEAALKQYEADL**GPGPG**EDEQTSIKAALAEL**GPGPG**ATYEAALKQYEADL**GPGPG**PTPTPDQKKPPTRTPDKKPPTPTPDKKPTRTPDQKKTRTPDQAKKPDQAEPNKKHHHHHH

**88.**

MAKLSTDELLDAFKEMTLLELSDFVKKFEETFEVTAAAPVAVAAAGAAPAGAAVEAAEEQSEFDVILEAAGDKKIGVIKVVREIVSGLGLKEAKDLVDGAPKPLLEKVAKEAADEAKAKLEAAGATVTVKEAAAKAKFVAAWTLKAAA**PMGLP**NYYELTWDL**AAY**AYGIKSNVV**AAY**STNYYELTW**AAY**TYKNNFTLTV**AAY**TVHFHYFKL**AAY**DPTVHFHYF**AAY**NAKATYEAALKQYEA**GPGPG**KATYEAALKQYEAD**GPGPG**AKATYEAALKQYEA**GPGPG**EDEQTSIKAALAEL**GPGPG**KATYEAALKQYEADL**GPGPG**ATYEAALKQYEADL**GPGPG**PTPTPDQKKPPTRTPDKKPPTPTPDKKPTRTPDQKKTRTPDQAKKPDQAEPNKKHHHHHH

**89.**

MAKLSTDELLDAFKEMTLLELSDFVKKFEETFEVTAAAPVAVAAAGAAPAGAAVEAAEEQSEFDVILEAAGDKKIGVIKVVREIVSGLGLKEAKDLVDGAPKPLLEKVAKEAADEAKAKLEAAGATVTVKEAAAKAKFVAAWTLKAAA**PMGLP**NYYELTWDL**AAY**AYGIKSNVV**AAY**STNYYELTW**AAY**TYKNNFTLTV**AAY**TVHFHYFKL**AAY**DPTVHFHYF**AAY**NAKATYEAALKQYEA**GPGPG**KATYEAALKQYEAD**GPGPG**AKATYEAALKQYEA**GPGPG**EDEQTSIKAALAEL**GPGPG**ATYEAALKQYEADL**GPGPG**KATYEAALKQYEADL**GPGPG**PTPTPDQKKPPTRTPDKKPPTPTPDKKPTRTPDQKKTRTPDQAKKPDQAEPNKKHHHHHH

**90.**

MAKLSTDELLDAFKEMTLLELSDFVKKFEETFEVTAAAPVAVAAAGAAPAGAAVEAAEEQSEFDVILEAAGDKKIGVIKVVREIVSGLGLKEAKDLVDGAPKPLLEKVAKEAADEAKAKLEAAGATVTVKEAAAKAKFVAAWTLKAAA**PMGLP**NYYELTWDL**AAY**AYGIKSNVV**AAY**STNYYELTW**AAY**TYKNNFTLTV**AAY**TVHFHYFKL**AAY**DPTVHFHYF**AAY**NAKATYEAALKQYEA**GPGPG**KATYEAALKQYEAD**GPGPG**AKATYEAALKQYEA**GPGPG**ATYEAALKQYEADL**GPGPG**KATYEAALKQYEADL**GPGPG**EDEQTSIKAALAEL**GPGPG**PTPTPDQKKPPTRTPDKKPPTPTPDKKPTRTPDQKKTRTPDQAKKPDQAEPNKKHHHHHH

**91.**

MAKLSTDELLDAFKEMTLLELSDFVKKFEETFEVTAAAPVAVAAAGAAPAGAAVEAAEEQSEFDVILEAAGDKKIGVIKVVREIVSGLGLKEAKDLVDGAPKPLLEKVAKEAADEAKAKLEAAGATVTVKEAAAKAKFVAAWTLKAAA**PMGLP**NYYELTWDL**AAY**AYGIKSNVV**AAY**STNYYELTW**AAY**TYKNNFTLTV**AAY**TVHFHYFKL**AAY**DPTVHFHYF**AAY**NAKATYEAALKQYEA**GPGPG**KATYEAALKQYEAD**GPGPG**AKATYEAALKQYEA**GPGPG**ATYEAALKQYEADL**GPGPG**EDEQTSIKAALAEL**GPGPG**KATYEAALKQYEADL**GPGPG**PTPTPDQKKPPTRTPDKKPPTPTPDKKPTRTPDQKKTRTPDQAKKPDQAEPNKKHHHHHH

**92.**

MAKLSTDELLDAFKEMTLLELSDFVKKFEETFEVTAAAPVAVAAAGAAPAGAAVEAAEEQSEFDVILEAAGDKKIGVIKVVREIVSGLGLKEAKDLVDGAPKPLLEKVAKEAADEAKAKLEAAGATVTVKEAAAKAKFVAAWTLKAAA**PMGLP**NYYELTWDL**AAY**AYGIKSNVV**AAY**STNYYELTW**AAY**TYKNNFTLTV**AAY**TVHFHYFKL**AAY**DPTVHFHYF**AAY**NAKATYEAALKQYEA**GPGPG**KATYEAALKQYEADL**GPGPG**EDEQTSIKAALAEL**GPGPG**AKATYEAALKQYEA**GPGPG**ATYEAALKQYEADL**GPGPG**KATYEAALKQYEAD**GPGPG**PTPTPDQKKPPTRTPDKKPPTPTPDKKPTRTPDQKKTRTPDQAKKPDQAEPNKKHHHHHH

**93.**

MAKLSTDELLDAFKEMTLLELSDFVKKFEETFEVTAAAPVAVAAAGAAPAGAAVEAAEEQSEFDVILEAAGDKKIGVIKVVREIVSGLGLKEAKDLVDGAPKPLLEKVAKEAADEAKAKLEAAGATVTVKEAAAKAKFVAAWTLKAAA**PMGLP**NYYELTWDL**AAY**AYGIKSNVV**AAY**STNYYELTW**AAY**TYKNNFTLTV**AAY**TVHFHYFKL**AAY**DPTVHFHYF**AAY**NAKATYEAALKQYEA**GPGPG**KATYEAALKQYEADL**GPGPG**EDEQTSIKAALAEL**GPGPG**AKATYEAALKQYEA**GPGPG**KATYEAALKQYEAD**GPGPG**ATYEAALKQYEADL**GPGPG**PTPTPDQKKPPTRTPDKKPPTPTPDKKPTRTPDQKKTRTPDQAKKPDQAEPNKKHHHHHH

**94.**

MAKLSTDELLDAFKEMTLLELSDFVKKFEETFEVTAAAPVAVAAAGAAPAGAAVEAAEEQSEFDVILEAAGDKKIGVIKVVREIVSGLGLKEAKDLVDGAPKPLLEKVAKEAADEAKAKLEAAGATVTVKEAAAKAKFVAAWTLKAAA**PMGLP**NYYELTWDL**AAY**AYGIKSNVV**AAY**STNYYELTW**AAY**TYKNNFTLTV**AAY**TVHFHYFKL**AAY**DPTVHFHYF**AAY**NAKATYEAALKQYEA**GPGPG**KATYEAALKQYEADL**GPGPG**EDEQTSIKAALAEL**GPGPG**ATYEAALKQYEADL**GPGPG**KATYEAALKQYEAD**GPGPG**AKATYEAALKQYEA**GPGPG**PTPTPDQKKPPTRTPDKKPPTPTPDKKPTRTPDQKKTRTPDQAKKPDQAEPNKKHHHHHH

**95.**

MAKLSTDELLDAFKEMTLLELSDFVKKFEETFEVTAAAPVAVAAAGAAPAGAAVEAAEEQSEFDVILEAAGDKKIGVIKVVREIVSGLGLKEAKDLVDGAPKPLLEKVAKEAADEAKAKLEAAGATVTVKEAAAKAKFVAAWTLKAAA**PMGLP**NYYELTWDL**AAY**AYGIKSNVV**AAY**STNYYELTW**AAY**TYKNNFTLTV**AAY**TVHFHYFKL**AAY**DPTVHFHYF**AAY** NAKATYEAALKQYEA**GPGPG**KATYEAALKQYEADL**GPGPG**EDEQTSIKAALAEL**GPGPG**ATYEAALKQYEADL**GPGPG**AKATYEAALKQYEA**GPGPG**KATYEAALKQYEAD**GPGPG**PTPTPDQKKPPTRTPDKKPPTPTPDKKPTRTPDQKKTRTPDQAKKPDQAEPNKKHHHHHH

**96.**

MAKLSTDELLDAFKEMTLLELSDFVKKFEETFEVTAAAPVAVAAAGAAPAGAAVEAAEEQSEFDVILEAAGDKKIGVIKVVREIVSGLGLKEAKDLVDGAPKPLLEKVAKEAADEAKAKLEAAGATVTVKEAAAKAKFVAAWTLKAAA**PMGLP**NYYELTWDL**AAY**AYGIKSNVV**AAY**STNYYELTW**AAY**TYKNNFTLTV**AAY**TVHFHYFKL**AAY**DPTVHFHYF**AAY**NAKATYEAALKQYEA**GPGPG**KATYEAALKQYEADL**GPGPG**EDEQTSIKAALAEL**GPGPG**KATYEAALKQYEAD**GPGPG**ATYEAALKQYEADL**GPGPG**AKATYEAALKQYEA**GPGPG**PTPTPDQKKPPTRTPDKKPPTPTPDKKPTRTPDQKKTRTPDQAKKPDQAEPNKKHHHHHH

**97.**

MAKLSTDELLDAFKEMTLLELSDFVKKFEETFEVTAAAPVAVAAAGAAPAGAAVEAAEEQSEFDVILEAAGDKKIGVIKVVREIVSGLGLKEAKDLVDGAPKPLLEKVAKEAADEAKAKLEAAGATVTVKEAAAKAKFVAAWTLKAAA**PMGLP**NYYELTWDL**AAY**AYGIKSNVV**AAY**STNYYELTW**AAY**TYKNNFTLTV**AAY**TVHFHYFKL**AAY**DPTVHFHYF**AAY**NAKATYEAALKQYEA**GPGPG**KATYEAALKQYEADL**GPGPG**EDEQTSIKAALAEL**GPGPG**KATYEAALKQYEAD**GPGPG**AKATYEAALKQYEA**GPGPG**ATYEAALKQYEADL**GPGPG**PTPTPDQKKPPTRTPDKKPPTPTPDKKPTRTPDQKKTRTPDQAKKPDQAEPNKKHHHHHH

**98.**

MAKLSTDELLDAFKEMTLLELSDFVKKFEETFEVTAAAPVAVAAAGAAPAGAAVEAAEEQSEFDVILEAAGDKKIGVIKVVREIVSGLGLKEAKDLVDGAPKPLLEKVAKEAADEAKAKLEAAGATVTVKEAAAKAKFVAAWTLKAAA**PMGLP**NYYELTWDL**AAY**AYGIKSNVV**AAY**STNYYELTW**AAY**TYKNNFTLTV**AAY**TVHFHYFKL**AAY**DPTVHFHYF**AAY**NAKATYEAALKQYEA**GPGPG**KATYEAALKQYEADL**GPGPG**ATYEAALKQYEADL**GPGPG**KATYEAALKQYEAD**GPGPG**EDEQTSIKAALAEL**GPGPG**AKATYEAALKQYEA**GPGPG**PTPTPDQKKPPTRTPDKKPPTPTPDKKPTRTPDQKKTRTPDQAKKPDQAEPNKKHHHHHH

**99.**

MAKLSTDELLDAFKEMTLLELSDFVKKFEETFEVTAAAPVAVAAAGAAPAGAAVEAAEEQSEFDVILEAAGDKKIGVIKVVREIVSGLGLKEAKDLVDGAPKPLLEKVAKEAADEAKAKLEAAGATVTVKEAAAKAKFVAAWTLKAAA**PMGLP**NYYELTWDL**AAY**AYGIKSNVV**AAY**STNYYELTW**AAY**TYKNNFTLTV**AAY**TVHFHYFKL**AAY**DPTVHFHYF**AAY**NAKATYEAALKQYEA**GPGPG**KATYEAALKQYEADL**GPGPG**ATYEAALKQYEADL**GPGPG**AKATYEAALKQYEA**GPGPG**EDEQTSIKAALAEL**GPGPG**KATYEAALKQYEAD**GPGPG**PTPTPDQKKPPTRTPDKKPPTPTPDKKPTRTPDQKKTRTPDQAKKPDQAEPNKKHHHHHH

**100.**

MAKLSTDELLDAFKEMTLLELSDFVKKFEETFEVTAAAPVAVAAAGAAPAGAAVEAAEEQSEFDVILEAAGDKKIGVIKVVREIVSGLGLKEAKDLVDGAPKPLLEKVAKEAADEAKAKLEAAGATVTVKEAAAKAKFVAAWTLKAAA**PMGLP**NYYELTWDL**AAY**AYGIKSNVV**AAY**STNYYELTW**AAY**TYKNNFTLTV**AAY**TVHFHYFKL**AAY**DPTVHFHYF**AAY**NAKATYEAALKQYEA**GPGPG**KATYEAALKQYEADL**GPGPG**ATYEAALKQYEADL**GPGPG**EDEQTSIKAALAEL**GPGPG**KATYEAALKQYEAD**GPGPG**AKATYEAALKQYEA**GPGPG**PTPTPDQKKPPTRTPDKKPPTPTPDKKPTRTPDQKKTRTPDQAKKPDQAEPNKKHHHHHH
