# Supplementary material for: Design of a Novel Peptide‐Based Vaccine Targeting Streptococcus mutans SpaP Antigen for Dental Caries Prevention
Source: Int J Dent. 2026 Jun 29;2026:5545020. doi: 10.1155/ijod/5545020 (PMC13312148; doi:10.1155/ijod/5545020)
Supplement: Supplementary file 6 — Supporting Information 6 Data 6: Multiepitope vaccine sequences evaluation. [file IJOD-2026-5545020-s006.docx]

| **Sequence** | **Instability index** | **Aliphatic index** | **PI** | **Half-life (hr)** | **GRAVY** | **Vaxijen score** | **SOLpro score** | **Allergenicity** |
| --- | --- | --- | --- | --- | --- | --- | --- | --- |
| 1 | 10.85 | 67.54 | 5.88 | 30 | -0.498 | 0.8245 | Soluble: 98% | No evidence |
| 2 | 10.85 | 67.54 | 5.88 | 30 | -0.498 | 0.8287 | Soluble: 98% | No evidence |
| 3 | 10.85 | 67.54 | 5.88 | 30 | -0.498 | 0.8257 | Soluble: 98% | No evidence |
| 4 | 10.85 | 67.54 | 5.88 | 30 | -0.498 | 0.8185 | Soluble: 98% | No evidence |
| 5 | 10.85 | 67.54 | 5.88 | 30 | -0.498 | 0.8254 | Soluble: 98% | No evidence |
| 6 | 10.85 | 67.54 | 5.88 | 30 | -0.498 | 0.824 | Soluble: 98% | No evidence |
| 7 | 10.85 | 67.54 | 5.88 | 30 | -0.498 | 0.8188 | Soluble: 98% | No evidence |
| 8 | 10.85 | 67.54 | 5.88 | 30 | -0.498 | 0.8249 | Soluble: 98% | No evidence |
| 9 | 10.85 | 67.54 | 5.88 | 30 | -0.498 | 0.8227 | Soluble: 98% | No evidence |
| 10 | 10.85 | 67.54 | 5.88 | 30 | -0.498 | 0.8193 | Soluble: 98% | No evidence |
| 11 | 10.85 | 67.54 | 5.88 | 30 | -0.498 | 0.8208 | Soluble: 98% | No evidence |
| 12 | 10.85 | 67.54 | 5.88 | 30 | -0.498 | 0.8206 | Soluble: 98% | No evidence |
| 13 | 10.85 | 67.54 | 5.88 | 30 | -0.498 | 0.8197 | Soluble: 98% | No evidence |
| 14 | 10.85 | 67.54 | 5.88 | 30 | -0.498 | 0.8240 | Soluble: 98% | No evidence |
| 15 | 10.85 | 67.54 | 5.88 | 30 | -0.498 | 0.8192 | Soluble: 98% | No evidence |
| 16 | 10.85 | 67.54 | 5.88 | 30 | -0.498 | 0.8142 | Soluble: 98% | No evidence |
| 17 | 10.85 | 67.54 | 5.88 | 30 | -0.498 | 0.8191 | Soluble: 98% | No evidence |
| 18 | 10.85 | 67.54 | 5.88 | 30 | -0.498 | 0.8194 | Soluble: 98% | No evidence |
| 19 | 10.85 | 67.54 | 5.88 | 30 | -0.498 | 0.8171 | Soluble: 99% | No evidence |
| 20 | 11.06 | 67.54 | 5.88 | 30 | -0.498 | 0.8236 | Soluble: 87% | No evidence |
| 21 | 10.08 | 67.54 | 5.88 | 30 | -0.498 | 0.8261 | Soluble: 89% | No evidence |
| 22 | 10.08 | 67.54 | 5.88 | 30 | -0.498 | 0.8305 | Soluble: 94% | No evidence |
| 23 | 10.74 | 67.54 | 5.88 | 30 | -0.498 | 0.8321 | Soluble: 88% | No evidence |
| 24 | 10.74 | 67.54 | 5.88 | 30 | -0.498 | 0.837 | Soluble: 88% | No evidence |
| 25 | 10.74 | 67.54 | 5.88 | 30 | -0.498 | 0.8293 | Soluble: 88% | No evidence |
| 26 | 10.74 | 67.54 | 5.88 | 30 | -0.498 | 0.8315 | Soluble: 88% | No evidence |
| 27 | 10.74 | 67.54 | 5.88 | 30 | -0.498 | 0.8369 | Soluble: 88% | No evidence |
| 28 | 11.33 | 67.54 | 5.88 | 30 | -0.498 | 0.8233 | Souluble: 89% | No evidence |
| 29 | 11.33 | 67.54 | 5.88 | 30 | -0.498 | 0.8271 | Souluble: 89% | No evidence |
| 30 | 11.33 | 67.54 | 5.88 | 30 | -0.498 | 0.8217 | Souluble: 89% | No evidence |
| 31 | 11.33 | 67.54 | 5.88 | 30 | -0.498 | 0.8232 | Soluble: 90% | No evidence |
| 32 | 11.33 | 67.54 | 5.88 | 30 | -0.498 | 0.8281 | Soluble: 90% | No evidence |
| 33 | 11.33 | 67.54 | 5.88 | 30 | -0.498 | 0.8256 | Souluble: 89% | No evidence |
| 34 | 11.33 | 67.54 | 5.88 | 30 | -0.498 | 0.8271 | Souluble: 89% | No evidence |
| 35 | 11.33 | 67.54 | 5.88 | 30 | -0.498 | 0.8267 | Souluble: 89% | No evidence |
| 36 | 11.33 | 67.54 | 5.88 | 30 | -0.498 | 0.8179 | Souluble: 89% | No evidence |
| 37 | 11.33 | 67.54 | 5.88 | 30 | -0.498 | 0.8256 | Souluble: 89% | No evidence |
| 38 | 11.33 | 67.54 | 5.88 | 30 | -0.498 | 0.8222 | Souluble: 89% | No evidence |
| 39 | 11.33 | 67.54 | 5.88 | 30 | -0.498 | 0.8235 | Souluble: 89% | No evidence |
| 40 | 11.06 | 67.54 | 5.88 | 30 | -0.498 | 0.8207 | Soluble: 88% | No evidence |
| 41 | 11.06 | 67.54 | 5.88 | 30 | -0.498 | 0.8258 | Soluble: 88% | No evidence |
| 42 | 11.06 | 67.54 | 5.88 | 30 | -0.498 | 0.8231 | Soluble: 88% | No evidence |
| 43 | 11.06 | 67.54 | 5.88 | 30 | -0.498 | 0.8202 | Soluble: 88% | No evidence |
| 44 | 11.06 | 67.54 | 5.88 | 30 | -0.498 | 0.8184 | Soluble: 88% | No evidence |
| 45 | 11.06 | 67.54 | 5.88 | 30 | -0.498 | 0.8189 | Soluble: 88% | No evidence |
| 46 | 11.06 | 67.54 | 5.88 | 30 | -0.498 | 0.825 | Soluble: 88% | No evidence |
| 47 | 11.06 | 67.54 | 5.88 | 30 | -0.498 | 0.8184 | Soluble: 88% | No evidence |
| 48 | 11.06 | 67.54 | 5.88 | 30 | -0.498 | 0.8202 | Soluble: 88% | No evidence |
| 49 | 10.85 | 67.54 | 5.88 | 30 | -0.498 | 0.8245 | Soluble: 98% | No evidence |
| 50 | 10.85 | 67.54 | 5.88 | 30 | -0.498 | 0.8245 | Soluble: 98% | No evidence |
| 51 | 10.85 | 67.54 | 5.88 | 30 | -0.498 | 0.8245 | Soluble: 98% | No evidence |
| 52 | 10.85 | 67.54 | 5.88 | 30 | -0.498 | 0.8245 | Soluble: 98% | No evidence |
| 53 | 10.85 | 67.54 | 5.88 | 30 | -0.498 | 0.8245 | Soluble: 98% | No evidence |
| 54 | 10.85 | 67.54 | 5.88 | 30 | -0.498 | 0.8245 | Soluble: 98% | No evidence |
| 55 | 10.85 | 67.54 | 5.88 | 30 | -0.498 | 0.8245 | Soluble: 98% | No evidence |
| 56 | 10.85 | 67.54 | 5.88 | 30 | -0.498 | 0.8245 | Soluble: 98% | No evidence |
| 57 | 10.85 | 67.54 | 5.88 | 30 | -0.498 | 0.8245 | Soluble: 98% | No evidence |
| 58 | 10.85 | 67.54 | 5.88 | 30 | -0.498 | 0.8245 | Soluble: 98% | No evidence |
| 59 | 10.85 | 67.54 | 5.88 | 30 | -0.498 | 0.8245 | Soluble: 99% | No evidence |
| 60 | 10.85 | 67.54 | 5.88 | 30 | -0.498 | 0.8245 | Soluble: 98% | No evidence |
| 61 | 10.85 | 67.54 | 5.88 | 30 | -0.498 | 0.8245 | Soluble: 98% | No evidence |
| 62 | 10.85 | 67.54 | 5.88 | 30 | -0.498 | 0.8245 | Soluble: 98% | No evidence |
| 63 | 10.85 | 67.54 | 5.88 | 30 | -0.498 | 0.8245 | Soluble: 98% | No evidence |
| 64 | 10.85 | 67.54 | 5.88 | 30 | -0.498 | 0.8245 | Soluble: 98% | No evidence |
| 65 | 10.85 | 67.54 | 5.88 | 30 | -0.498 | 0.8245 | Soluble: 98% | No evidence |
| 66 | 10.85 | 67.54 | 5.88 | 30 | -0.498 | 0.8245 | Soluble: 98% | No evidence |
| 67 | 10.85 | 67.54 | 5.88 | 30 | -0.498 | 0.8245 | Soluble: 98% | No evidence |
| 68 | 10.85 | 67.54 | 5.88 | 30 | -0.498 | 0.8245 | Soluble: 98% | No evidence |
| 69 | 10.85 | 67.54 | 5.88 | 30 | -0.498 | 0.8245 | Soluble: 98% | No evidence |
| 70 | 10.85 | 67.54 | 5.88 | 30 | -0.498 | 0.8245 | Soluble: 98% | No evidence |
| 71 | 10.85 | 67.54 | 5.88 | 30 | -0.498 | 0.8245 | Soluble: 98% | No evidence |
| 72 | 10.85 | 67.54 | 5.88 | 30 | -0.498 | 0.8245 | Soluble: 98% | No evidence |
| 73 | 10.85 | 67.54 | 5.88 | 30 | -0.498 | 0.8245 | Soluble: 98% | No evidence |
| 74 | 10.85 | 67.54 | 5.88 | 30 | -0.498 | 0.8245 | Soluble: 98% | No evidence |
| 75 | 10.85 | 67.54 | 5.88 | 30 | -0.498 | 0.8245 | Soluble: 98% | No evidence |
| 76 | 10.85 | 67.54 | 5.88 | 30 | -0.498 | 0.8245 | Soluble: 98% | No evidence |
| 77 | 10.85 | 67.54 | 5.88 | 30 | -0.498 | 0.8245 | Soluble: 98% | No evidence |
| 78 | 10.85 | 67.54 | 5.88 | 30 | -0.498 | 0.8245 | Soluble: 98% | No evidence |
| 79 | 10.85 | 67.54 | 5.88 | 30 | -0.498 | 0.8245 | Soluble: 98% | No evidence |
| 80 | 10.85 | 67.54 | 5.88 | 30 | -0.498 | 0.8245 | Soluble: 98% | No evidence |
| 81 | 10.85 | 67.54 | 5.88 | 30 | -0.498 | 0.8245 | Soluble: 98% | No evidence |
| 82 | 10.85 | 67.54 | 5.88 | 30 | -0.498 | 0.8245 | Soluble: 98% | No evidence |
| 83 | 10.85 | 67.54 | 5.88 | 30 | -0.498 | 0.8245 | Soluble: 98% | No evidence |
| 84 | 10.85 | 67.54 | 5.88 | 30 | -0.498 | 0.8257 | Soluble: 98% | No evidence |
| 85 | 10.85 | 67.54 | 5.88 | 30 | -0.498 | 0.8257 | Soluble: 98% | No evidence |
| 86 | 10.85 | 67.54 | 5.88 | 30 | -0.498 | 0.8257 | Soluble: 98% | No evidence |
| 87 | 10.85 | 67.54 | 5.88 | 30 | -0.498 | 0.8257 | Soluble: 98% | No evidence |
| 88 | 10.85 | 67.54 | 5.88 | 30 | -0.498 | 0.8257 | Soluble: 98% | No evidence |
| 89 | 10.85 | 67.54 | 5.88 | 30 | -0.498 | 0.8257 | Soluble: 98% | No evidence |
| 90 | 10.85 | 67.54 | 5.88 | 30 | -0.498 | 0.8257 | Soluble: 98% | No evidence |
| 91 | 10.85 | 67.54 | 5.88 | 30 | -0.498 | 0.8257 | Soluble: 98% | No evidence |
| 92 | 10.85 | 67.54 | 5.88 | 30 | -0.498 | 0.8257 | Soluble: 98% | No evidence |
| 93 | 10.85 | 67.54 | 5.88 | 30 | -0.498 | 0.8257 | Soluble: 98% | No evidence |
| 94 | 10.85 | 67.54 | 5.88 | 30 | -0.498 | 0.8257 | Soluble: 98% | No evidence |
| 95 | 10.85 | 67.54 | 5.88 | 30 | -0.498 | 0.8257 | Soluble: 98% | No evidence |
| 96 | 10.85 | 67.54 | 5.88 | 30 | -0.498 | 0.8257 | Soluble: 98% | No evidence |
| 97 | 10.85 | 67.54 | 5.88 | 30 | -0.498 | 0.8257 | Soluble: 98% | No evidence |
| 98 | 10.85 | 67.54 | 5.88 | 30 | -0.498 | 0.8257 | Soluble: 98% | No evidence |
| 99 | 10.85 | 67.54 | 5.88 | 30 | -0.498 | 0.8257 | Soluble: 98% | No evidence |
| 100 | 10.85 | 67.54 | 5.88 | 30 | -0.498 | 0.8257 | Soluble: 98% | No evidence |
